# Preprogrammed assembly of supramolecular polymer networks via the controlled disassembly of a metastable rotaxane

**DOI:** 10.1038/s42004-022-00774-5

**Published:** 2022-11-21

**Authors:** Gosuke Washino, Miguel A. Soto, Siad Wolff, Mark J. MacLachlan

**Affiliations:** 1 Department of Chemistry, University of British Columbia, 2036 Main Mall, Vancouver, BC V6T 1Z1 Canada; 2 Stewart Blusson Quantum Matter Institute, University of British Columbia, 2355 East Mall, Vancouver, BC V6T 1Z4 Canada; 3 Bioproducts Institute, University of British Columbia, 2385 East Mall, Vancouver, BC V6T 1Z4 Canada; 4 WPI Nano Life Science Institute, Kanazawa University, Kanazawa, 920-1192 Japan

**Keywords:** Interlocked molecules, Self-assembly, Gels and hydrogels

## Abstract

In our daily life, some of the most valuable commodities are preprogrammed or preassembled by a manufacturer; the end-user puts together the final product and gathers properties or function as desired. Here, we present a chemical approach to preassembled materials, namely supramolecular polymer networks (SPNs), which wait for an operator’s command to organize autonomously. In this prototypical system, the controlled disassembly of a metastable interlocked molecule (rotaxane) liberates an active species to the medium. This species crosslinks a ring-containing polymer and assembles with a reporting macrocycle to produce colorful SPNs. We demonstrate that by using identical preprogrammed systems, one can access multiple supramolecular polymer networks with different degrees of fluidity (μ^*^ = 2.5 to 624 Pa s^-1^) and color, all as desired by the end-user.

## Abstract (web summary)

Preassembled materials are ubiquitous in our everyday life due to their readiness and functionality; an end-user simply follows instructions to assemble them and harness function. Here, metastable rotaxanes are utilized to approach preassembled materials: a multicomponent, preprogrammed system can be conveniently (*via* heating) transformed into colorful polymer networks at the end-user’s will.

## Introduction

Since the introduction of molecular meccano^1, 2^ a myriad of mechanically interlocked molecules (MIMs) has been reported^3^, from rotaxanes^4–6^ and catenanes^7–9^ to other fascinating superstructures^10–14^. The assembly of MIMs has been of particular interest in recent years^15^ (new syntheses^16^ and emerging host and guest elements^17^), which is in stark contrast to the few studies that have explored their disassembly. These examples include the delivery of small molecules to a medium^18–21^ and the degradation of crosslinked polymers using metastable rotaxanes^22–24^.

**Fig. 1:**
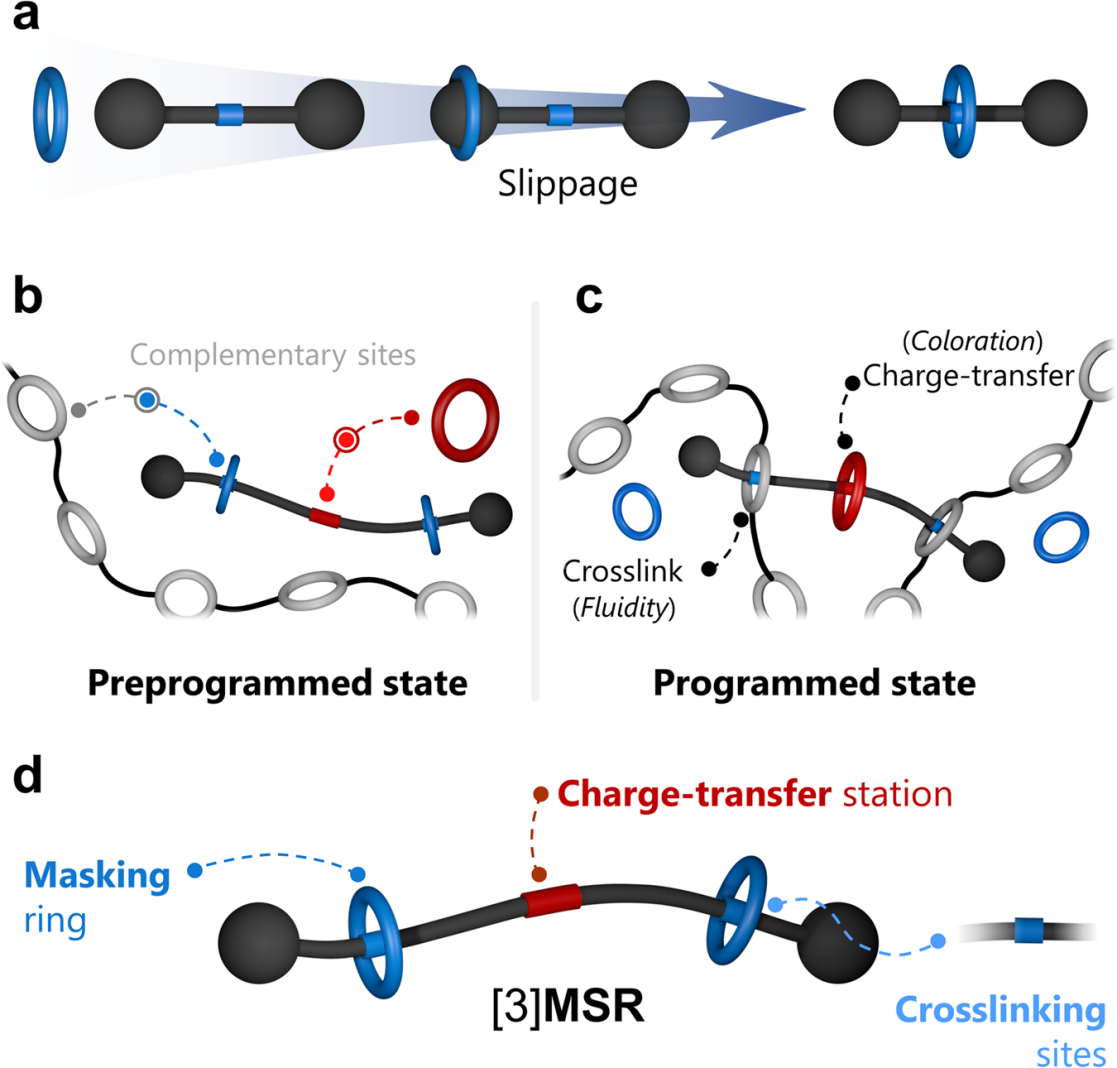
Metastable rotaxanes to produce supramolecular gels with a color indicator. Cartoon representation of **a** rotaxane assembly through the slippage approach, **b** a preprogrammed system containing complementary elements, **c** a programmed self-assembled supramolecular network, and **d** [3]**MSR** comprising one dumbbell molecule and two rings.

Stoddart et al. introduced the synthesis of metastable rotaxanes—also known as kinetically stable pseudorotaxanes—using the slipping approach, i.e. threading one ring onto a preformed dumbbell-like molecule using specific stimuli (Fig. 1a)^25–27^. At elevated temperatures, for example, a ring slips over one of the dumbbell’s stoppers and both components—ring and dumbbell—link together as the temperature is reduced. With the right stimulus, the kinetically trapped components in a metastable rotaxane can be unlinked and consequently released to the medium.

In our view, the disassembly of rotaxanes could trigger a series of sequenced self-assembly processes to yield, e.g., other MIMs, molecular devices, or nanostructured materials. As a proof-of-principle, we envisioned a multicomponent system in which the scaffolds to produce a colored supramolecular polymer network (SPN)^28^ coexist in a medium, but are unable to interact (preprogrammed state, Fig. 1b). These subcomponents wait for the end-user’s command (programming stage, see Fig. 1c) to initiate their assembly.

We designed a prototypical metastable [3]rotaxane ([3]**MSR**) that contains a dumbbell (crosslinker) threaded by two rings (Fig. 1d). The dumbbell has three recognition sites, two near the ends to crosslink a ring-containing polymer, and a central station to produce a colorful charge-transfer (CT) complex. In its rotaxanated form, the crosslinker cannot be threaded by other species in solution: it is masked or *protected* from further self-assembly processes. The end-user’s command will cause a series of sequenced events: the release of the crosslinker, its assembly with a reporting macrocycle via CT, crosslinking of the ring-containing polymer, and the production of a colored gel material.

## Results and discussion

**Fig. 2:**
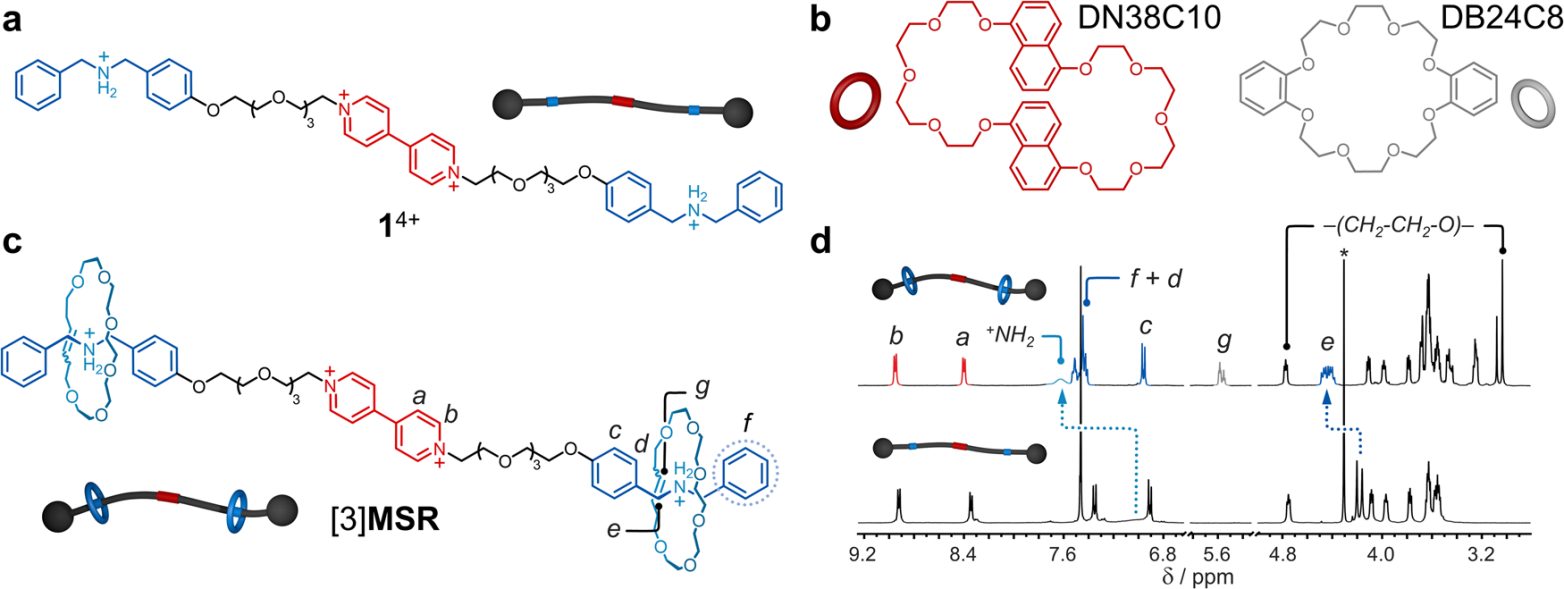
NMR data for [3]MSR and the axle. Chemical structures and schematic representations of **a**, **b** the employed molecular components and **c** the assembled metastable [3]rotaxane [3]**MSR**. **d** Partial ^1^H NMR spectra (CD_3_CN, 400 MHz) of [3]**MSR** (top) and **1**^4+^ (bottom) prepared at 3.5 × 10^-3^ M. *Denotes MeNO_2_.

To prove our concept, crosslinker **1**^4+^ (PF_6_^−^ salt, Fig. 2a) was prepared (four steps, 35% overall yield) and characterized by high-resolution mass spectrometry (HRMS) and nuclear magnetic resonance (NMR) spectroscopy, Supplementary Figs. 1‒9. We selected **1**^4+^ among other prospective crosslinkers (Supplementary Fig. 10) based on the following experimental evidence (Supplementary Figs. 11‒20): (i) its bipyridinium (BIPY) core station interacts with the reporting macrocycle DN38C10 (Fig. 2b)^29^ to give a colored CT complex. (ii) Both dibenzylammonium (DBA) stations assemble with the 24-membered crown ether DB24C8^30^ (embedded in the ring-containing polymer of choice, vide infra), which ensures that **1**^4+^ will act as a crosslinker. (iii) DN38C10 and DB24C8 self-sort along the three stations contained in **1**^4+^ (DN38C10/BIPY and DB24C8/DBA), meaning that crosslinking and CT complex formation will occur simultaneously in the programmed system. In addition, the DBA motifs in **1**^4+^ are known to be excellent scaffolds to construct metastable rotaxanes that disassemble irreversibly upon thermal stimulation^31^.

Crosslinker **1**^4+^ was used to synthesize what was anticipated to behave as a metastable rotaxane, [3]**MSR**. Ring-closing metathesis of pentaethylene glycol dibut-4-enyl ether, in the presence of **1**[PF_6_]_4_ and Grubbs catalyst II^32^, yielded [3]**MSR** bearing one [22]crown-6 ether (**22C6**) per DBA unit (Fig. 2c)^33^. The purity and interlocked nature of [3]**MSR** were assessed by 1D and 2D NMR spectroscopy (Supplementary Figs. 21‒27). The ^1^H NMR spectrum showed resonances ^*+*^*NH*_*2*_ and *e* visibly shifted (e.g., Δδ_*e*_ = 0.25 ppm) with respect to those of free **1**^4+^ (Fig. 2d). The observed downfield shifts suggest strong hydrogen bonding interactions between the **22C6** rings and the DBA stations. NMR spectroscopy also revealed the presence of both *cis* (7%) and *trans* (93%) isomers of the olefin-containing **22C6** rings. HRMS confirmed the formation of [3]**MSR** through the molecular ion [**1** + 2(**22C6**) + 2(PF_6_)]^2+^, detected at *m*/*z* = 914.4279 (calc. 914.4276).

**Fig. 3:**
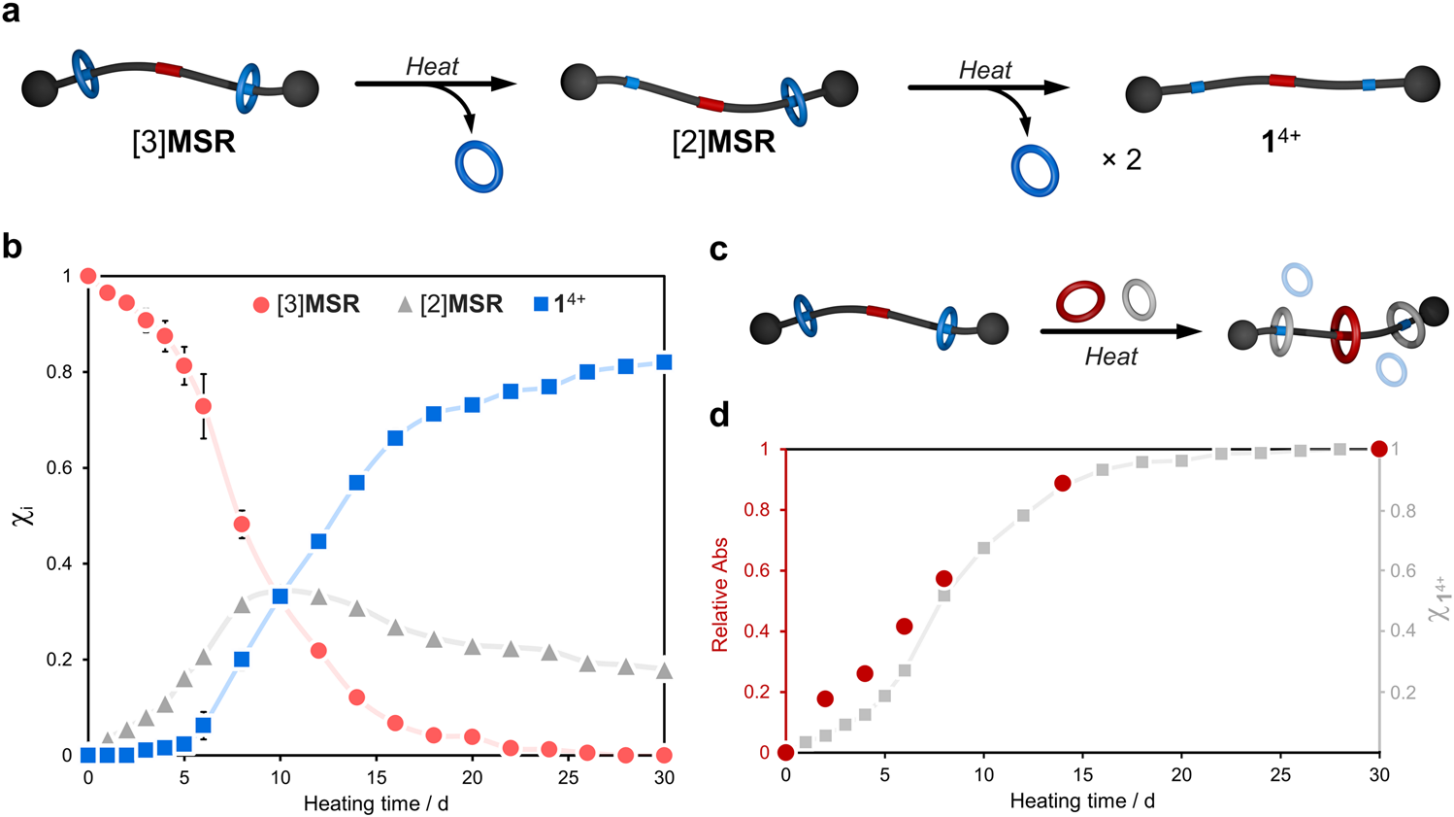
Dissociation process for [3]MSR. **a** Schematic representation of the stepwise disassembly of [3]**MSR**. **b** Speciation plot showing the appearance of [2]**MSR** and **1**^4+^ to the detriment of [3]**MSR**. Data extracted from ^1^H NMR spectra (Supplementary Note 1). Error bars correspond to the standard deviation of three independent experiments. **c** Cartoon representation of a two-by-three ring-exchange involving [3]**MSR**, DB24C8, and DN38C10. **d** Relative absorbance changes at the CT band (486 nm) throughout the ring-exchange process (red). The gradual release of **1**^4+^ via the disassembly of [3]**MSR** is shown here for comparison (gray).

Compound [3]**MSR** is stable in acetonitrile and acetonitrile/chloroform mixtures; no disassembly was observed after 30 d at room temperature (Supplementary Fig. 31). However, heating the sample at 70 °C (CD_3_CN/CDCl_3_, 1/1, v/v) for 30 days rendered the loose components in solution. This experiment was monitored by ^1^H NMR spectroscopy for 30 days (Fig. 3a and Supplementary Figs. 32‒38). After 1 day of heating, a new set of resonances was visible in the ^1^H NMR spectrum; signals at 5.54 and 5.48 ppm matched well with protons *g (cis*/*trans)* observed in a pure sample of **22C6** (Supplementary Fig. 32); resonances at 6.53 (*c*) and 7.23 (*d*) ppm were also indicative of disassembled **1**^4+^ in solution. In addition, we detected a [2]rotaxane intermediate ([2]**MSR**) through resonances at 6.94 and 6.72 ppm. The presence of [2]**MSR** was further confirmed by 2D NMR spectroscopy and HRMS (Supplementary Figs. 35,36).

During the 30-day heating experiment, there was a clear trend that we mapped through the integration of the ^1^H NMR spectra (Supplementary Note 1). As the speciation plot in Fig. 3b shows, the concentration of [3]**MSR** dropped by 10% after 3 days and 50% after 8 days, then reached a plateau after ca. 28 days. With the disappearance of [3]**MSR**, the appearance of [2]**MSR** (peaking at 34% in 10 days) and **1**^4+^ was evident. After 30 days of heating, we registered an estimated composition of 0.2 ± 0.2% [3]**MSR**, 18.0 ± 0.2% [2]**MSR**, and 82.0 ± 0.5% **1**^4+^. A mass spectrum of the disassembled material revealed the presence of [**1** + **22C6**]^4+^ (*m*/*z* = 305.1818, calc. 305.1804) along with the free species [**1** – 2H]^2+^ (*m*/*z* = 450.2515, calc. 450.2513) and [**22C6** + NH_4_]^+^ (*m*/*z* = 336.2383, calc. 336.2381). We proved the disassembly of [3]**MSR** to be irreversible; detailed information is in Supplementary Fig. 39.

In a set of control experiments (Supplementary Figs. 40–42), we demonstrated that [3]**MSR** is masked and cannot be threaded by DN38C10 or DB24C8. In contrast, ring-exchange processes occur when thermal stimulation is applied (Supplementary Figs. 43–55). That is, heating a solution of [3]**MSR** in the presence of DN38C10, DB24C8, or a mixture of DN38C10/DB24C8 leads to two-by-one, two-by-two, and two-by-three ring-exchange processes (Fig. 3c), respectively. For instance, the gradual release of **1**^4+^, in the presence of DN38C10, allowed for the threading of the reporting macrocycle onto the axle, producing the expected naphthalene/BIPY CT complex. By UV-vis spectroscopy, we detected an increase in absorbance (λ_CT_ = 486 nm) with heating time, reaching saturation in ca. 14 days (Supplementary Fig. 52 and Supplementary Note 2). The CT complex emerged at a rate that correlated well with the standalone release of **1**^4+^, see Fig. 3d.

**Fig. 4:**
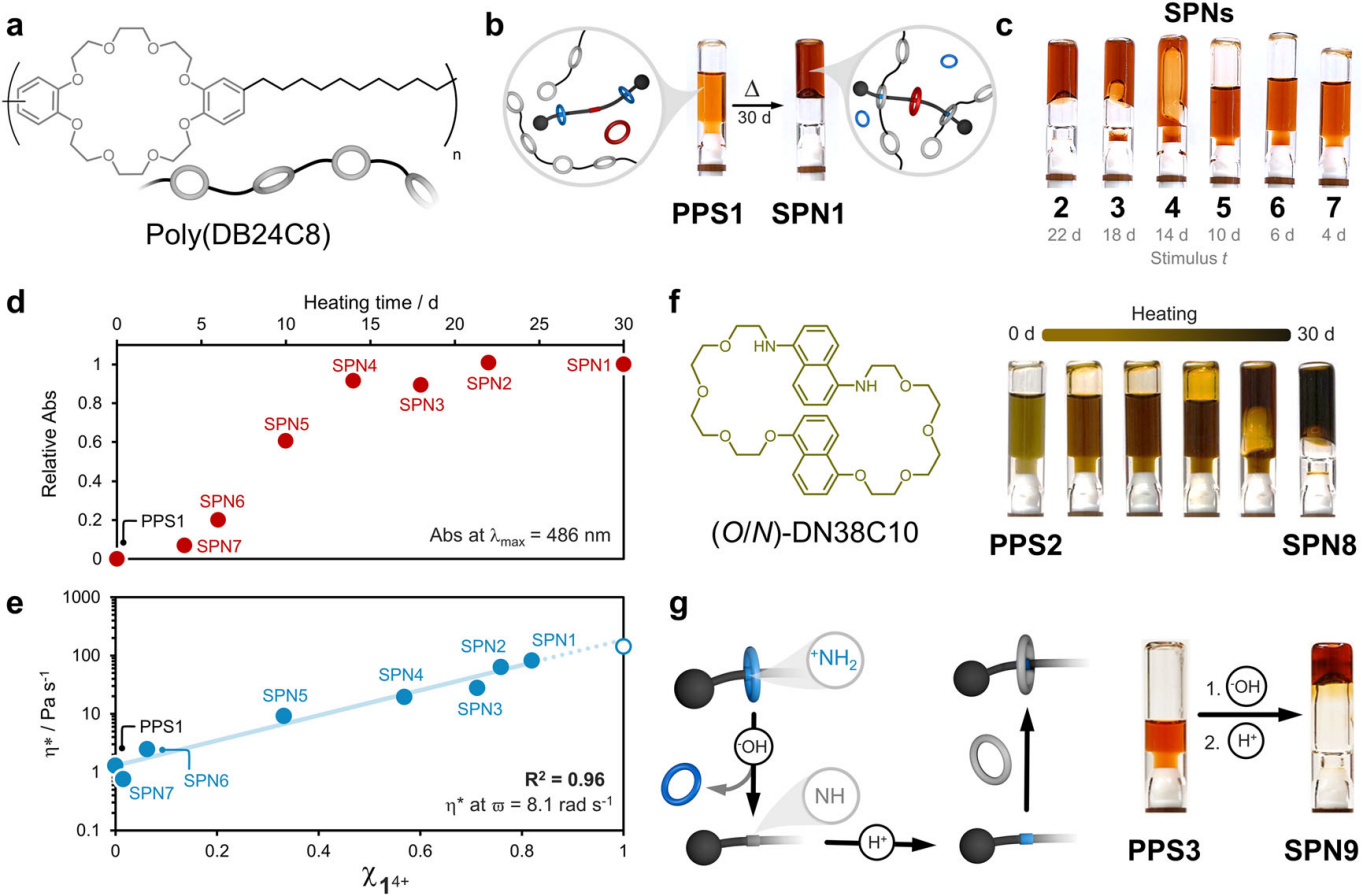
Supramolecular gels formed from the controlled dissociation of a metastable rotaxane. **a** Chemical structure and cartoon of polymer poly(DB24C8)^22^. **b** Thermally controlled disassembly of the preprogrammed system **PPS1** and its assembly into **SPN1**. **c** SPNs **2**–**7**, all prepared in (CHCl_3_/CH_3_CN, 1/1, v/v) and heated (70 °C) for an arbitrary period (see Supplementary Methods); [[3]**MSR**] = 1.3 × 10^−2^ M, [DN38C10] = 1.3 × 10^−2^ M, and [poly(DB24C8)] = 3.1 × 10^−1^ M. Photographs were taken upon equilibration at room temperature. **d** Relative absorbance changes for the CT band (486 nm) throughout the heating progress; all samples were diluted to 5 × 10^−3^ M (CHCl_3_/CH_3_CN, 1/1, v/v) prior to collecting the corresponding spectrum. **e** Complex viscosity (η*) changes over the thermal treatment of a preprogrammed system (measured at 15 °C). Fitting to a logarithmic function is shown as a blue line. The open symbol represents a reference sample prepared with [**1**[PF_6_]_4_] = 1.3 × 10^−2^ M, [DN38C10] = 1.3 × 10^−2^ M, [**22C6**] = 2.6 × 10^−2^ M, and [poly(DB24C8)] = 3.1 × 10^−1^ M. **f** Alternative approach to tune the apparent color of preprogrammed SPNs. **PPS2** prepared in CHCl_3_/CH_3_CN, 1/1, v/v, its evolution was imaged at different stages (six shown) after equilibrating to room temperature; [[3]**MSR**] = 1.3 × 10^−2^ M, [(O/N)-DN38C10] = 1.3 × 10^−2^ M, and [poly(DB24C8)] = 3.1 × 10^−1^ M. **g** Acid/base controlled self-assembly of **PPS3**; [[3]**MSR**] = 2.0 × 10^−2^ M, [DN38C10] = 2.0 × 10^−2^ M, and [poly(DB24C8)] = 3.0 × 10^−1^ M in CHCl_3_/CH_3_CN, 1/1, v/v. The schematic shows the expected transformation at the acid/base-responsive station (DBA) in **1**^4+^. Scale bars represent 1 cm.

Next, we tested the activity of [3]**MSR** in a preprogrammed state, i.e., a system containing [3]**MSR**, DN38C10, and the ring-containing polymer poly(DB24C8)^34–36^, see Fig. 4a (synthesis and characterization in Supplementary Methods). After mixing [3]**MSR** (1.3 × 10^−2^ M) with DN38C10 (1 equiv) and poly(DB24C8) (24 equiv, see Supplementary Fig. 59), a light orange color appeared (Fig. 4b), which was attributed to weak CT from the electron-rich DN38C10 ring to the BIPY unit in **1**^4+^ (unthreaded). A viscosity increase was not evident, and no further changes were observed after storing the sample for 30 days at room temperature. This indicated the successful creation of a preprogrammed state (**PPS1**).

The sample was then heated for 30 days at 70 °C. During the equilibration of the system to room temperature, there was a clear color change from orange to dark red, accompanied by the gelation of the system (Fig. 4b). These observations indicated that the reporter DN38C10 threaded **1**^4+^ to display CT (λ_CT_ = 486 nm), while the DBA stations crosslinked poly(DB24C8) to render supramolecular polymer network **SPN1**. The resulting material showed a clear increase in complex viscosity (η*), from 1.3 Pa s^-1^ (**PPS1**) to 82 Pa s^-1^ (**SPN1**) at ω = 8.1 rad s^−1^, which further confirmed the crosslinking of poly(DB24C8).

We proved that the **PPS1** can be programmed into multiple polymer networks, **SPN1**–**SPN7** (see Fig. 4c and Supplementary Fig. 60), by controlling heating time. **SPN4**, for instance, was achieved after 14 days of heating and showed a distinct set of properties with respect to **SPN1**, with a 10% decrease in absorbance (Fig. 4d), and η* of 19.3 Pa s^−1^. We were able to tune the η* values of the SPNs from 2.5 to 82 Pa s^−1^ (Fig. 4e) by controlling the stimulus duration (Supplementary Figs. 60–63). Likewise, PPSs prepared with different ratios of components were also heat-programmable, showing η* values that increased linearly from 1.9 Pa s^−1^ for the corresponding PPS to 624 Pa s^−1^ for the stiffest SPN (*R*^2^ = 0.97), see Supplementary Fig. 63. It is worth mentioning that the color intensity and η* are inherently connected, and the end-user can estimate η* of a programmed SPN by determining the absorbance intensity of the corresponding CT band.

Our system was designed so that the CT complex within the SPNs would give an intense red color to the material. This was adjusted with ease by switching the π-electron-rich rings contained in DN38C10. The parent macrocycle (*O*/*N*)-DN38C10^37^, comprising dioxy- and diaminonaphthalene moieties, rendered **SPN8** with distinct color (Fig. 4f). The thermal activation of **PPS2** containing (*O*/*N*)-DN38C10, changed from a light green solution to a dark brown gel, which demonstrated the modularity of our approach.

The proof-of-concept presented here aims to show that complex materials can be preprogrammed for an end-user, who can initialize and finalize the assembly at their own discretion, using a non-invasive stimulus (heat). It is known that metastable rotaxanes based on DBA and **22C6** disassemble irreversibly upon deprotonation^18^. To demonstrate the versatility of our system, we investigated the activation of **PPS3** using an invasive stimulus (base/acid, Fig. 4g). A solution containing [3]**MSR** (2.0 × 10^−2^ M), DN38C10 (1 equiv) and poly(DB24C8) (15 equiv) was treated with 1.1 equiv of NaOH_(aq)_ (10 M). This caused the deprotonation of the DBA moieties followed by disassembly. Gel formation was not observed as the DBA stations were disabled upon deprotonation. After adding 1.2 equiv of HBF_4_ (2 M in diethyl ether), an intensely red-colored gel formed, confirming the assembly of **SPN9**. It is noteworthy that although the whole process (disassembly/SPN formation) takes less than 10 min., the base/acid-activated approach is invasive and requires preparation and accurate addition of acidic and basic reagents, which might be disadvantageous when compared to the thermally activated process.

## Conclusions

We demonstrated that metastable mechanically interlocked molecules can serve as scaffolds to preprogram the assembly of supramolecular materials, namely SPNs. The end-user can program (assemble) SPNs according to the desired fluidity and coloration by simply applying non-invasive (thermal) and invasive (acid/base) stimuli. To the best of our knowledge, this is the only example that shows the use of metastable MIMs to pre-assemble materials that await an operator’s command to organize^38–40^. We anticipate that this approach will be useful in the development of intricate preprogrammed systems that could lead to the assembly of MIMs, molecular devices, and other self-assembled materials.

## Methods

### Materials

Commercially available chemicals were purchased from Sigma-Aldrich, Tokyo Chemical Industry (TCI), and Oakwood Chemical, and used as received. Dry dichloromethane (CH_2_Cl_2_) was collected from an Inert PureSolv MD5 purification system, whereas acetonitrile (CH_3_CN) and chloroform (CHCl_3_) were freshly dried with activated 3 Å molecular sieves. Deuterated solvents (CD_3_CN and CDCl_3_) were purchased from Cambridge Isotope Laboratories and freshly dried using activated 3 Å molecular sieves. Flash column chromatography was carried out using SiliCycle (230–400 mesh) silica gel as the stationary phase.

### Physical techniques

Nuclear magnetic resonance (NMR) experiments were recorded on Bruker AVIII HD 400 MHz and Bruker Avance 400 MHz spectrometers; ^1^H and ^13^C NMR chemical shifts (δ) are given in parts per million (ppm) relative to tetramethylsilane as referenced with the residual solvent signal. *J* values are reported in Hz, and signal multiplicity is denoted as *s* (singlet), *d* (doublet), *t* (triplet), *dd* (doublet of doublet), *m* (multiplet), and *br* (broad signal). UV-vis spectra were recorded on a Cary 5000 UV-vis-NIR spectrometer, employing 1 mm pathlength quartz cuvettes. Electrospray ionization high-resolution mass spectra (ESI-HRMS) were recorded on an ESI-TOF Waters Micromass LCT spectrometer. Rheology tests were carried out on Anton Paar Modular Compact Rheometer MCR 502 with a cone plate geometry (diameter 25 mm, cone angle 1°).

### Synthesis of [3]rotaxane ([3]MSR)

Compound **1**^4+^ (0.75 g, 0.50 mmol) and pentaethylene glycol dibut-4-enyl-ether (0.62 g, 1.80 mmol) were dissolved in a mixture of CH_3_CN and CHCl_3_ (15 mL, 1:1, v/v), and stirred at room temperature overnight. Both solvents were evaporated under a vacuum, keeping the temperature below 30 °C. A solution of the residual orange oil, prepared in dry CH_2_Cl_2_ (500 mL), was loaded with Grubbs catalyst second generation (91.0 mg, 0.11 mmol) under the protection of an N_2_ atmosphere, and heated at 45 °C for 60 h. After cooling down to room temperature, the reaction was quenched with ethyl vinyl ether (2 mL), followed by rotary evaporation (maintaining *T* below 30 °C). Column chromatography (SiO_2_, CH_2_Cl_2_/CH_3_OH = 92:8 (v/v), R_f_ = 0.33) yielded [3]**MSR** in 28% yield as a dark brown oil. Postprocessing of [3]**MSR** allowed us to obtain a less colored material, which was relevant for our following studies. Rotaxane [3]**MSR** (484 mg) was dissolved in CH_3_CN (10 mL) and stirred with H_2_O_2_ aq (30%, 20 mL) at room temperature for 2 h, followed by evaporation of CH_3_CN by rotary evaporation. The residue was extracted with CH_2_Cl_2_ (4 × 5 mL), washed with water (2 × 10 mL), and then dried over Na_2_SO_4_. After evaporating the organic solvents, [3]**MSR** was isolated as an orangish-brown glassy solid (400 mg, 82% yield). This treatment allowed the removal of the remaining traces of coloring Ru-containing species.

### Preparation of preprogrammed systems

All samples were prepared combining [3]**MSR**, DN38C10, and poly(DB24C8) in dry CH_3_CN/CHCl_3_ (1:1, v/v). Two sets of materials were preassembled containing the following ratio of components: [3]**MSR**:DN38C10:poly(DB24C8) = 1:1:24 (12.5 mM, *system 1*) and 1:1:2 (50 mM, *system 2*). Eight and six independent samples were prepared for each system, respectively. All samples (free-flowing solutions with light orange color) were transferred and stored in glass vessels equipped with J. Young valves, under the protection of N_2_.

### Programming stage

Samples belonging to *system 1* were heated for 0 (control), 4, 6, 10, 14, 18, 22, and 30 days at 70 °C, while the six samples of *system 2* were treated for 0 (control), 10, 16, 20, 26, and 30 days at the same temperature. In all cases, the starting solutions were low-viscosity, free-flowing liquids before heating.

### Rheology measurements

All samples were analyzed by dynamic oscillatory shear measurements (25 mm diameter parallel cone plates with a 100 μm gap) from 1 to 100 rad s^−1^ at 15 °C under a strain of 1%.

## Supplementary information

Supplementary Information

## Data Availability

All data analyzed and generated throughout this study are included in this article and its accompanying Supplementary Information.
