# Supplementary material for: Preprogrammed assembly of supramolecular polymer networks via the controlled disassembly of a metastable rotaxane
Source: Commun Chem. 2022 Nov 21;5:155. doi: 10.1038/s42004-022-00774-5 (PMC9814676; doi:10.1038/s42004-022-00774-5)
Supplement: Supplementary file 1 — Supplementary Information [file 42004_2022_774_MOESM1_ESM.pdf]

Supplementary Information

Preprogrammed assembly of supramolecular polymer networks via the controlled disassembly of a metastable rotaxane

Gosuke Washino,[a+] Miguel A. Soto,[a+] Siad Wolff,[a] and Mark J. MacLachlan[abcd*]

[a] Department of Chemistry, University of British Columbia, 2036 Main Mall, Vancouver, British Columbia V6T 1Z1 Canada

[b] Stewart Blusson Quantum Matter Institute, University of British Columbia, 2355 East Mall, Vancouver, BC, V6T 1Z4 Canada

[c] Bioproducts Institute, University of British Columbia, 2385 East Mall, Vancouver, BC V6T 1Z4 Canada

[d] WPI Nano Life Science Institute, Kanazawa University, Kanazawa, 920-1192 Japan

[+] These authors contributed equally to this work

Table of Contents

Supplementary methods 3

General 3

Synthesis scheme 4

Benzaldehyde precursor **3** 5

Dialdehyde **2** 5

Guest molecule $\mathbf{1}^{4+}$ 9

Prospective dumbbell molecules 12

Pseudorotaxane Formation 12

Characterization of [2]PR [$\mathbf{1}^{4+} \subset$ DN38C10] 13

Characterization of [3]PR [$\mathbf{1}^{4+} \subset (DB24C8)_2$] 19

Characterization of [4]PR [DN38C10 $\supset \mathbf{1}^{4+} \subset (DB24C8)_2$] 23

Metastable [3]rotaxane ([3]MSR) 27

[22]crown-6 ether (22C6) 32

Stability test of [3]**MSR** at room temperature 34

Dissociation tests of [3]**MSR** 35

Supplementary note 1 41

Stability test of dissociation process of [3]**MSR** 47

Ring-exchange from metastable rotaxane to pseudorotaxane 49

Ring-exchange from [3]**MSR** to [2]pseudorotaxane 49

Ring-exchange from [3]**MSR** to [3]pseudorotaxane 52

Ring-exchange from [3]**MSR** to [4]pseudorotaxane 56

Analysis of color change over transformation from [3]**MSR** to [4]pseudorotaxane 62

Supplementary note 2 63

Synthesis and characterization of poly(DB24C8) 69

Gelation of poly(DB24C8) with $\mathbf{1}^{4+}$ 71

Preparation of preprogrammed supramolecular network samples 71

Analysis of viscosity change over transformation from [3]**MSR** to [4]pseudorotaxane 71

Supplementary references 76

Supplementary methods
General

All commercially available chemicals were purchased from Sigma-Aldrich, Tokyo Chemical Industry (TCI), and Oakwood Chemical, and used as received. Dry dichloromethane (CH_2Cl_2) was collected from an Inert PureSolv MD5 purification system, whereas acetonitrile (CH_3CN) and chloroform ($CHCl_3$) were freshly dried with activated 3 Å molecular sieves. Deuterated solvents (CD_3CN and $CDCl_3$) were purchased from Cambridge Isotope Laboratories and freshly dried using activated 3 Å molecular sieves.

Flash column chromatography was carried out using SiliCycle (230–400 mesh) silica gel as the stationary phase. Nuclear magnetic resonance (NMR) experiments were recorded on Bruker AVIII HD 400 MHz and Bruker Avance 400 MHz spectrometers; ^{1}H and ^{13}C NMR chemical shifts (δ) are given in parts per million (ppm) relative to tetramethylsilane as referenced with the residual solvent signal. *J* values are reported in Hz, and signal multiplicity is denoted as s (singlet), d (doublet), t (triplet), dd (doublet of doublet), m (multiplet), and br (broad signal). UV-vis spectra were recorded on a Cary 5000 UV-vis-NIR spectrometer, employing 1 mm pathlength quartz cuvettes. Electrospray ionization - high-resolution mass spectra (ESI-HRMS) were recorded on an ESI-TOF Waters Micromass LCT spectrometer. MALDI-TOF mass spectrometry experiments were performed using a Bruker Autoflex. GPC data were collected on a Malvern Omnisec chromatographer equipped with two T6000M columns arranged in series; the experiment was performed at a flow rate of 1 mL min^{-1} at 35 °C in THF, and using a refractive index detector. Rheology tests were carried out on Anton Paar Modular Compact Rheometer MCR 502 with a cone plate geometry (diameter 25 mm, cone angle 1º).

Compounds tetraethylene glycol monotosylate,[1] 4-(2-(2-(2-(2-hydroxyethoxy)ethoxy)ethoxy)-ethoxy)-benzaldehyde,[2] pentaethylene glycol di-but-4-enyl ether,[3] bis-1,5-dioxynaphtho[38]crown-10 (**DN38C10**),[4],[5] dibenzo[24]crown-8-containing polymer (poly(**DB24C8**)),[6] and the modified dinaphtho [38]crown-10 host ((*O/N*)-**DN38C10**)[7] were synthesized following reported methodologies; all spectroscopic characterization matched with the published data.

Synthesis scheme

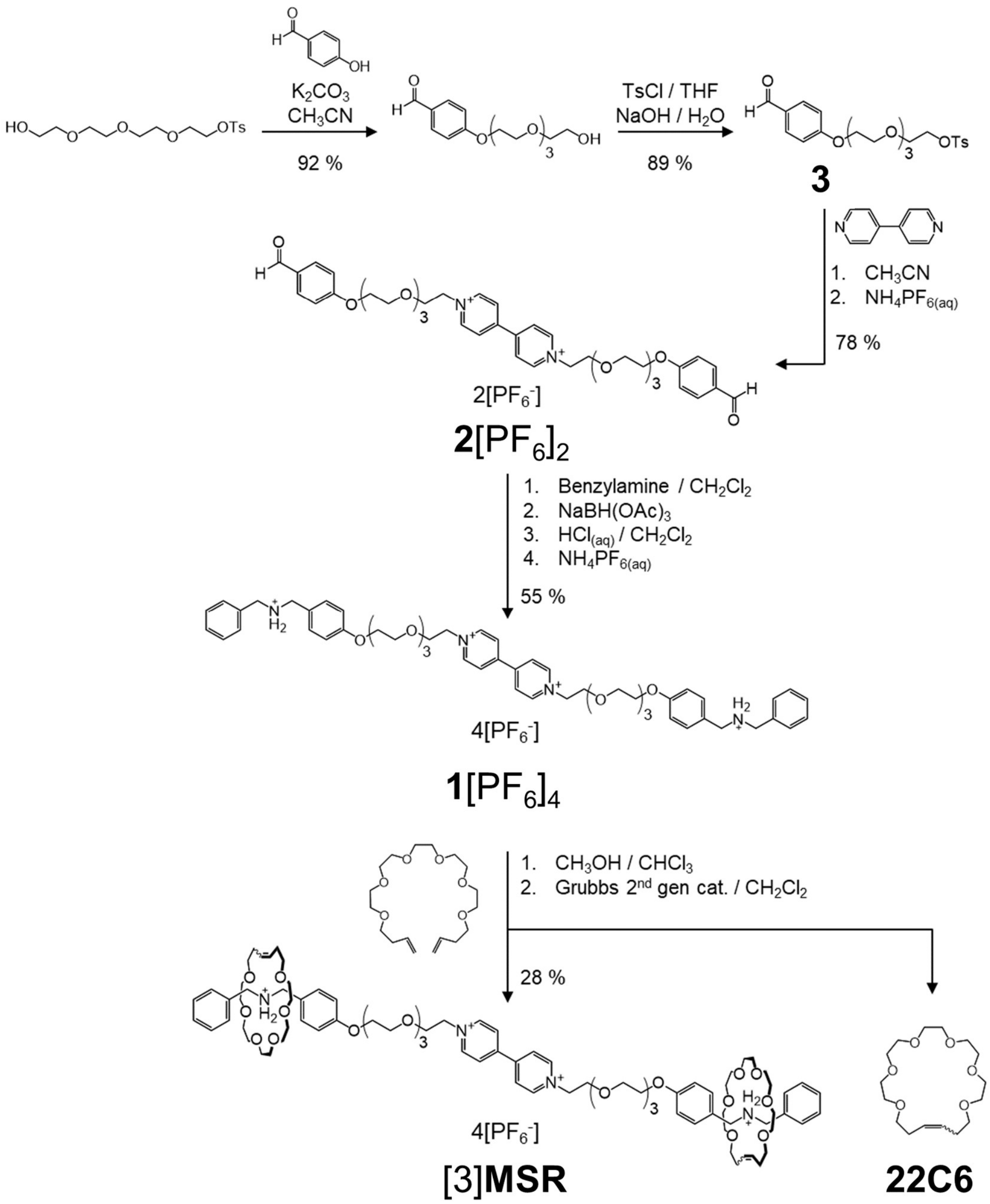

Supplementary Scheme 1. Synthesis of the guest molecule $\mathbf{1}^{4+}$, [3]**MSR** and **22C6**

Benzaldehyde precursor 3

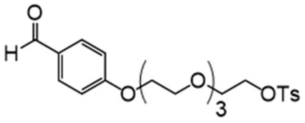

Supplementary Figure 1. Chemical structure of **3**

The title compound was synthesized following a procedure adapted from the literature.[2] A solution of sodium hydroxide (4.1g, 100 mmol) in water (50 mL) was added to a solution of 4-(2-(2-(2-(2-hydroxyethoxy)ethoxy)ethoxy)ethoxy)benzaldehyde (11.9 g, 40.0 mmol) in THF (60 mL). The mixture was cooled down to 0 ºC, and then a solution of *p*-toluenesulfonyl chloride (7.6 g, 40.0 mmol) in THF (60 mL) was added over 30 min while keeping the temperature below 5 ºC. After stirring at 0 ºC for 3 h, the reaction was poured into ice water (100 mL). The organic layer was extracted with CH_2Cl_2 (3 × 60 mL). The combined organic layers were washed with brine (2 × 50 mL) and then dried over Na_2SO_4, followed by rotary evaporation, which yielded **3** as a pale-yellow oil (16.1 g, 35.6 mmol, 89% yield). The ^1H NMR spectrum matched with the reported data[2].

Dialdehyde 2

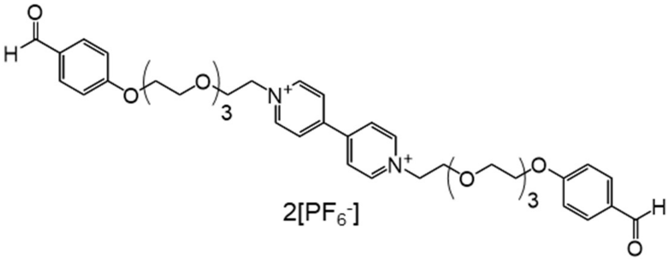

Supplementary Figure 2. Chemical structure of **2**$[PF_6]_2$

A solution of **3** (16.1 g, 35.6 mmol) and 4, 4'-bipyridyl (0.95 g, 6.1 mmol) in CH_3CN (50 mL) was heated under reflux for 6 days. After cooling down to room temperature, the solvent was evaporated under vacuum to afford an orange oil, which was dissolved in CH_2Cl_2 (40 mL). The resulting solution was stirred with brine (50 mL) at room temperature to exchange anions from TsO^- to Cl^-. Stirring continued until a third (deep orange) layer appeared between the upper aqueous phase and the bottom light-orange organic layer. The new phase consisted of **2**$[Cl]_2$ was separated and stirred with saturated aqueous NH_4PF_6 (6 mL) at room temperature for 30 min, which yielded a deep orange oil. **2**$[PF_6]_2$ was extracted from this oil with CH_2Cl_2 (220 mL), and then the combined solutions were washed with water (2 × 30 mL). The organic layer was dried over Na_2SO_4 and evaporated, the resulting brown oil was washed with $CHCl_3$ (20 mL) to remove all organic byproducts. Evaporation under reduced

pressure afforded **2**[PF_6]$_2$ as a deep orange oil (4.7 g, 4.7 mmol, 78% yield). ^{1}H NMR (400 MHz, CD_3CN) δ (ppm) = 9.86 (s, 2H), 8.95 (d, J = 6.9 Hz, 4H), 8.30 (d, J = 6.7 Hz, 4H), 7.78 (d, J = 8.8 Hz, 4H), 6.96 (d, J = 8.7 Hz, 4H), 4.77 (t, J = 4.6 Hz, 4H), 4.18 (t, J = 4.4 Hz, 4H), 4.00 (t, J = 4.6 Hz, 4H), 3.83 (t, J = 4.4 Hz, 4H), 3.72 – 3.55 (m, 16H). ^{13}CNMR (100 MHz, CD_3CN) δ (ppm) = 191.0, 163.6, 149.7, 146.3, 131.7, 130.2, 126.5, 114.8, 70.4, 70.4, 70.0, 70.0, 69.0, 68.7, 68.0, 61.6. ESI-HRMS: m/z calculated for [**2** + PF_6]$^+$ $C_{40}H_{50}F6N_2O_{10}P$, 863.3102; found, 863.3099; relative error -0.3 ppm.

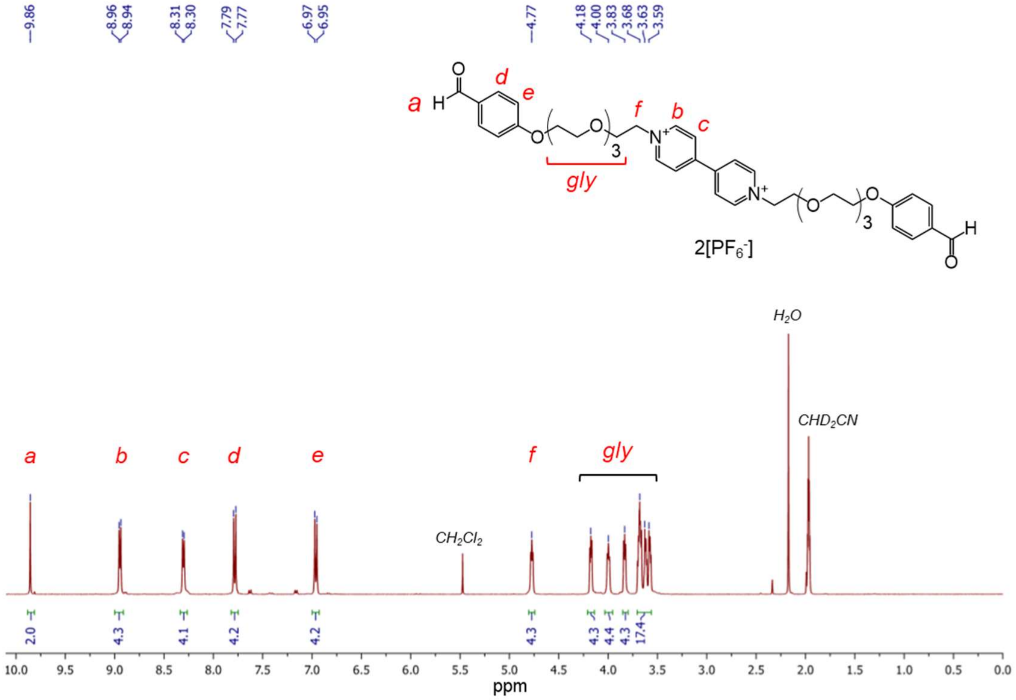

Supplementary Figure 3. ^{1}H NMR spectrum (400 MHz, CD_3CN) of **2**[PF_6]$_2$

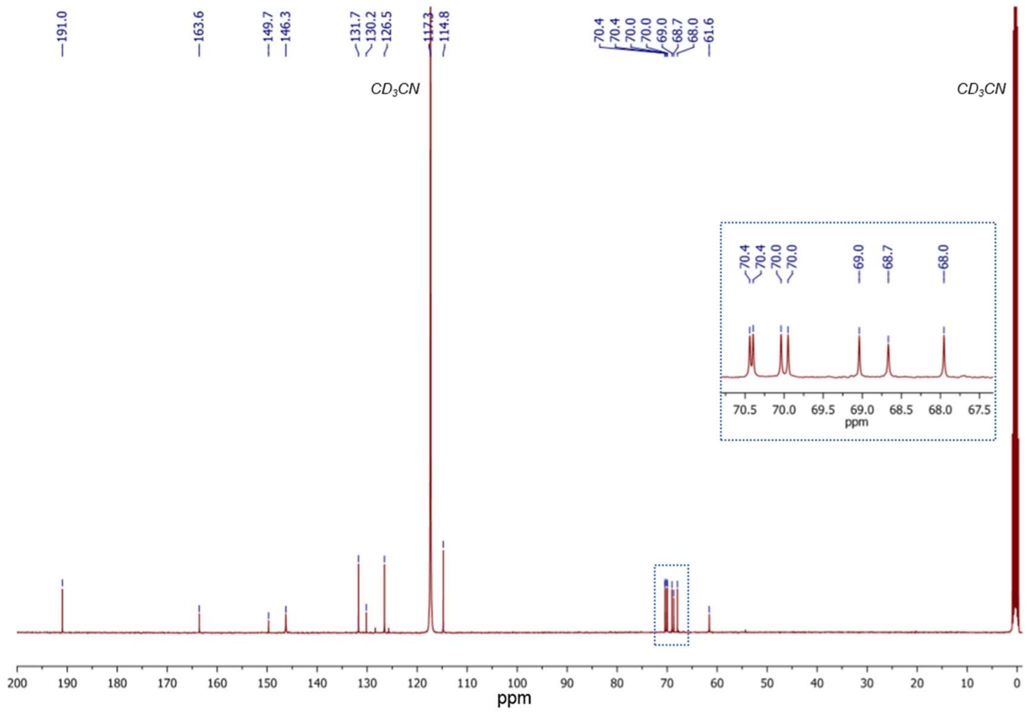

Supplementary Figure 4. ^{13}C NMR spectrum (100 MHz, CD_3CN) of **2**$[PF_6]_2$

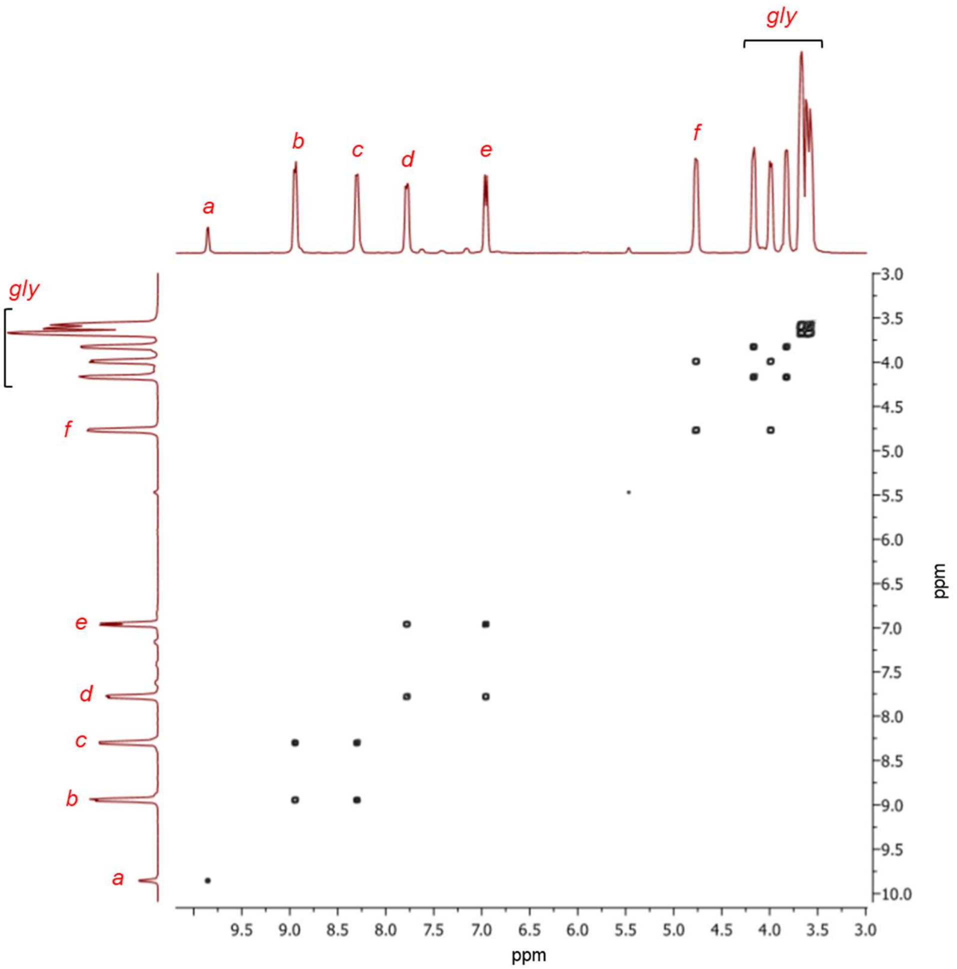

Supplementary Figure 5. ^{1}H-^{1}H COSY NMR spectrum (400 MHz, CD_3CN) of **2**$[PF_6]_2$

Guest molecule 1^{4+}

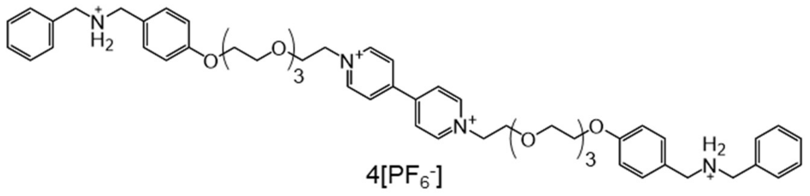

Supplementary Figure 6. Chemical structure of $\mathbf{1}^{4+}$

A solution of **S2**$[PF_6]_2$ (4.7 g, 4.7 mmol) and benzylamine (1.1 g, 9.8 mmol), in CH_2Cl_2 (22 mL), was stirred at room temperature under air for 1h, followed by the addition of sodium triacetoxyborohydride (3.2 g, 14.8 mmol) as a solid. The reaction was stirred at room temperature for 24 h and quenched with an aqueous solution of $NaHCO_3$ (5 wt.%, 100 mL). All organic components were extracted with CH_2Cl_2 (3 × 30 mL), the organic layers were combined and washed with water (30 mL). Then, HCl (1 M, 14 mL) was added and the mixture was stirred at room temperature for 30 min, this produced a brown oily layer between the upper (aqueous) and the bottom (organic) phases. To the obtained brown layer, an aqueous saturated solution of NH_4PF_6 (7 mL) was added and stirred at room temperature to cause the segregation of brown oil. This brown oil was separated from the colorless solution by decantation, washed with CH_2Cl_2 (2 × 30 mL), and then dissolved in CH_3NO_2 (30 mL). The resulting solution was washed with water (2 × 30 mL) and dried under a vacuum. Column chromatography of the crude mixture (SiO_2, CH_3CN/CH_3OH/10 wt.% NH_4Cl (aq) (3:1:1), R_f = 0.37) afforded the tetracationic product as a yellow oil, which was stirred with saturated aqueous NH_4PF_6 (7 mL) at room temperature for 1 h to ensure anion (PF_6^-) homogeneity, and then the product was extracted with CH_3NO_2 (2 × 20 mL). The combined organic layers were dried over Na_2SO_4 and evaporated under reduced pressure to yield $\mathbf{1}^{4+}$ as an orange oil (3.8 g, 2.6 mmol, 55% yield). ^1H NMR (400 MHz, CD_3CN) δ (ppm) = 8.95 (d, J = 6.9 Hz, 4H), 8.37 (d, J = 6.8 Hz, 4H), 7.49 (br, 10H), 7.38 (d, J = 8.7 Hz, 4H), 6.94 (d, J = 8.7 Hz, 4H), 4.78 (t, J = 4.6 Hz, 4H), 4.23 (s, 4H), 4.19 (s, 4H), 4.11 (t, J = 4.4 Hz, 4H), 4.00 (t, J = 4.8 Hz, 4H), 3.80 (t, J =4.4 Hz, 4H), 3.68 – 3.56 (m, 16H). ^{13}C NMR (100 MHz, CD_3CN) δ (ppm) = 159.8, 150.0, 146.2, 131.9, 130.4, 130.2, 129.8, 129.1, 126.7, 122.4, 114.8, 70.3, 70.3, 70.1, 69.9, 69.2, 68.6, 67.6, 61.6, 51.2, 50.9. ESI-HRMS: m/z calculated for $[\mathbf{1} + (PF_6)_2]^{2+}$ $C_{54}H_{70}F_{12}N_4O_8P_2$, 596.2233; found, 596.2236; relative error 0.5 ppm

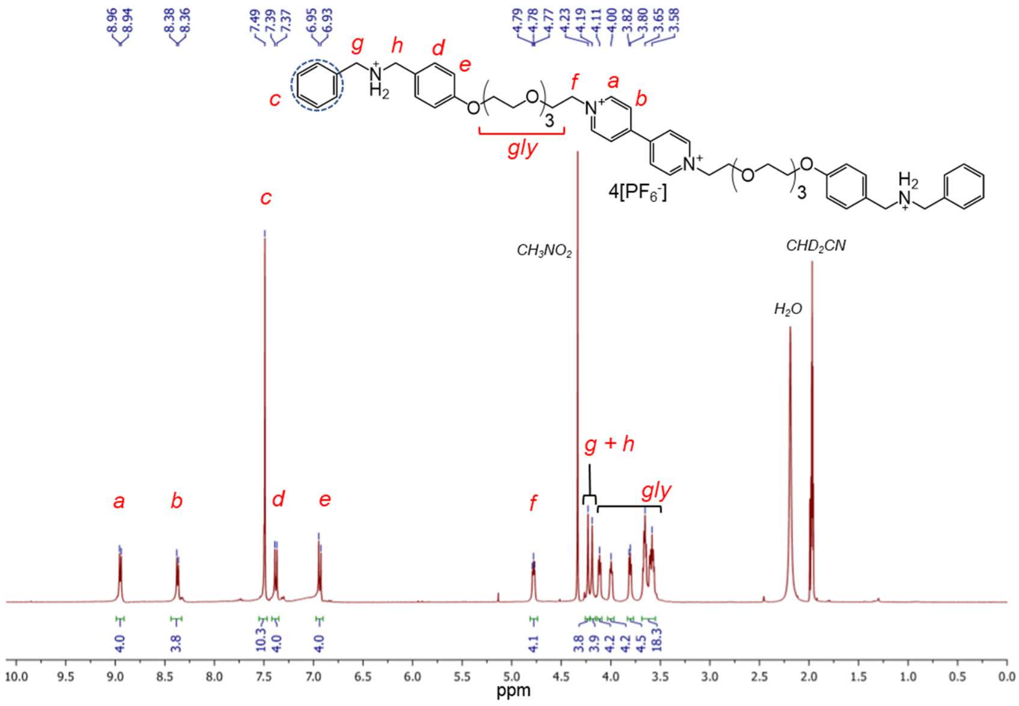

Supplementary Figure 7. ^{1}H NMR spectrum (400 MHz, CD_3CN) of **1**$^{4+}$

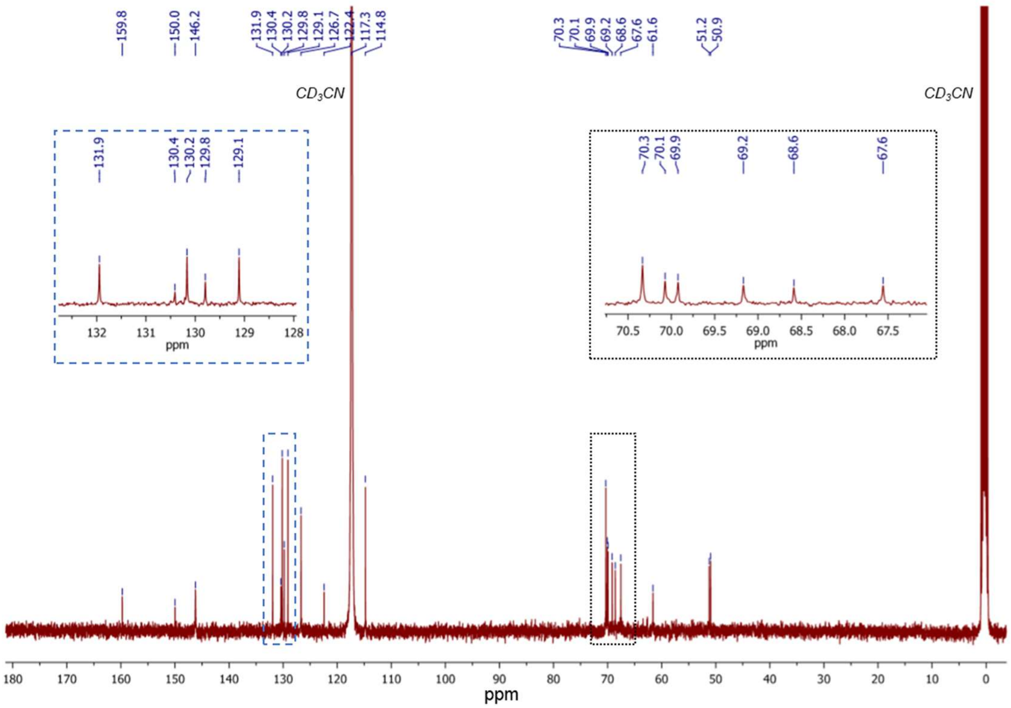

Supplementary Figure 8. ^{13}C NMR spectrum (100 MHz, CD_3CN) of **1**$^{4+}$

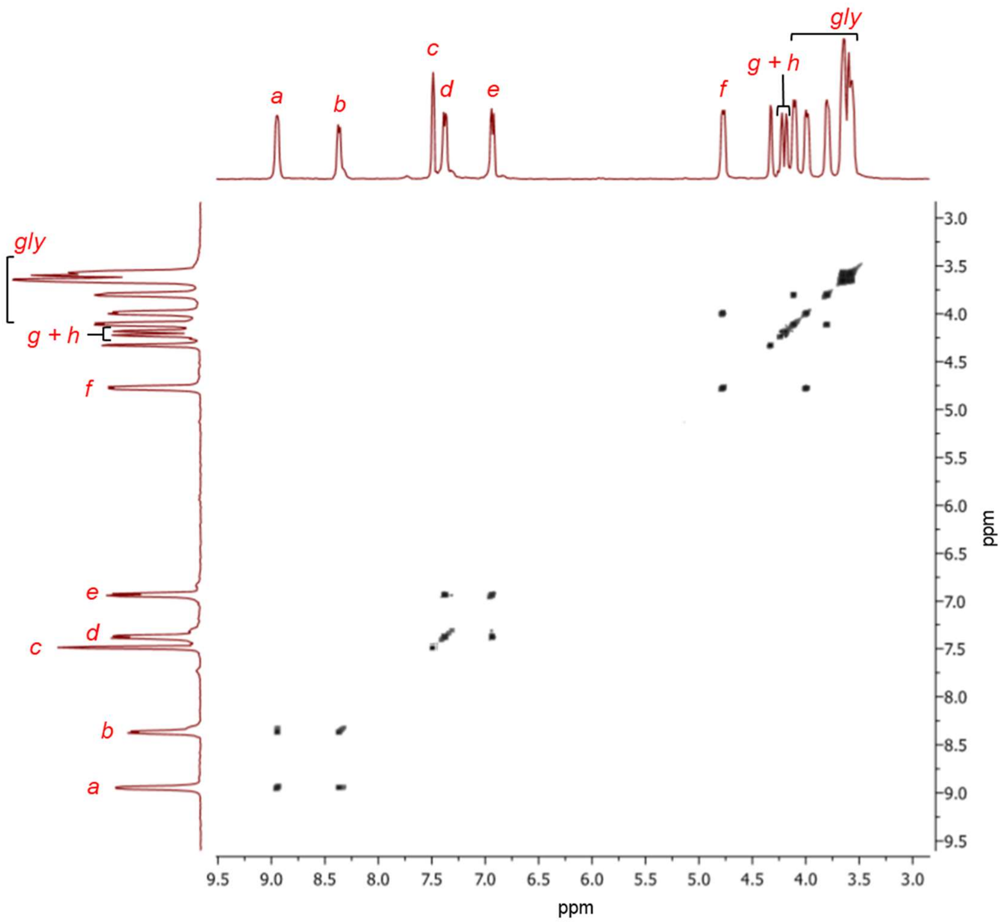

Supplementary Figure 9. ^1H-^1H COSY NMR spectrum (400 MHz, CD_3CN) of $\mathbf{1}^{4+}$

Prospective dumbbell molecules

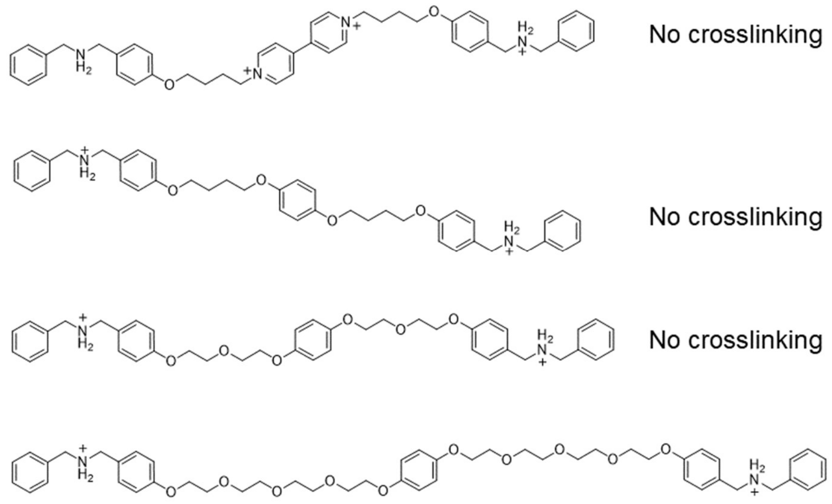

Supplementary Figure 10. Dumbbell molecules discarded because of their poor crosslinking performance or solvent incompatibility.

Pseudorotaxane Formation

We investigated the self-assembly of $\mathbf{1}^{4+}$with the crown ethers **DN38C10** and **DB24C8** to produce a set of pseudorotaxanes (**PR**s): [2]pseudorotaxane [$\mathbf{1}^{4+}$ ⊂ **DN38C10**] ([2]**PR**), [3]pseudorotaxane [$\mathbf{1}^{4+}$ ⊂ (**DB24C8**)$_2$] ([3]**PR**), and [4]pseudorotaxane [**DN38C10**⊃ $\mathbf{1}^{4+}$ ⊂ **DB24C8**)$_2$] ([4]**PR**). Compound $\mathbf{1}^{4+}$ (5.0 mM) was separately mixed with the corresponding ring(s): **DN38C10** (5.0 mM), **DB24C8** (10.0 mM), and **DN38C10** (5.0 mM)/**DB24C8** (10.0 mM), in a mixture of acetonitrile and chloroform (1/1, v/v). The resulting solutions were analyzed by 1-D (^{1}H) and 2-D (^{1}H-^{1}H NOESY and EXSY) NMR spectroscopy, UV-vis spectroscopy, and ESI-HRMS.

Characterization of [2]PR [$1^{4+} \subset$ DN38C10]

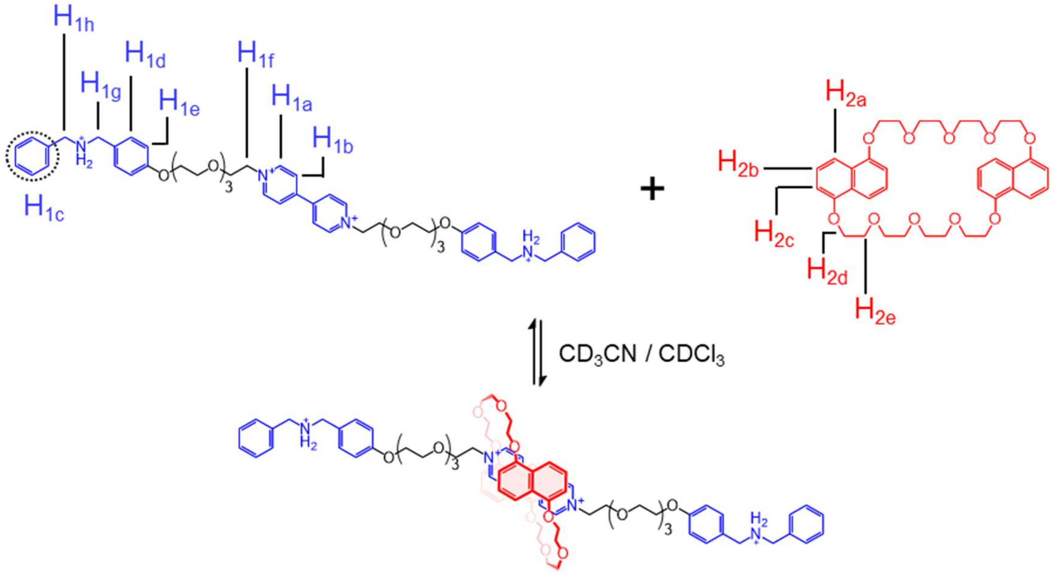

Supplementary Scheme 2. [2]**PR** formation with **1**$^{4+}$ and **DN38C10**

The addition of host (**DN38C10**) to a solution of guest $\mathbf{1^{4+}}$ in $CD_3CN/CDCl_3$ (1:1, v/v, 5.0 mM) resulted in an immediate change of color from yellow to red, which suggested spontaneous intercomponent self-assembly (Supplementary Figure 11 (photos)). In the corresponding UV-vis spectrum, we confirmed the presence of a new band centered at 486 nm (Supplementary Figure 11 (spectra: a (host), c (guest), and d (mixture)), which is attributed to a charge-transfer from the electron-rich naphthyl moieties of the **DN38C10** to the 4,4′-bipyridinium (BIPY) core of **1**$^{4+}$.NMR spectroscopy further confirmed the formation of [2]**PR** [**1**$^{4+} \subset$ **DN38C10**]$^{4+}$. In the collected ^{1}H NMR spectrum (Supplementary Figure 12), we observed only one set of resonances, implying that the assembly/disassembly process is fast compared to the NMR time-scale. This correlates well with other host-guest systems composed of **DN38C10** and a viologen.[8] Moreover, we detected that the bipyridinium protons (H_{1a} and H_{1b}) shifted upfield compared with a pure sample of **1**[PF_6]$_4$, *e.g.* H_{1b} displayed a $\Delta\delta = 0.55$ ppm, which can be assigned to host–guest π-stacking. The remaining protons, for instance, those of the dibenzylammonium (DBA) stations, were barely affected. These observations corroborate that the **DN38C10** ring sits on the central portion of **1**$^{4+}$ yielding a [2]**PR** structure. In fact, in a ^{1}H-^{1}H NOESY NMR spectrum (Supplementary Figure 13) we observed correlations between protons H_{1a} and H_{2d}, and H_{1f} and H_{2d}, which indicates that the BIPY and **DN38C10** components are spatially close. Finally, ESI-HRMS showed the molecular ion [**1** + **DN38C10** + (2 × PF_6)]$^{2+}$ at m/z = 914.3697 for, ($C_{90}H_{114}F_{12}N_4O_{18}P_2$ calc. 914.3700; relative error -0.3 ppm).

The association constant (K_a) of [2]**PR**, in acetonitrile and chloroform (1:1, v/v) at 25 ºC, was

determined by the titration of **1^{4+}** with **DN38C10** (Supplementary Figure 14), monitored by ^{1}H NMR spectroscopy.

The resulting isotherms were fitted by a nonlinear least-squares method using a 1:1 global fitting model (Nelder-Mead method) in the BindFit platform.[9] ΔG was determined by applying Equation (1). The obtained values are summarized in Supplementary Table 1.

$\boldsymbol{\Delta G = -RT\ln K_a}$ ... Equation (1)

Supplementary Table 1. Thermodynamic parameters for [2]**PR** ($CD_3CN/CDCl_3$ (1:1, v/v) at 298 K)

K_a (M^{-1})	ΔG (kJ mol^{-1})
$(1.11 \pm 0.17) \times 10^2$	-11.7 ± 0.4

Standard deviation was calculated based on the results from two protons (H_{1a} and H_{1f})

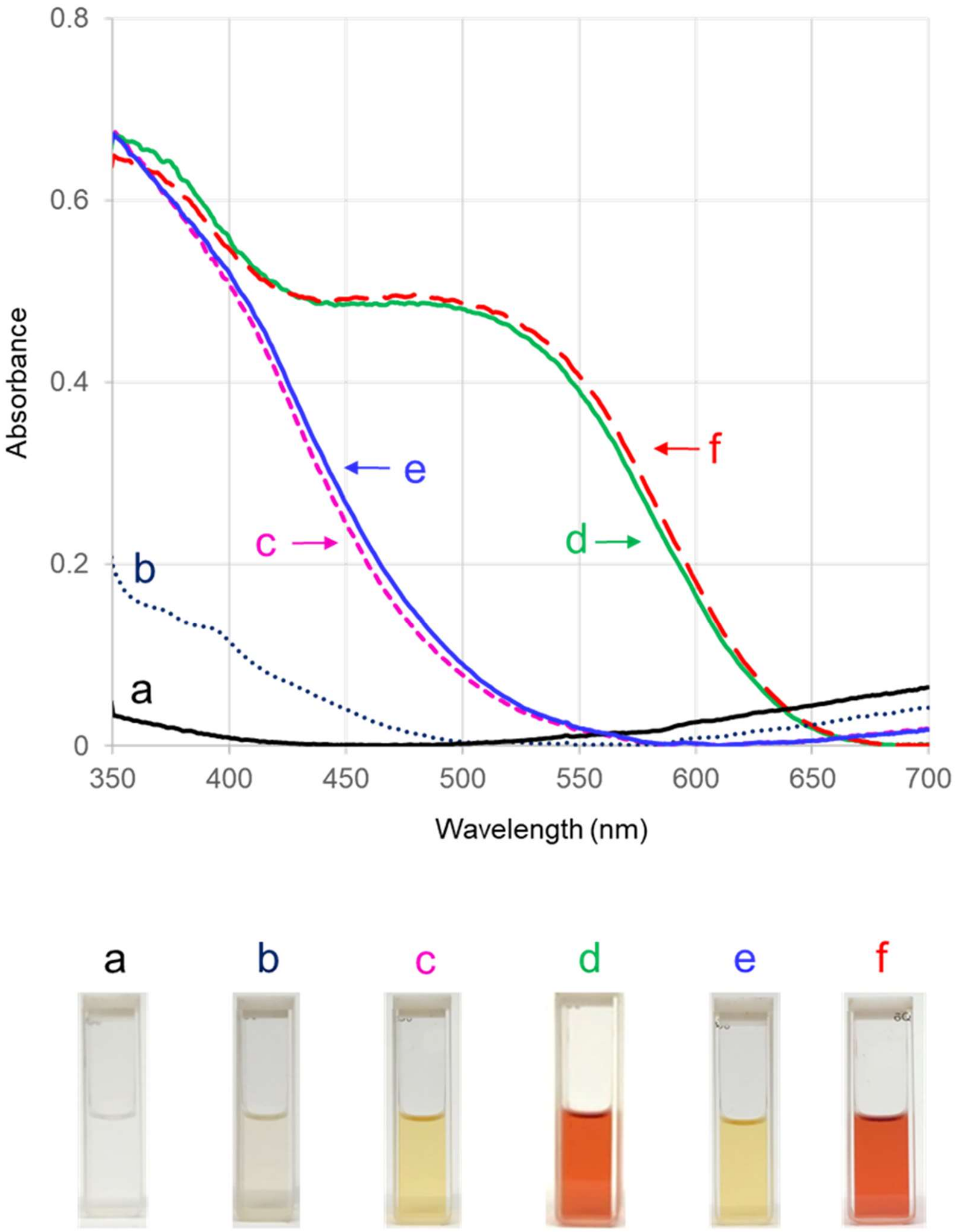

Supplementary Figure 11. UV-vis spectrum and solution appearance of a) **DN38C10**, b) **DB24C8**, c) **1**[PF_6]$_4$, d) 1:1 mixture of **1**[PF_6]$_4$ and **DN38C10**, e) 1:2 mixture of **1**[PF_6]$_4$ and **DB24C8**, and f) 1:1:2 mixture of **1**[PF_6]$_4$, **DN38C10** and **DB24C8**; samples were prepared in $CH_3CN/CHCl_3$ (1:1, v/v) at 10 mM (b) and at 5 mM (others).

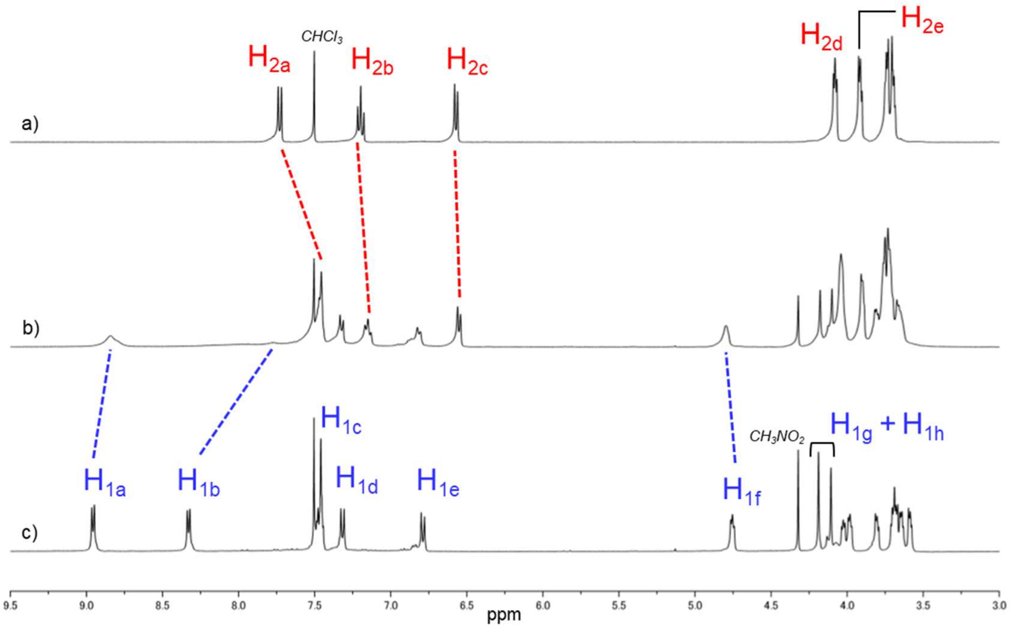

Supplementary Figure 12. Partial ^{1}H NMR spectra (400 MHz, $CD_3CN/CDCl_3$ = 1:1, v/v, 5.0 mM). a) **DN38C10**, c) $\mathbf{1}^{4+,}$ and b) 1:1 mixture of $\mathbf{1}^{4+}$ and **DN38C10**; dash lines indicate peak shifts upon mixing the compounds.

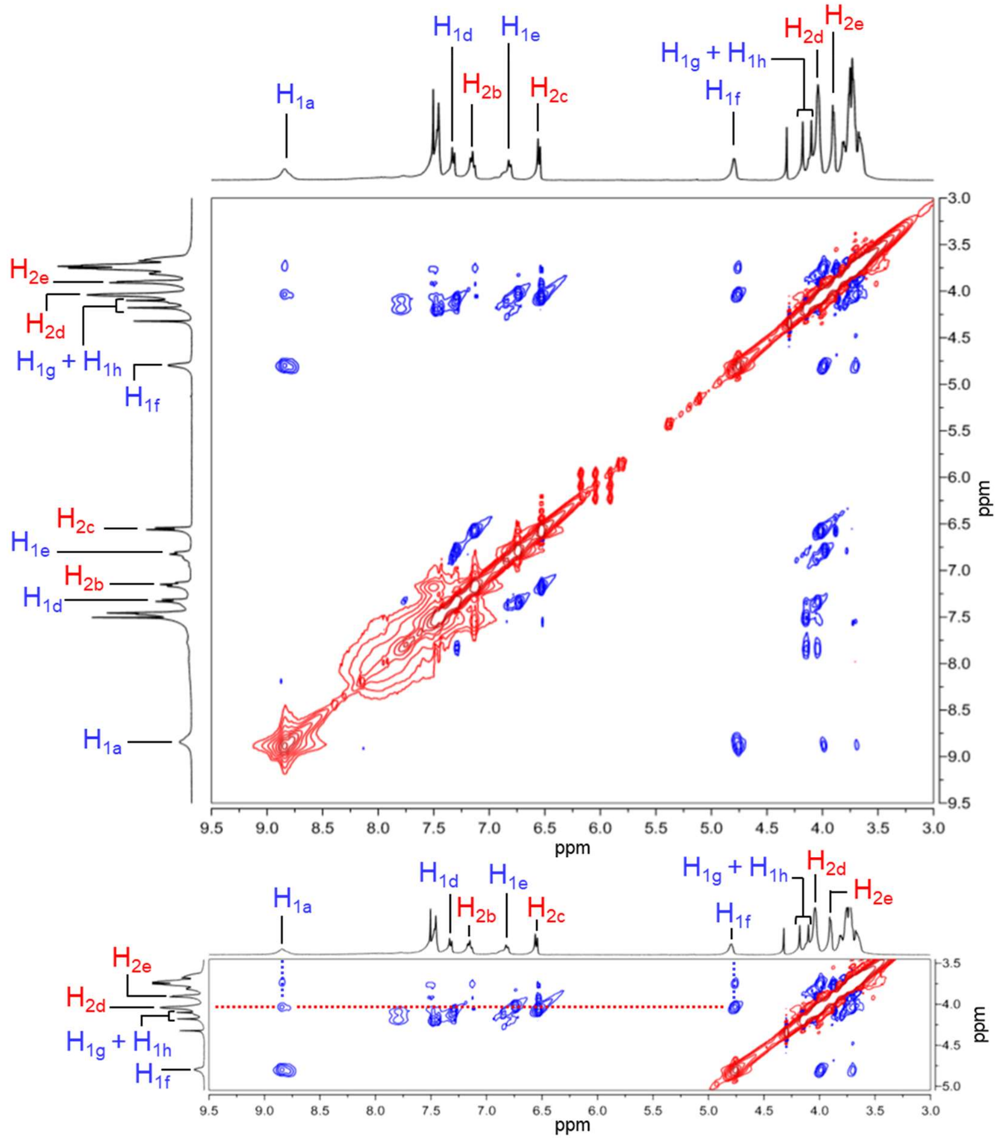

Supplementary Figure 13. ^{1}H-^{1}H NOESY NMR spectra (400 MHz, $CD_3CN/CDCl_3$ = 1:1, v/v), 5.0 mM) for [2]**PR** [$\mathbf{1^{4+}} \subset$ **DN38C10**]: Full and zoom-in views; dash lines indicate correlations.

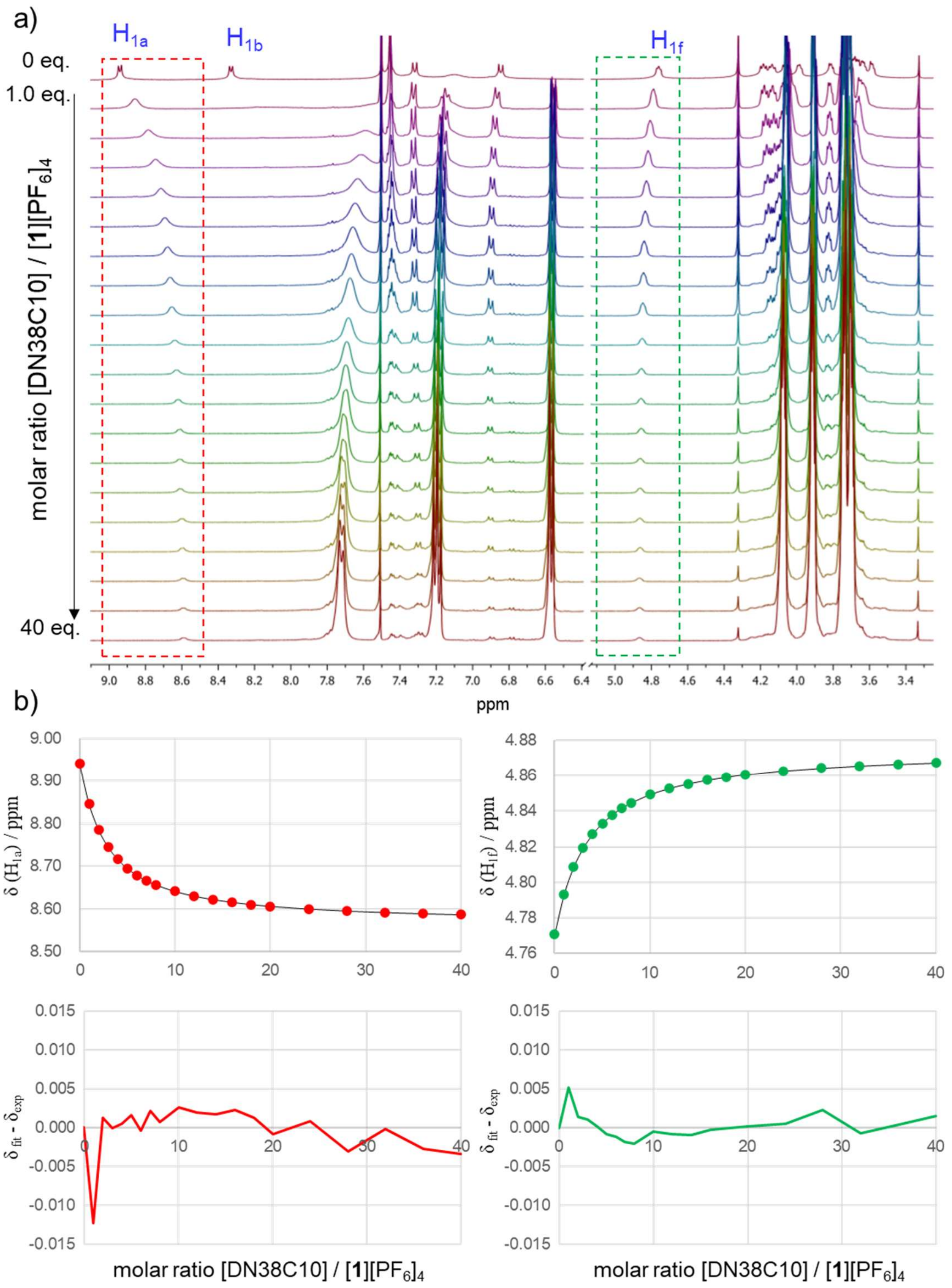

Supplementary Figure 14. a) ^{1}H NMR spectra (400MHz, $CD_3CN/CDCl_3$ = 1:1, v/v, 298 K) for titration of **1**$^{4+}$ (2.0 mM) with **DN38C10** (50.0 mM). b) Titration isotherms for H_{1a} (left) and H_{1f} (right) protons of **1**$^{4+}$; experimental data (dots), fitted data (black line).

Characterization of [3]PR [$1^{4+} \subset (\mathbf{DB24C8})_2$]

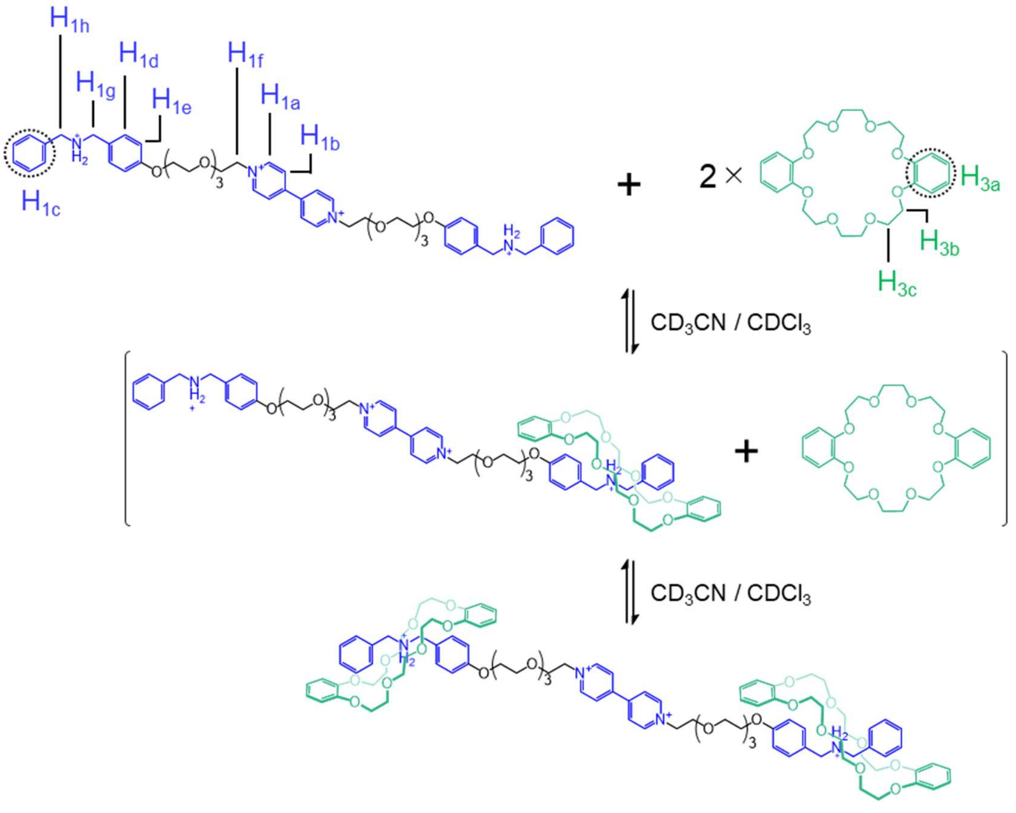

Supplementary Scheme 3. [3]**PR** formation with **1**$^{4+}$ and two equivalents of **DB24C8**

Two equivalents of the host (**DB24C8**) were added into a solution of **1**$^{4+}$ in $CD_3CN/CDCl_3$ (1/1, v/v, 5.0 mM) showing no significant changes in solution color and UV-vis spectrum (Supplementary Figure 11), which suggests that, as expected, there was no charge-transfer complex forming. NMR spectroscopy confirmed the formation of [3]**PR** [**1**$^{4+}$ ⊂ (**DB24C8**)$_2$] (Supplementary Scheme 3). In a ^{1}H NMR spectrum (Supplementary Figure 15), two sets of peaks corresponding to the complexed and the uncomplexed species were observed, indicating that the assembly/disassembly process is slower than the NMR time-scale. Peak splitting was observed in DBA moieties (H_{1c}, H_{1d}, H_{1e}, H_{1g}, and H_{1h}) and **DB24C8** (H_{3a} and H_{3c}) protons. The other protons, for instance, those of bipyridinium, were barely affected. Additionally, a ^{1}H-^{1}H EXSY NMR spectrum (Supplementary Figure 16) detected chemical exchange between DBA (H_{1c}, H_{1d}, H_{1e}, H_{1g}, and H_{1h}) and **DB24C8** (H_{3a} and H_{3c}) protons, supporting the slow equilibrium involving DBA and **DB24C8**. Furthermore, in a ^{1}H-^{1}H NOESY NMR spectrum (Supplementary Figure 17), we observed correlations of H_{3c} with H_{1c}, H_{1d}, H_{1e}, H_{1g}, and H_{1h}. These results indicate that the DBA moieties of both ends of **1**$^{4+}$ are occupied by **DB24C8** rings. Finally,

ESI-HRMS spectrometry showed the molecular ion [**1** + (2 × **DB24C8**) + (PF_6)]$^{3+}$ at *m*/*z* = 648.3021 for, ($C_{102}H_{134}F_6N_4O_{24}P$ calc. 648.3016; relative error 0.8 ppm).

The global association constant of the [3]**PR** in acetonitrile and chloroform (1:1, v/v) at 25 ºC, was determined by the Equation (2) using the integration of the uncomplexed proton (H_{3a}(uc)) and complexed proton (H_{3a}(c)) on **DB24C8** in ^{1}H NMR spectrum (Supplementary Figure 15). The global association constant was determined to ((3.2 ± 0.2) × 10^4 M^{-2}) from three independent experiments.

$$K_a = \frac{[\mathbf{1}+(\boldsymbol{DB24C8})_2]}{([\mathbf{1}])\cdot([\boldsymbol{DB24C8}])^2} \quad \dots \text{ Equation (2)}$$

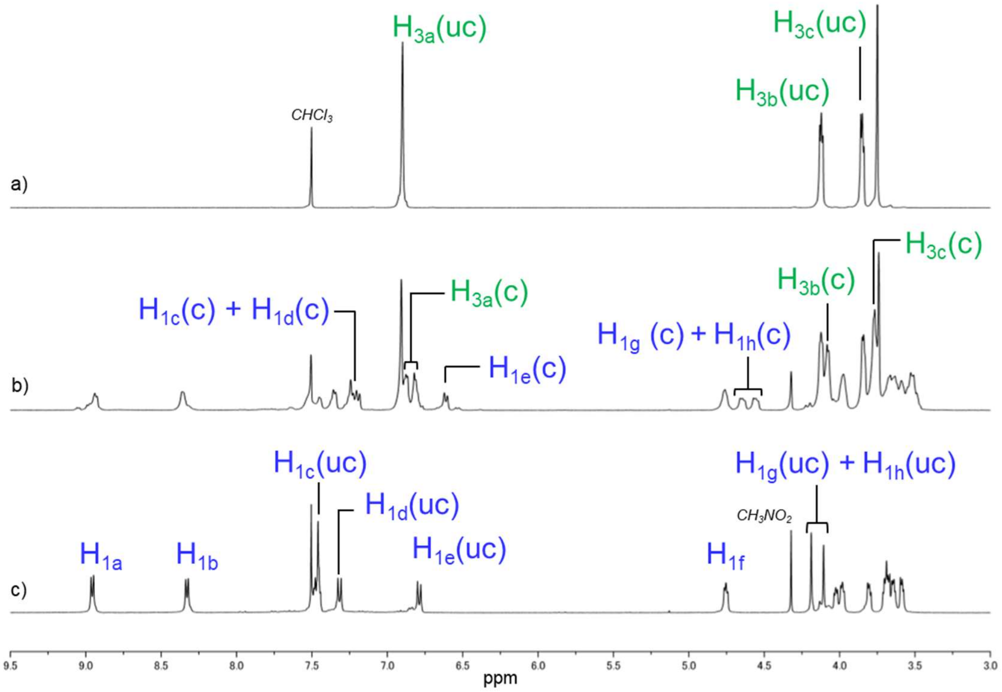

Supplementary Figure 15. Partial ^{1}H NMR spectra (400 MHz, $CD_3CN/CDCl_3$ = 1:1 (v/v), 5.0 mM). a) **DB24C8**, c) $\mathbf{1}^{4+}$, and b) 1:2 mixture of $\mathbf{1}^{4+}$ and **DB24C8**; “c” and “uc” in the parentheses denote peaks arising from protons on complexed and uncomplexed components, respectively.

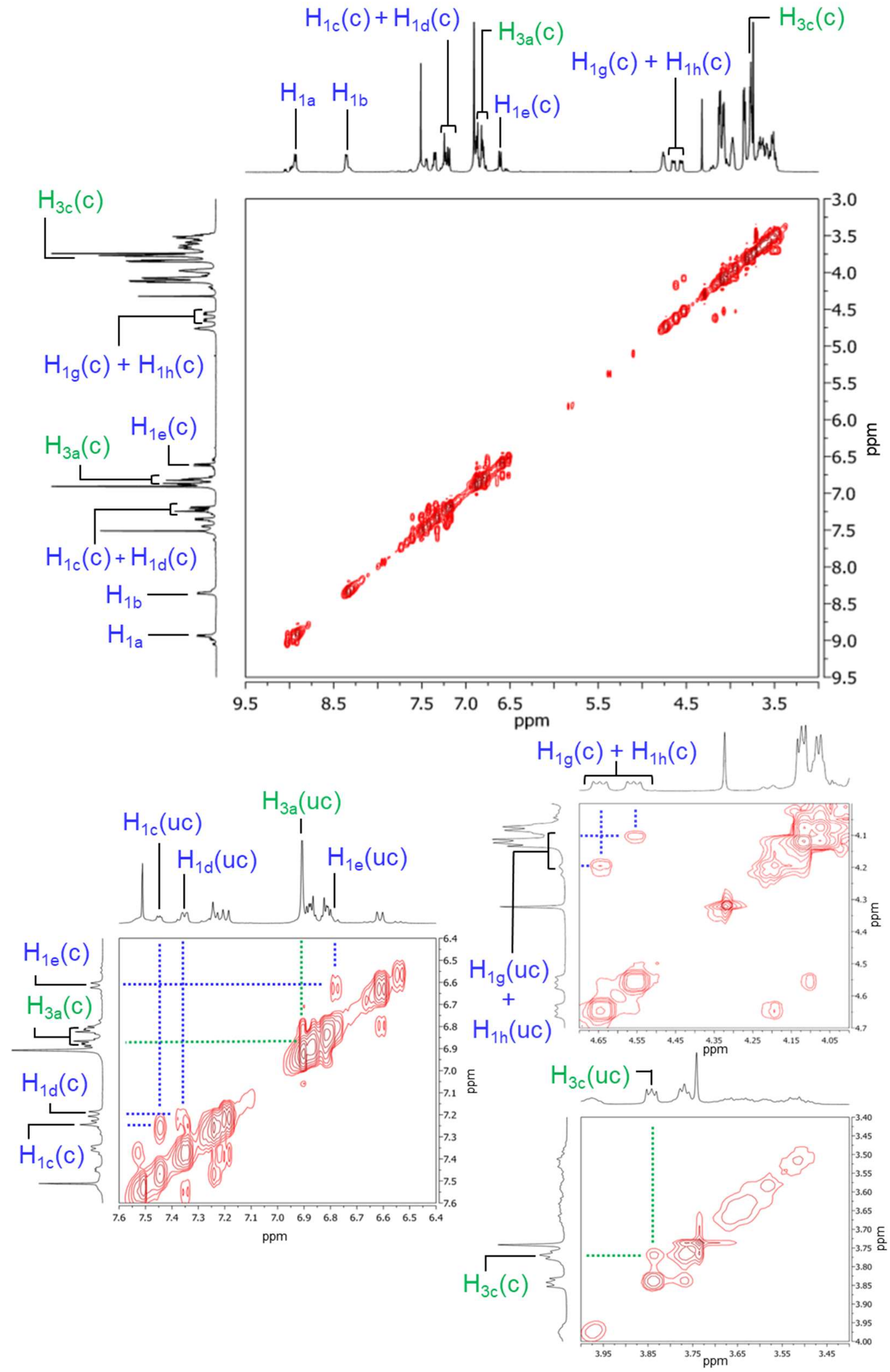

Supplementary Figure 16. ^{1}H-^{1}H EXSY NMR spectrum (400 MHz, $CD_3CN/CDCl_3$ = 1:1, v/v, 5.0 mM) for [3]**PR**:

[$1^{4+} \subset$ (**DB24C8**)$_2$]. Full and zoom-in views; dash lines indicate correlations.

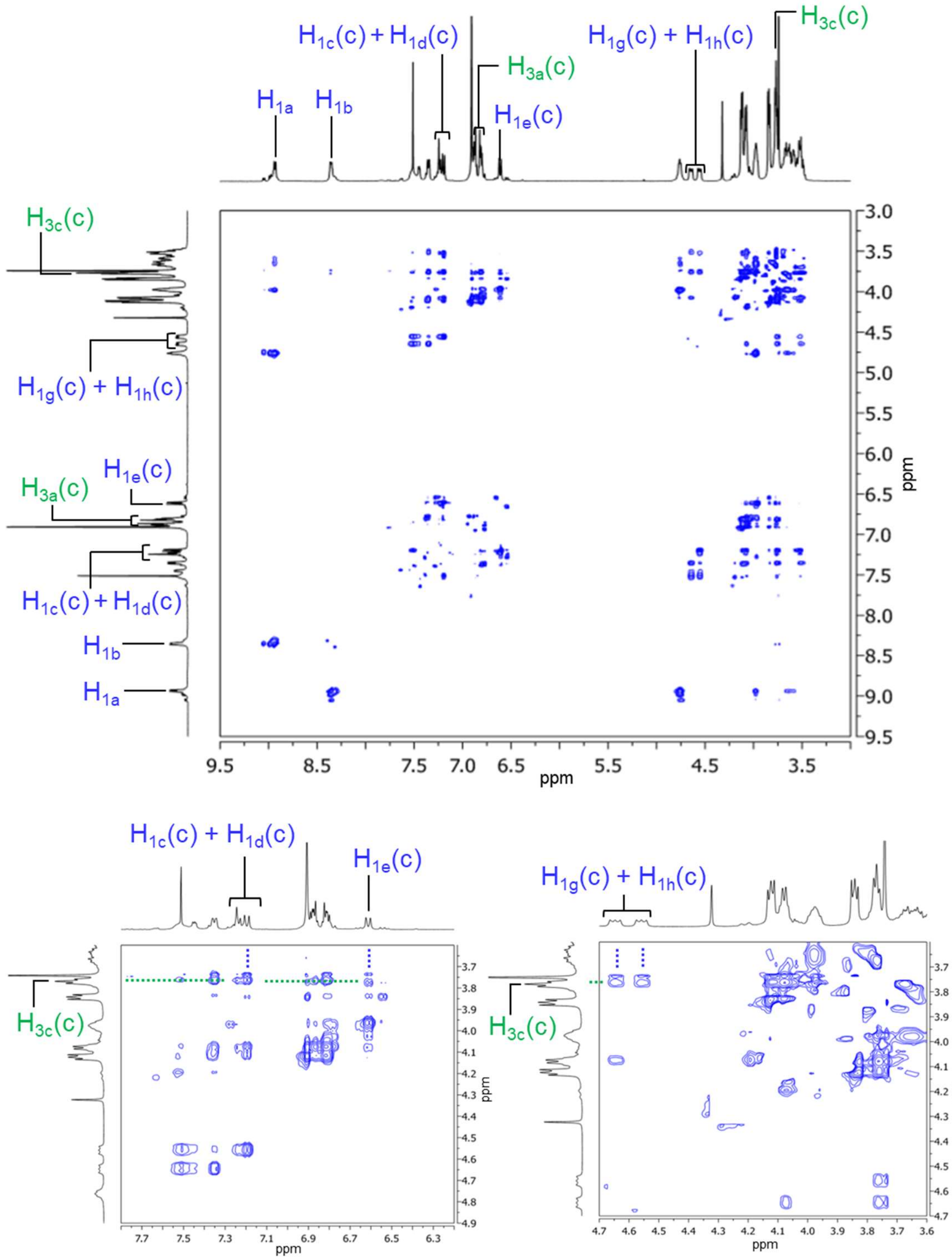

Supplementary Figure 17. ^{1}H-^{1}H NOESY NMR spectrum (400 MHz, $CD_3CN/CDCl_3$ = 1:1 (v/v), 5.0 mM) for [3]**PR**: [$1^{4+} \subset$ (**DB24C8**)$_2$]. Full and zoom-in views; dash lines indicate correlations.

Characterization of [4]PR [DN38C10 ⊃ 1^{4+} ⊂ (DB24C8)$_2$]

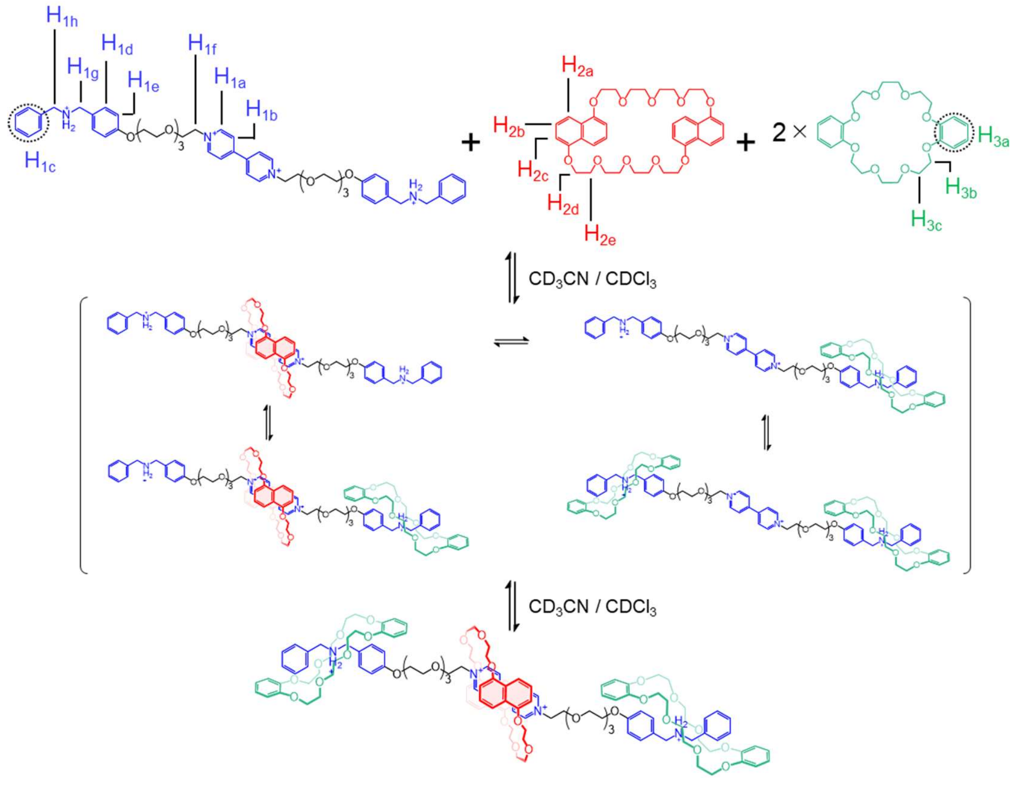

Supplementary Scheme 4. [4]**PR** formation with $\mathbf{1}^{4+}$, one equivalent of **DN38C10**, and two equivalents of **DB24C8**

The addition of hosts (one equivalent of **DN38C10** and two equivalents of **DB24C8**) to a solution of guest $\mathbf{1}^{4+}$ in $CD_3CN/CDCl_3$ (1:1, v/v, 5.0 mM) immediately changed solution color from yellow to red, which suggested spontaneous intercomponent self-assembly (Supplementary Figure 11). In the corresponding UV-vis spectrum, we observed the band overlapped with [2]**PR** (Supplementary Figure 11) suggesting the formation of the same charge-transfer structure as [2]**PR**, where **DN38C10** sits on the bipyridinum core of $\mathbf{1}^{4+}$. According to NMR spectroscopy, the formation of [4]**PR** [**DN38C10** ⊃ $\mathbf{1}^{4+}$ ⊂ (**DB24C8**)$_2$] (Supplementary Scheme 4) was confirmed. In the collected ^{1}H NMR spectrum (Supplementary Figure 18), we observed two sets of peaks for BIPY core (H_{1a}) and DBA moieties (H_{1c}, H_{1d}, H_{1g}, and H_{1f}) of $\mathbf{1}^{4+}$, and **DB24C8** (H_{3a} and H_{3c}), respectively, corresponding to the complexed and the uncomplexed species due to the slow assembly/disassembly process. This result was supported by the observation of chemical exchanges for BIPY core (H_{1a}) and DBA (H_{1c}, H_{1d}, H_{1g},

and H_{1f}) moieties of $\mathbf{1}^{4+}$, and **DB24C8** (H_{3a}) in ^{1}H-^{1}H EXSY NMR (Supplementary Figure 19). Furthermore, in ^{1}H-^{1}H NOESY NMR (Supplementary Figure 20) we confirmed the correlations of H_{3c} with H_{1c}, H_{1d}, H_{1e}, H_{1g}, and H_{1h}, respectively, suggesting that **DB24C8** sit on the DBA moieties of $\mathbf{1}^{4+}$, as well as [3]**PR**. The assembly/disassembly motion of **DN38C10** and BIPY core of $\mathbf{1}^{4+}$ became slower in [4]**PR** than that in [2]**PR**, probably because the speed was limited by the slow assembly/disassembly of **DB24C8** and DBA moieties of both ends of $\mathbf{1}^{4+}$. Finally, ESI-HRMS showed the molecular ion [**1** + **DN38C10** + (**DB24C8**)$_2$]$^{4+}$ at m/z = 609.0589 for, ($C_{138}H_{178}N_4O_{34}$ calc. 609.0584; relative error 0.8 ppm). Summarizing the results from UV-vis spectroscopy, NMR spectroscopy, and ESI-HRMS, $\mathbf{1}^{4+}$ was proved to have the capability to form [4]**PR**, where its BIPY core is occupied with one **DN38C10** and the two DBA moieties were bound to two **DB24C8**.

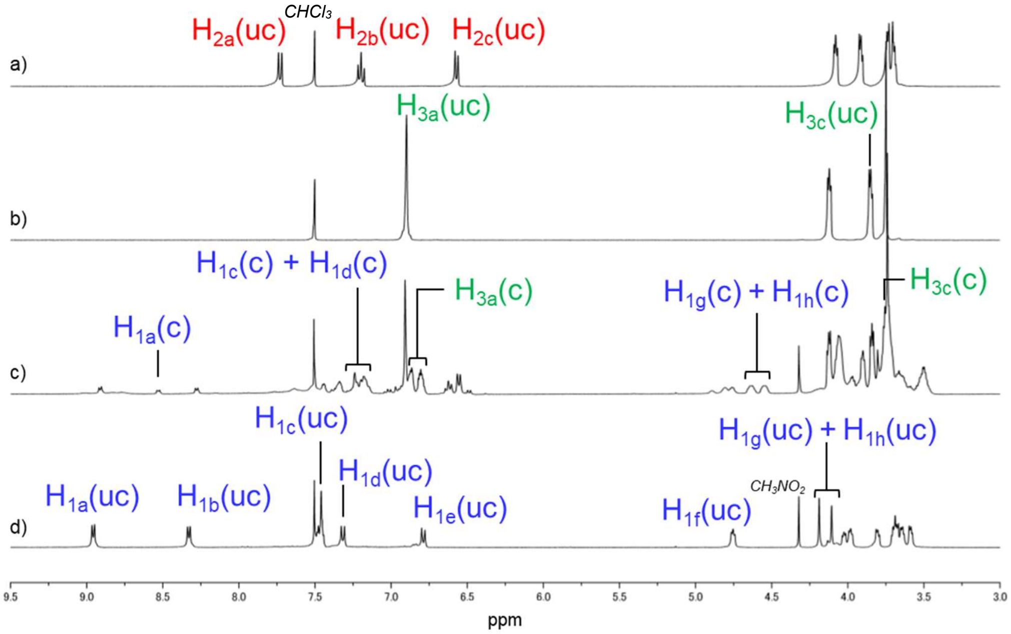

Supplementary Figure 18. Partial ^{1}H NMR spectra (400 MHz, $CD_3CN/CDCl_3$ = 1:1 (v/v), 5.0 mM). a) **DN38C10**, b) **DB24C8**, c) 1:1:2 mixture of $\mathbf{1}^{4+}$, **DN38C10**, and **DB24C8**, and d) $\mathbf{1}^{4+}$; “c” and “uc” in the parentheses denote peaks arising from protons on complexed and uncomplexed components, respectively.

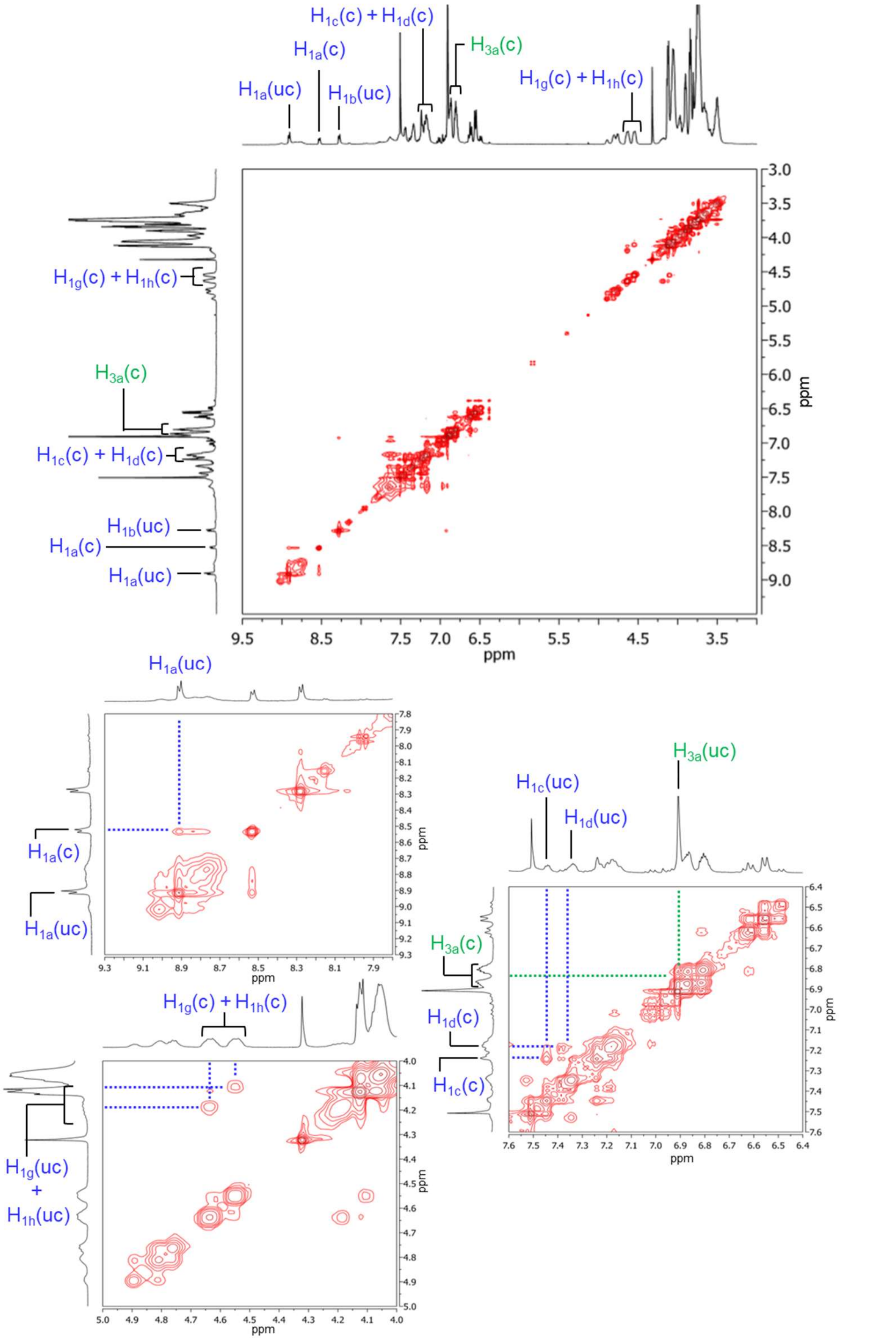

Supplementary Figure 19. ^{1}H-^{1}H EXSY NMR spectrum (400 MHz, $CD_3CN/CDCl_3$ = 1:1, v/v, 5.0 mM) for [4]**PR**: [**DN38C10** ⊃ **1**$^{4+}$ ⊂ **(DB24C8)$_2$**]. Full and zoom-in views; "c" and "uc" in the parentheses denote peaks arising from protons on complexed and uncomplexed components, respectively. Dash lines indicate correlations.

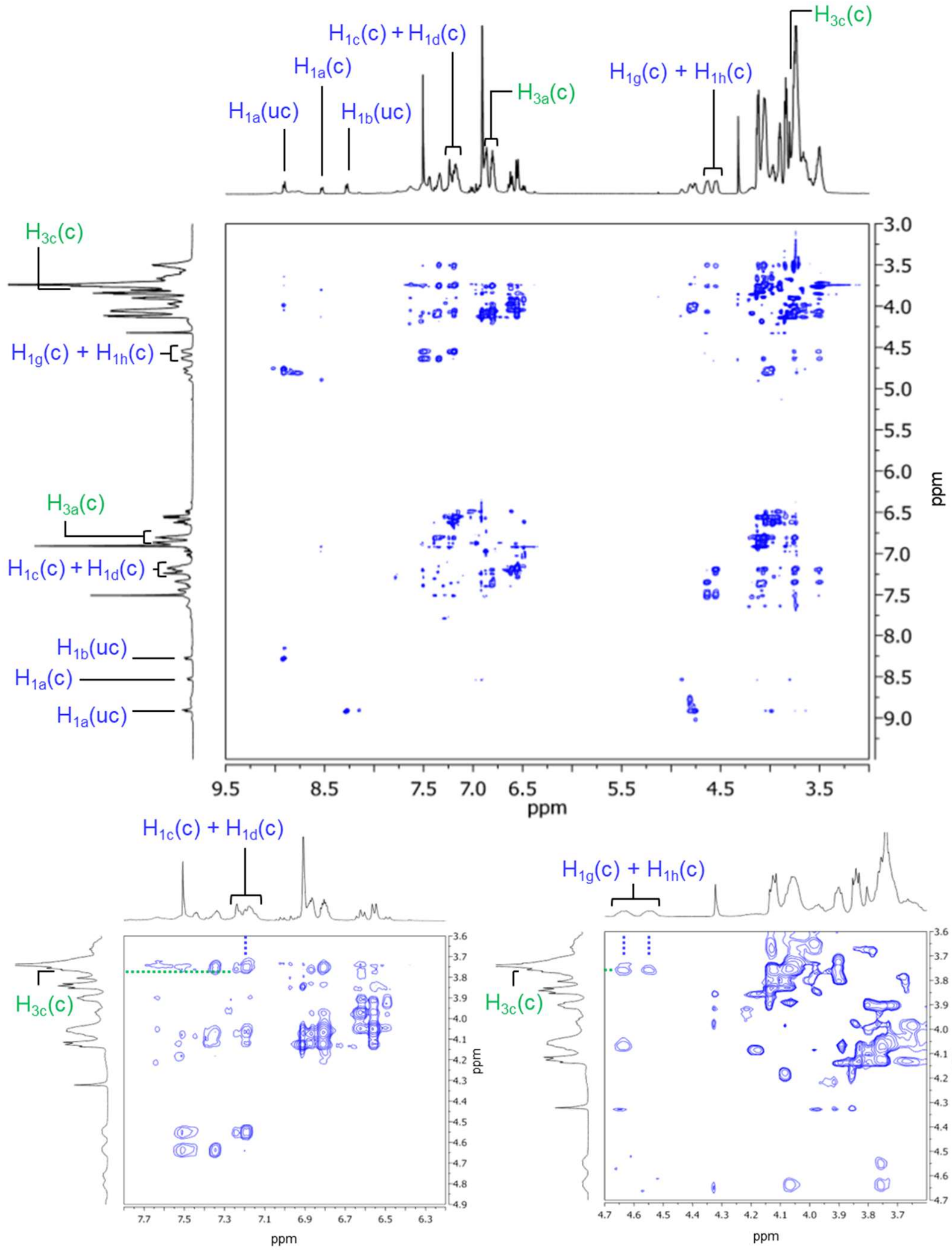

Supplementary Figure 20. ^{1}H-^{1}H NOESY NMR spectrum (400 MHz, $CD_3CN/CDCl_3$ = 1:1 (v/v), 5.0 mM) for [4]**PR**: [**DN38C10** ⊃ $\mathbf{1}^{4+}$ ⊂ **(DB24C8)**$_2$]. Full and zoom-in views; “c” and “uc” in the parentheses denote peaks arising from protons on complexed and uncomplexed components, respectively. Dash lines indicate correlations.

<u>Metastable [3]rotaxane ([3]MSR)</u>

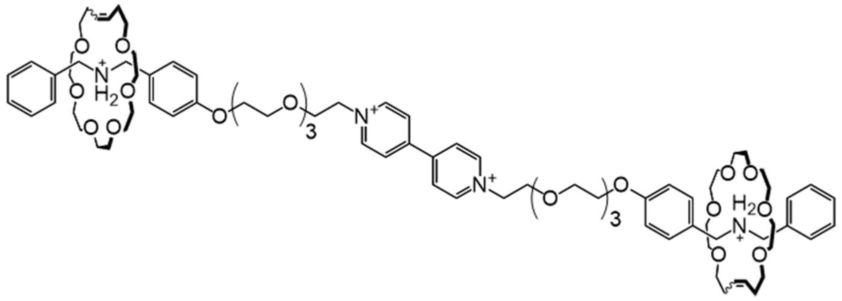

Supplementary Figure 21. Chemical structure of [3]**MSR**

Compound $\mathbf{1}^{4+}$ (0.75 g, 0.50 mmol) and pentaethylene glycol di-but-4-enyl-ether (0.62 g,1.80 mmol) were dissolved in a mixture of CH_3CN and $CHCl_3$ (15 mL, 1:1, v/v), and stirred at room temperature overnight. Both solvents were evaporated under vacuum keeping the temperature below 30 ºC. A solution of the residual orange oil, prepared in dry CH_2Cl_2 (500 mL), was loaded with Grubbs catalyst 2nd generation (91.0 mg, 0.11 mmol) under the protection of a N_2 atmosphere, and heated at 45 ºC for 60 h. After cooling down to room temperature, the reaction was quenched with ethyl vinyl ether (2 mL), followed by rotary evaporation (maintaining T below 30 ºC). Column chromatography (SiO_2, CH_2Cl_2/CH_3OH = 92:8 (v/v), R_f = 0.33) yielded [3]**MSR** in 28% yield as a dark brown oil. ^{1}H NMR (400 MHz, CD_3CN) δ = 8.98 (d, J = 6.6 Hz, 4H), 8.43 (d, J = 6.5 Hz, 4H), 7.65 (br, 4H), 7.56 – 7.44 (m, 14H), 6.99 (d, J = 8.7 Hz, 4H), 5.62 – 5.57 (m, 4H), 4.80 (t, J = 4.8 Hz, 4H), 4.51 – 4.41 (m, 8H), 4.14 (t, J = 4.4 Hz, 4H), 4.01 (t, J = 4.6 Hz, 4H), 3.81 (t, J = 4.4 Hz, 4H), 3.72 – 3.56 (m, 40H), 3.51 – 3.46 (m, 8H), 3.30 – 3.26 (m, 8H), 3.06 (s, 8H), 2.42 – 2.31 (m, 8H). ^{13}C NMR (100 MHz, CD_3CN) δ = 159.5, 159.5, 150.1, 146.3, 132.3, 132.3, 132.0, 132.0, 130.6, 130.2, 130.2, 130.1, 129.2, 129.2, 128.7, 126.8, 124.4, 114.5, 71.7, 71.5, 71.5, 71.4, 71.0, 70.8, 70.5, 70.4, 70.4, 70.3, 70.3, 70.2, 70.2, 70.1, 69.9, 69.2, 68.6, 67.6, 61.7, 52.1, 51.8, 51.5, 32.8, 29.9, 28.3. ESI-HRMS: m/z calculated for [[3]**MSR** + $(PF_6)_2]^{2+}$ $C_{86}H_{130}F_{12}N_4O_{20}P_2$, 914.4276; found, 914.4279; relative error 0.3 ppm.

Postprocessing of [3]**MSR** allowed us to obtain a less colored material, which was relevant for our following studies. Rotaxane [3]**MSR** (484 mg) was dissolved in CH_3CN (10 mL) and stirred with H_2O_2 aq (30%, 20 mL) at room temperature for 2 h, followed by evaporation of CH_3CN by rotary evaporation. The residue was extracted with CH_2Cl_2 (4 × 5 mL), washed with water (2 × 10 mL), and then dried over Na_2SO_4. After evaporating the organic solvents, [3]**MSR** was isolated an orangish-brown glassy solid (400 mg, 82% yield). The collected ^{1}H NMR spectrum was identical to that described above, and PF_6^- remained as the counterion, which was confirmed by ^{19}F (Supplementary Figure 26) and ^{31}P (Supplementary Figure 27) NMR spectroscopy. This treatment allowed removal of the remaining traces of colorful Ru-containing species.

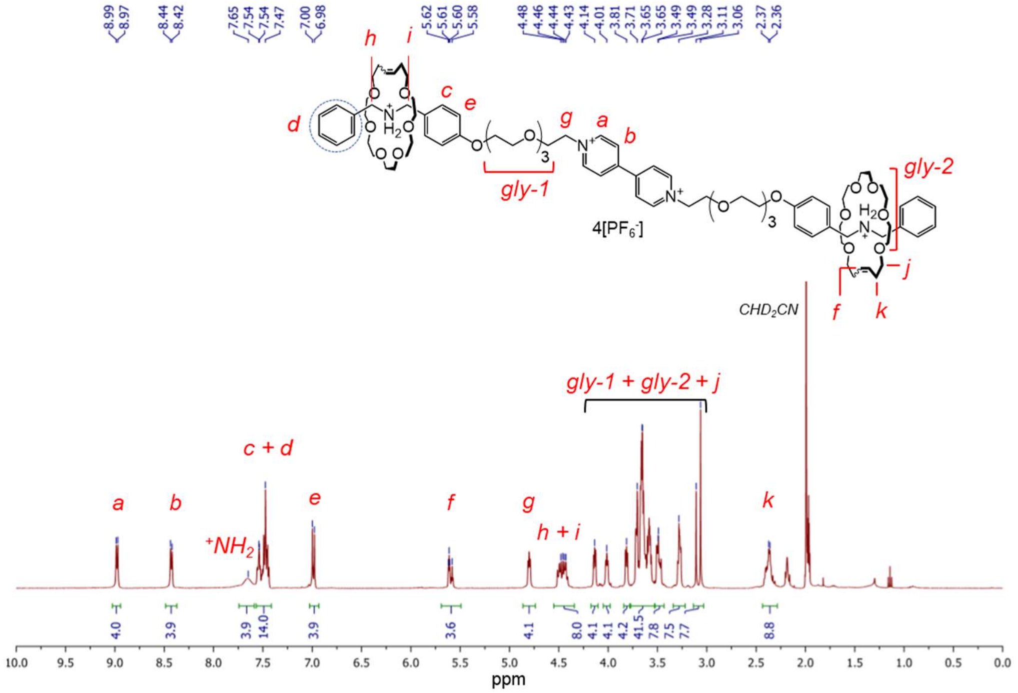

Supplementary Figure 22. ^{1}H NMR spectrum (400 MHz, CD_3CN) of [3]**MSR**

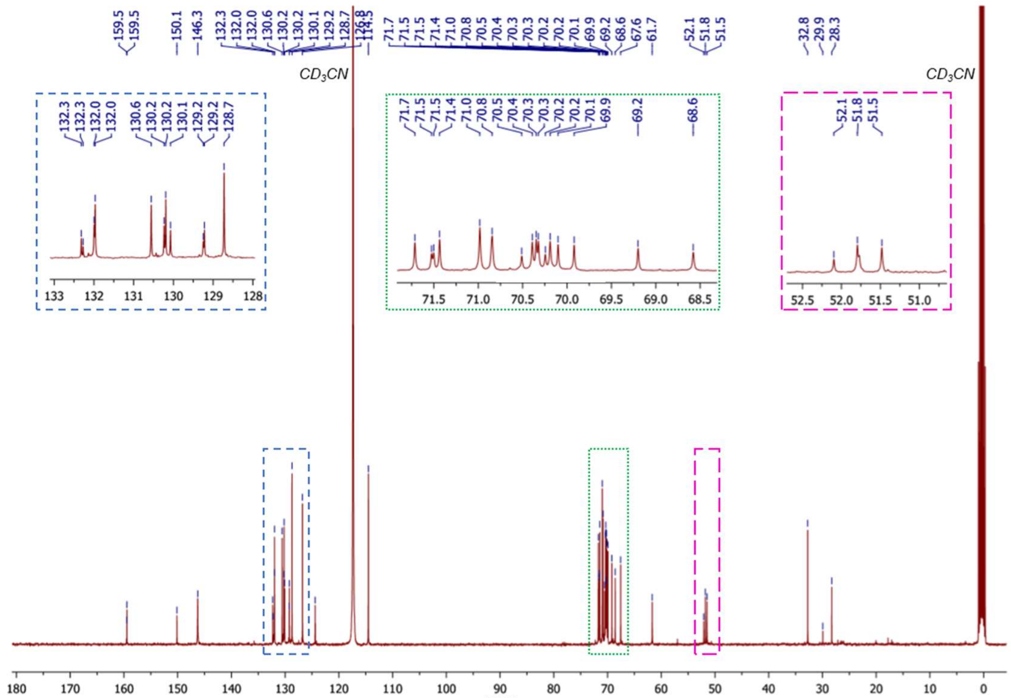

Supplementary Figure 23. ^{13}C NMR spectrum (100 MHz, CD_3CN) of [3]**MSR**

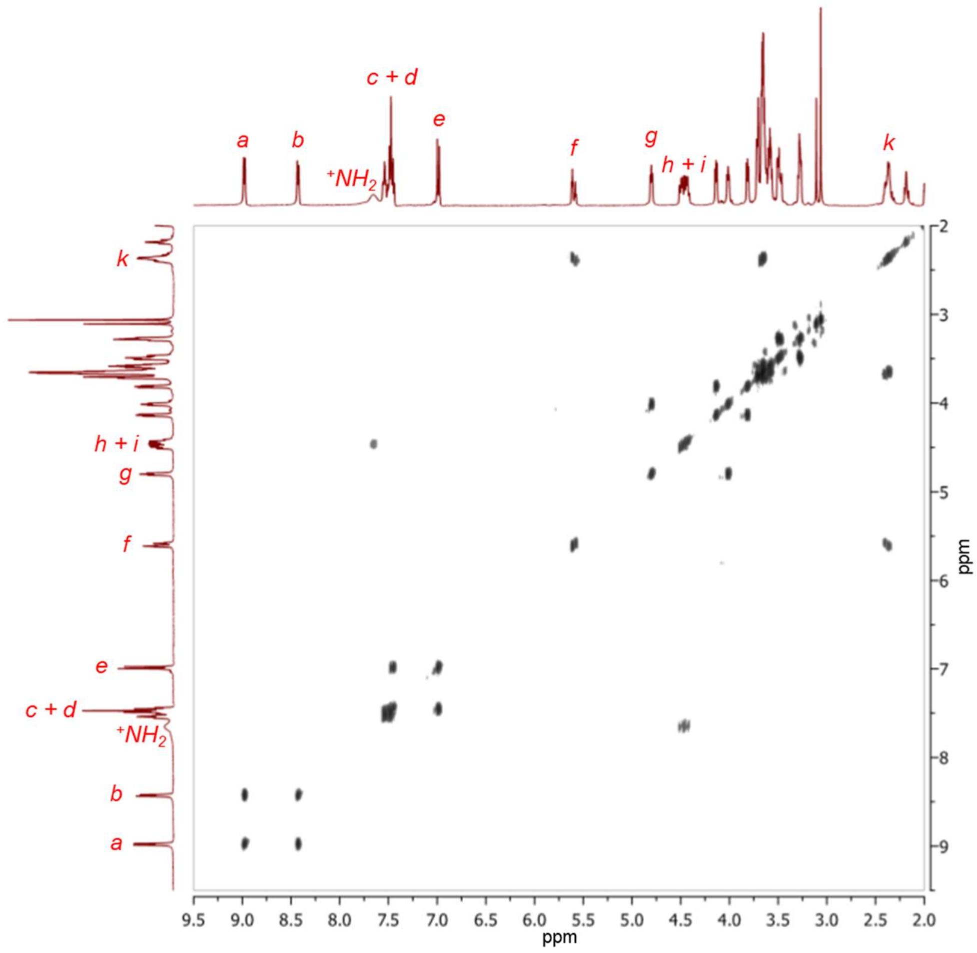

Supplementary Figure 24. ^{1}H-^{1}H COSY NMR spectrum (400 MHz, CD_3CN) of [3]**MSR**

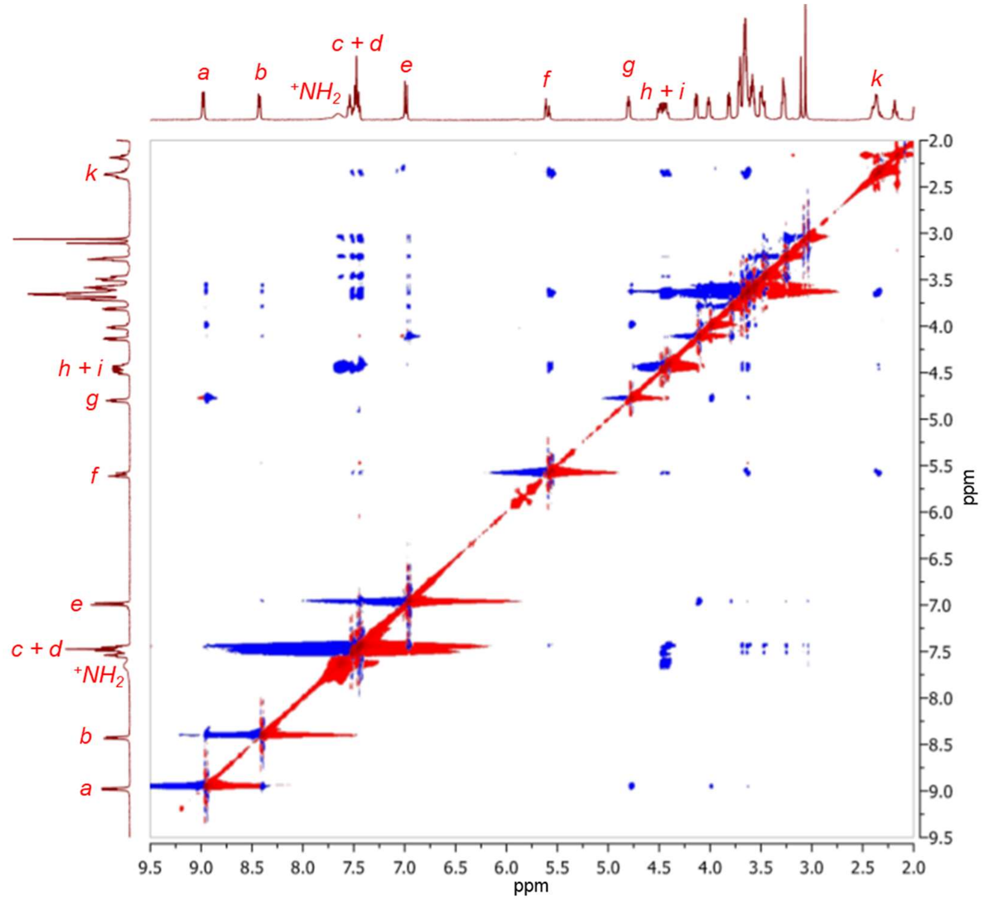

Supplementary Figure 25. ^{1}H-^{1}H NOESY NMR spectrum (400 MHz, CD_3CN) of [3]**MSR**

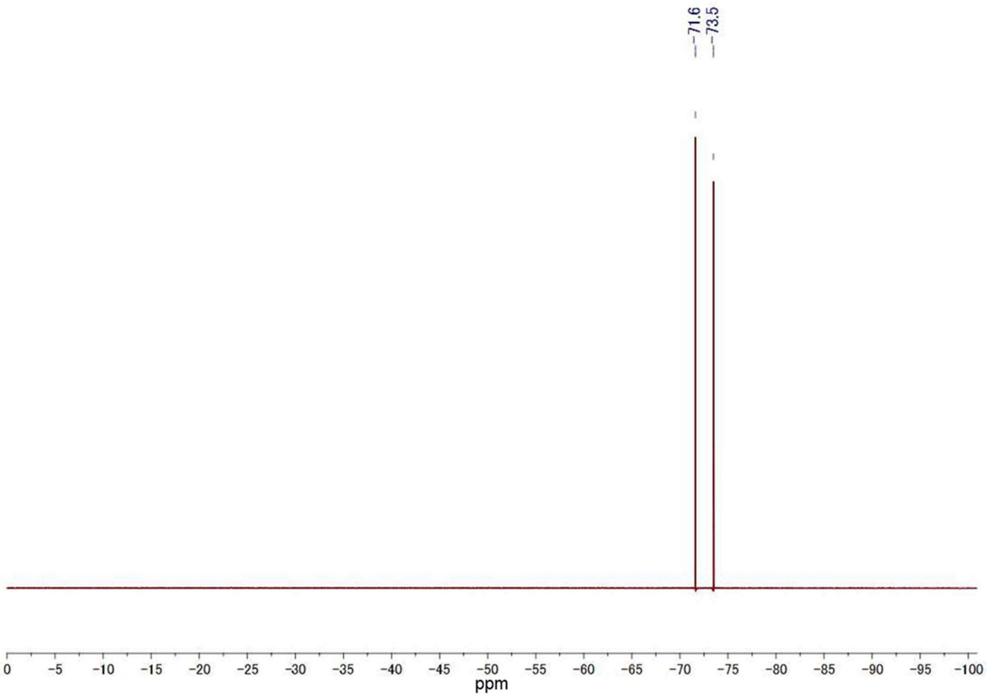

Supplementary Figure 26. ^{19}F NMR spectrum (376 MHz, CD_3CN) of [3]**MSR**

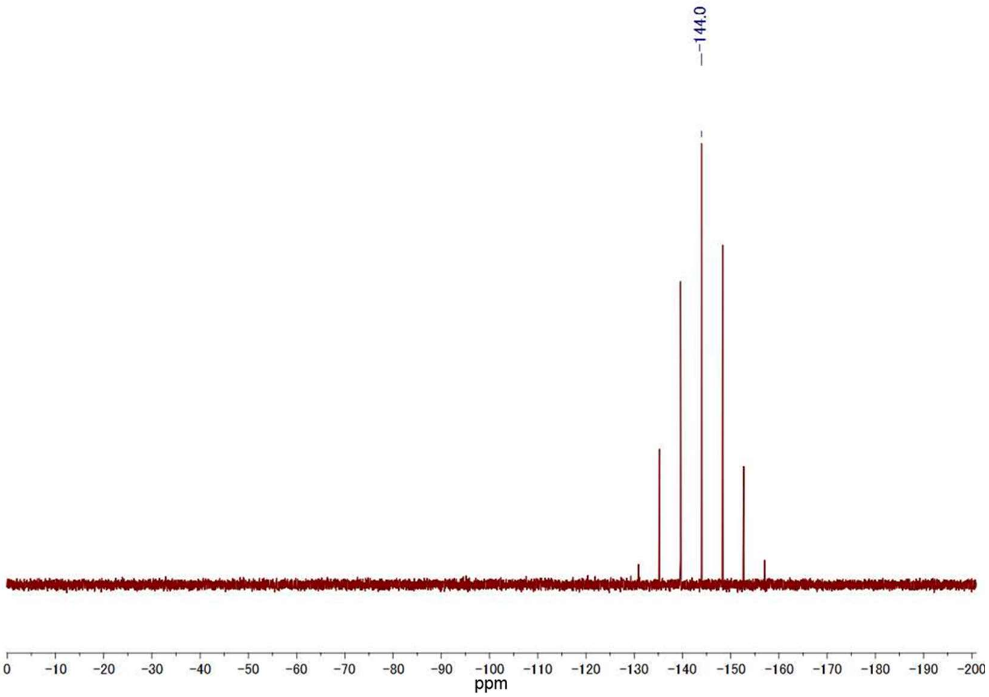

Supplementary Figure 27. ^{31}P NMR spectrum (161 MHz, CD_3CN) of [3]**MSR**

[22]crown-6 ether (22C6)

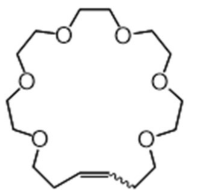

Supplementary Figure 28. Chemical structure of **22C6**

Macrocycle [22]crown-6 ether (**22C6)** was isolated as a byproduct from the synthesis of [3]**MSR**. This crown ether was collected (impure) after column chromatography (SiO_2, CH_2Cl_2/CH_3OH = 92:8 (v/v)). The crude product was subjected to flash column chromatography (SiO_2, EtOAc, R_f = 0.15) to afford the titled product including *cis* (7%)and *trans* (93%) isomers as a colorless oil (0.21 g, 0.64 mmol, 36% yield). ^{1}H NMR (400 MHz, $CDCl_3$) δ = 5.58 – 5.50 (m, 2H), 3.71 – 3.61(m, 20H), 3.57 – 3.51 (m, 4H), 2.41 – 2.28 (m, 4H). ^{13}C NMR (100 MHz, $CDCl_3$) δ = 128.7, 128.0, 71.0, 71.0, 70.9, 70.8, 70.8, 70.7, 70.7, 70.6, 70.6, 70.5, 70.4, 33.1, 28.1. ESI-HRMS: *m*/*z* calculated for [**22C6** + NH_4]$^+$ $C_{16}H_{34}NO_6$, 336.2381; found, 336.2382; relative error 0.3 ppm.

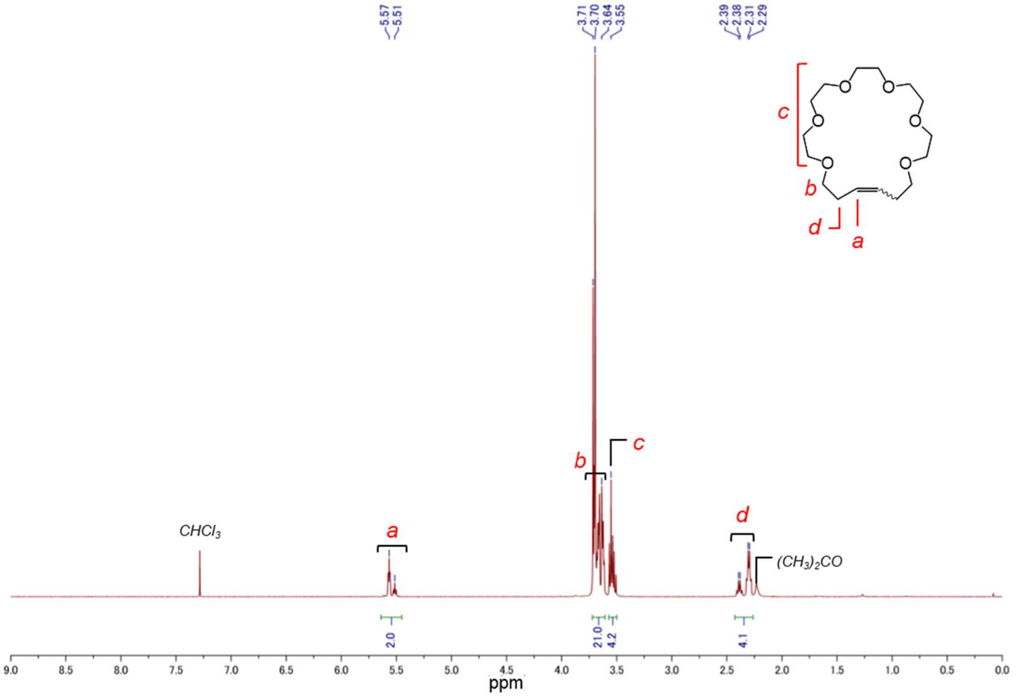

Supplementary Figure 29. ^{1}H NMR spectrum (400 MHz, $CDCl_3$) of **22C6**

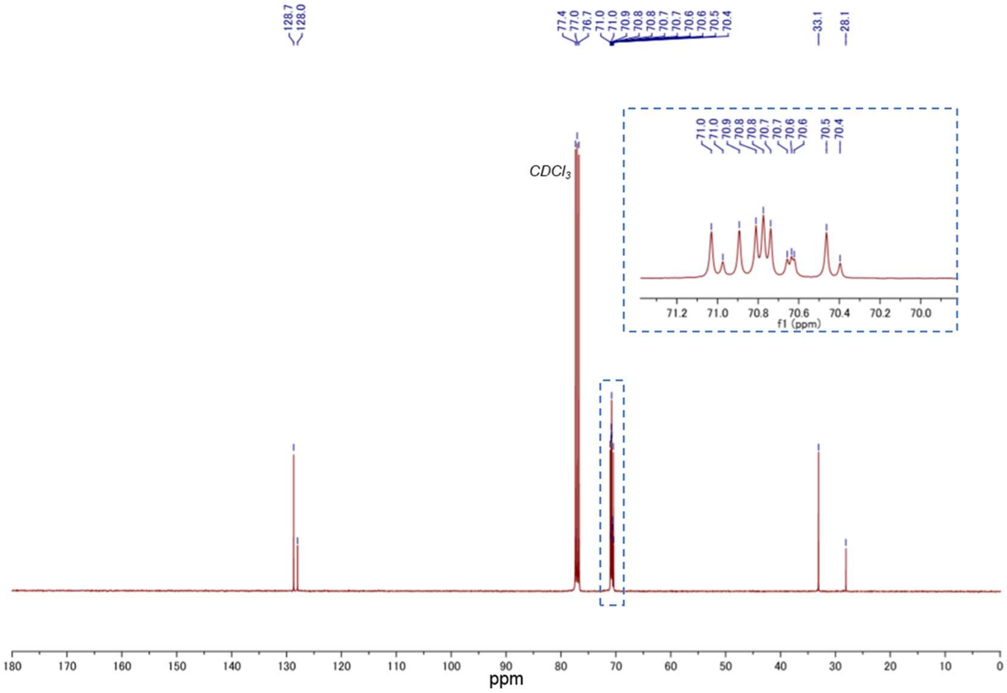

Supplementary Figure 30. ^{13}C NMR spectrum (100 MHz, $CDCl_3$) of **22C6**

Stability test of [3]MSR at room temperature

A solution of [3]**MSR** in dry $CD_3CN/CDCl_3$ (1:1, v/v, 3.5 mM) was prepared and stored at room temperature for 30 days. This sample was periodically analyzed by ^{1}H NMR spectroscopy (Supplementary Figure 31). No significant changes were observed in ^{1}H NMR results over this period, which means [3]**MSR** does not dissociate in solution at room temperature.

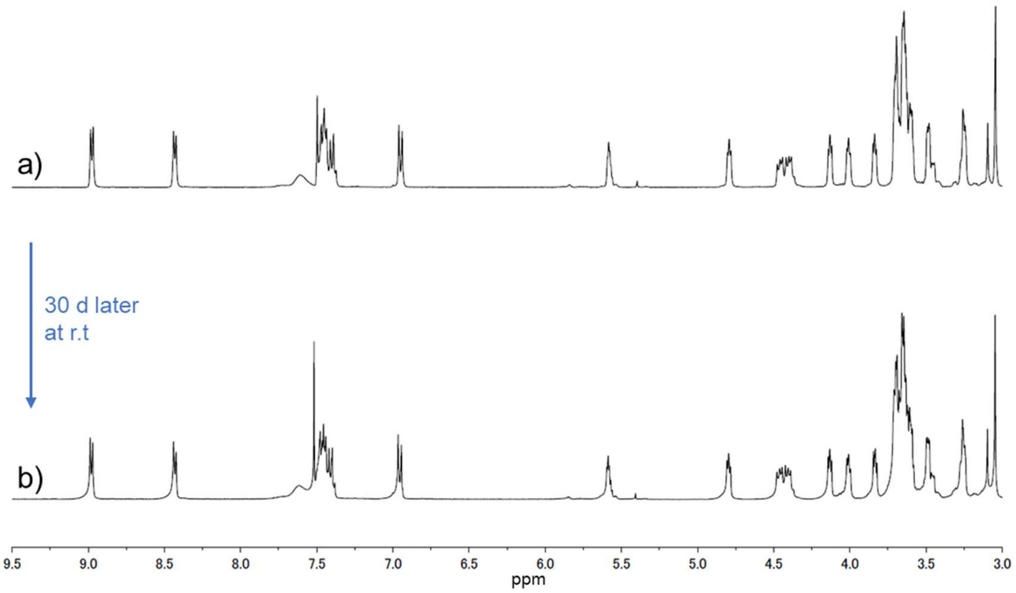

Supplementary Figure 31. Partial ^{1}H NMR spectrum (400 MHz, 298 K, $CD_3CN/CDCl_3$ = 1:1, v/v) of [3]**MSR** a) before and b) after being stored at room temperature for 30 d.

Dissociation tests of [3]MSR

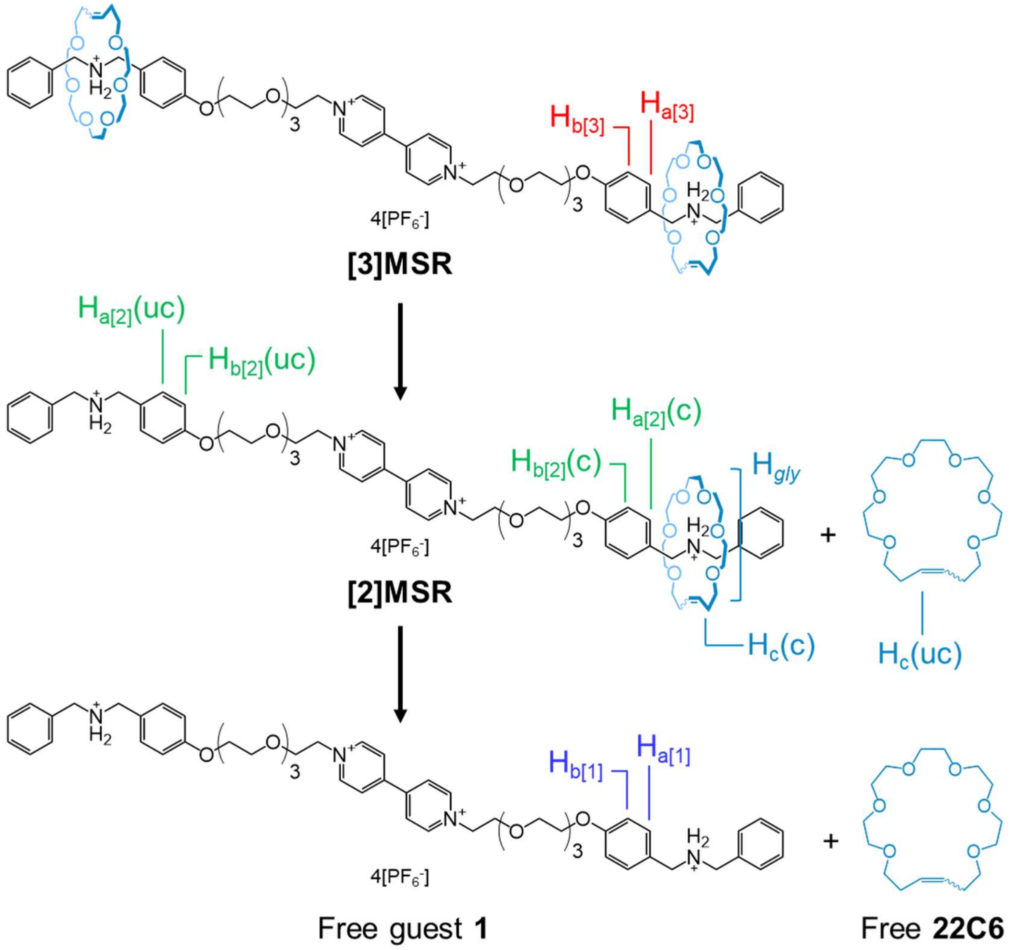

Supplementary Scheme 5. Dissociation of [3]**MSR**

Three independent solutions of [3]**MSR** were prepared with dry $CD_3CN/CDCl_3$ (1:1, v/v, 3.5 mM) in NMR tubes. All systems were tightly capped under the protection of N_2 to prevent moisture intake (a trace amount of water causes precipitation of $\mathbf{1}^{4+}$ when dissociated). We heated the samples at 70 ºC and periodically analyzed them by ^{1}H NMR spectroscopy at 25 ºC. A representative system is shown in Supplementary Figure 32 where two major changes were observed.

First, upon heating, proton signals of *cis*/*trans* H_c on C=C of **22C6** shifted upfield by 0.04 ppm (Supplementary Figure 32). The shifted H_c signals matched with those of free **22C6** indicating that these peak shifts reflect dissociation of **22C6** rings from [3]**MSR**. Second, a single proton peak for DBA moiety of $\mathbf{1}^{4+}$ ($H_{b[3]}$) gradually split into three distinct peaks ($H_{b[2]}$(c), $H_{b[2]}$(uc), and $H_{b[1]}$) during the heating process. While the original $H_{b[3]}$ peak at 6.95 ppm diminished upon heating, one new peak

appeared at an upfield position (6.81 ppm) after 1 d of heating, followed by another peak appearing at a further upfield position (6.77 ppm) on the 4th heating day. The appearance of new peaks at the upfield positions indicates that DBA lost electron-rich **22C6** rings[6]. Focusing on their intensity changes over 30 d heating, while the original $H_{b[3]}$ peak monotonically decreased to almost zero, the first appeared peak increased to reach a maximum on the 10th heating day and decreased after that; on the other hand, the second new signal grew to be dominant. These characteristic intensity changes mean that the original $H_{b[3]}$ peak was converted into the first appeared peak followed by another conversion into the second peak. Considering the order of appearance and positions of the peaks, and overall intensity changes, it is reasonable to explain the observed peak changes as the following process: [3]**MSR** converted the final product (free **1**$^{4+}$) *via* intermediate [2]**MSR**. Based on these points, we assigned the first and second appeared peaks to the protons for uncomplexed DBA of [2]**MSR** ($H_{b[2]}$(uc)) and free **1**$^{4+}$ ($H_{b[1]}$), respectively. It is worth mentioning that one more peak appeared at the same time with $H_{b[2]}$(uc), nearly overlapping with $H_{b[3]}$. This peak's intensity change was identical to that of $H_{b[2]}$(uc) so this peak was assigned to the proton for the remaining rotaxane structure of [2]**MSR** ($H_{b[2]}$(c)). The identical peak splitting was observed in another benzyl proton of DBA ($H_{a[3]}$).

These characterizations were supported by 2-D NMR and ESI-HRMS studies. We analyzed [3]**MSR** solution after 10 d of heating by ^{1}H-^{1}H COSY, NOESY, and EXSY NMR spectroscopy. In ^{1}H-^{1}H COSY NMR (Supplementary Figure 33), we observed 3 sets of cross-peaks between $H_{a[3]}$ + $H_{a[2]}$(c) and $H_{b[3]}$ + $H_{b[2]}$(c), $H_{a[2]}$(uc) and $H_{b[2]}$(uc), and $H_{a[1]}$ and $H_{b[1]}$, which means the protons in the same cross-peak pair are adjacent to each other. Among 3 sets of signals split from $H_{a[3]}$, the mixed-signal of $H_{a[3]}$ + $H_{a[2]}$(c) showed correlations with protons for ethylene glycol chain of **22C6** (H_{gly}) in ^{1}H-^{1}H NOESY NMR (Supplementary Figure 34). The other protons ($H_{a[2]}$(uc) and $H_{a[1]}$), however, did not have correlations with H_{gly}. These observations support DBA moieties holding $H_{a[3]}$ or $H_{a[2]}$(c) are surely surrounded by **22C6** rings, whereas DBA with $H_{a[2]}$(uc) or $H_{a[1]}$ are uncomplexed. Besides, we did not observe chemical exchanges in ^{1}H-^{1}H EXSY NMR (Supplementary Figure 35), which agrees with the fact that all compounds in the solution ([3]**MSR**, [2]**MSR**, free **1**$^{4+}$, and free **22C6**) are non-exchangeable species. Final evidence was provided by ESI-HRMS analysis. We detected [3]**MSR**, [2]**MSR**, free **1**$^{4+}$, and free **22C6** in the samples after 30 d of heating as follows: m/z calculated for [[3]**MSR**]$^{4+}$ $C_{86}H_{130}N_4O_{20}$, 384.9823; found, 384.9832. m/z calculated for [[2]**MSR**]$^{4+}$ $C_{70}H_{100}N_4O_{14}$, 305.1804; found, 305.1818. m/z calculated for [**1** – 2H]$^{2+}$ $C_{54}H_{68}N_4O_8$, 450.2513; found, 450.2515. m/z calculated for [**22C6** + NH_4]$^+$ $C_{16}H_{34}NO_6$, 336.2381; found, 336.2383(Supplementary Figure 36). Thus, all results here supported the thermal dissociation of [3]**MSR** proceeds along with Supplementary Scheme 5.

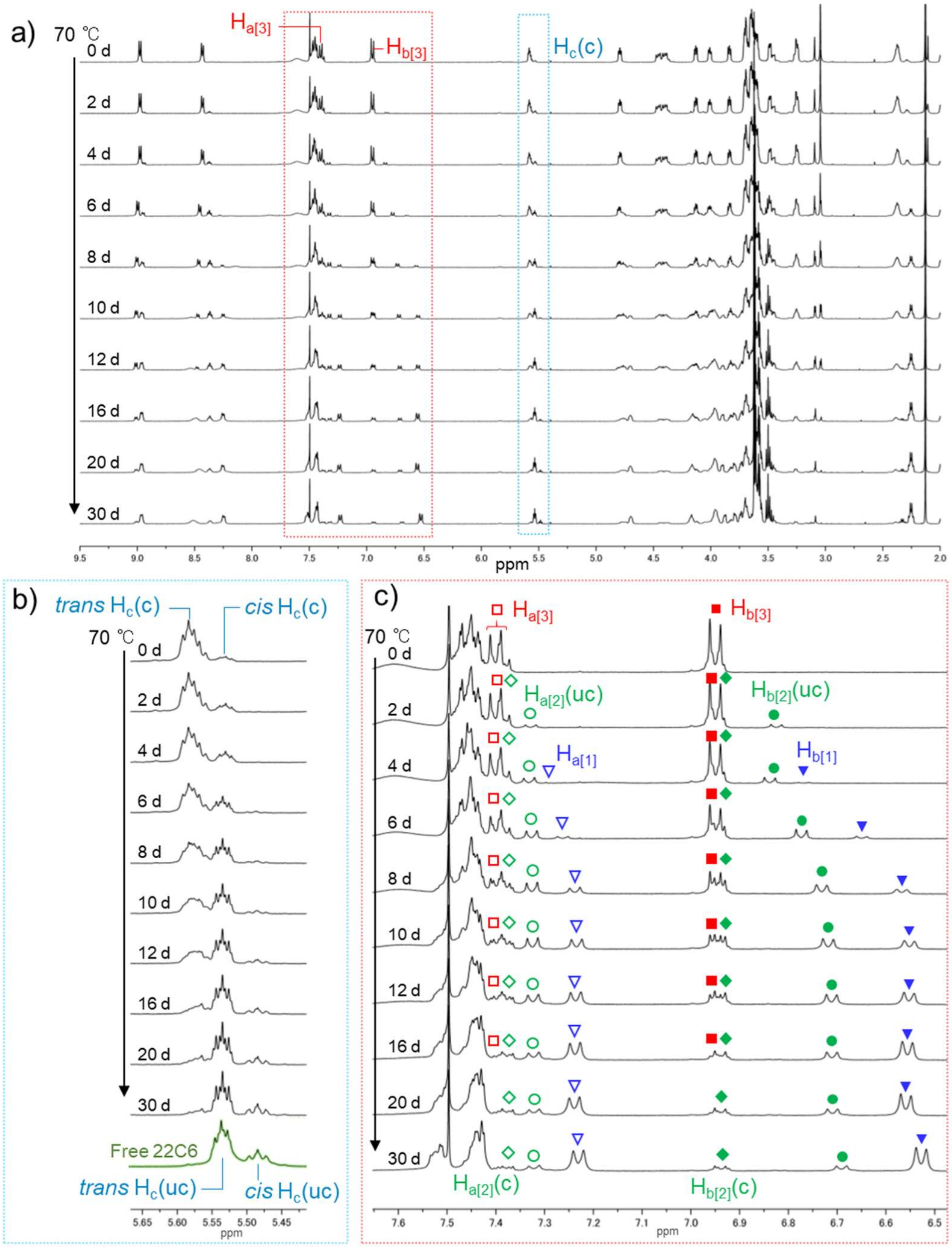

Supplementary Figure 32. Representative partial ^{1}H NMR spectra of [3]**MSR** heated at 70 ºC for 30 d (400 MHz, $CD_3CN/CDCl_3$ = 1:1, v/v, 3.5 mM) for dissociation. a) Full view showing changes over the heating time. b) Zoom-in view focusing on H_c. The bottom green spectrum is isolated free **22C6** as a reference, and the others are spectra depending on heating time. H_c had *trans* and *cis* isomers. (c) and (uc) denote complexed and uncomplexed (dissociated), respectively. c) Zoom-in spectra view focusing on protons on DBA moiety (H_a and H_b): suffix [3], [2], and [1] represent [3]**MSR**, [2] **MSR**, and free **1**$^{4+}$, respectively. The same symbols on the peaks support tracking the highlighted peak changes.

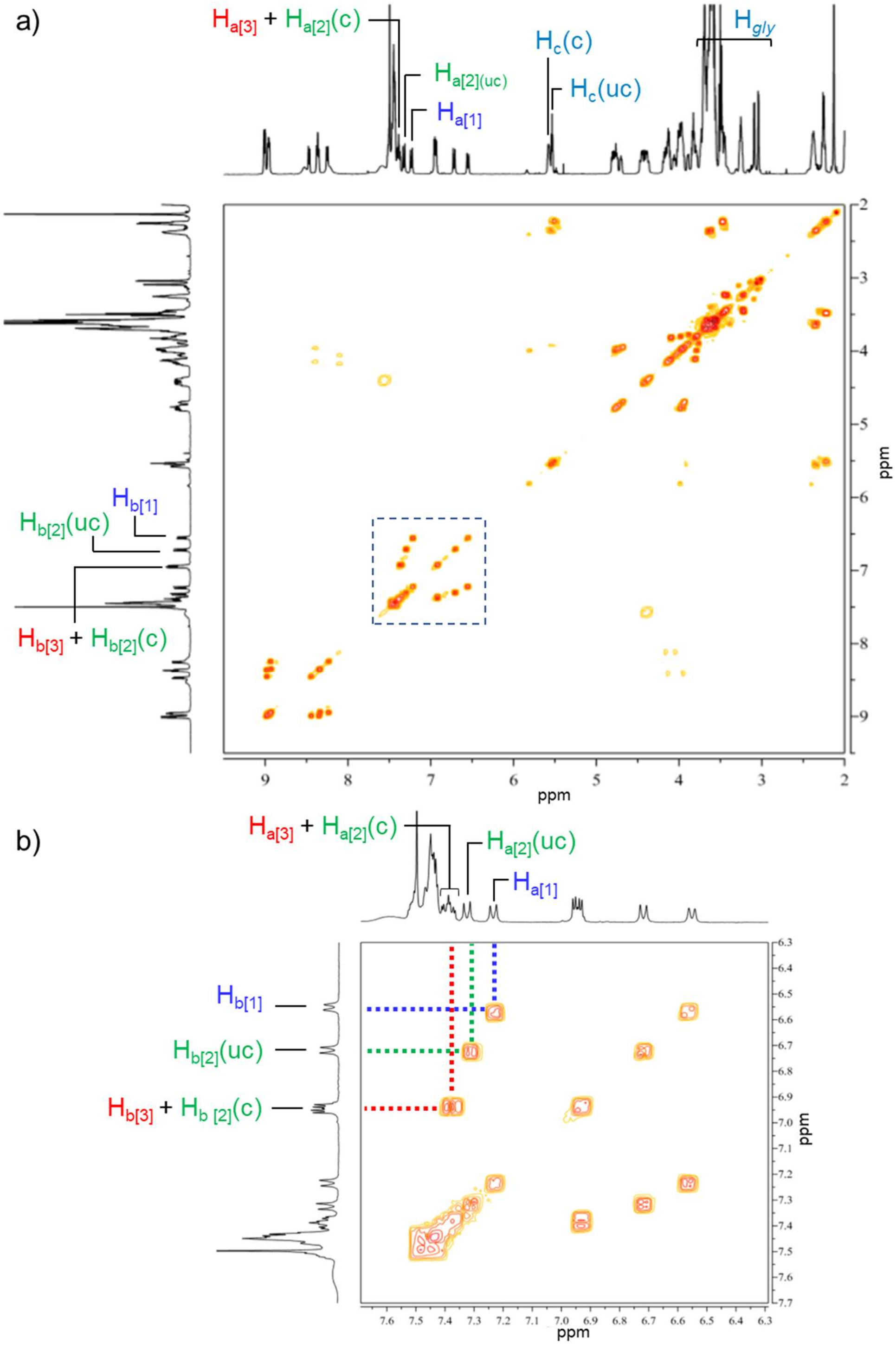

Supplementary Figure 33. ^{1}H-^{1}H COSY NMR spectrum (400 MHz, $CD_3CN/CDCl_3$ =1:1, v/v) of [3]**MSR** heated at 70 ºC for 10 days under N_2 atmosphere. a) Full and b) Zoom-in view on the square area in a); dash lines indicate correlations.

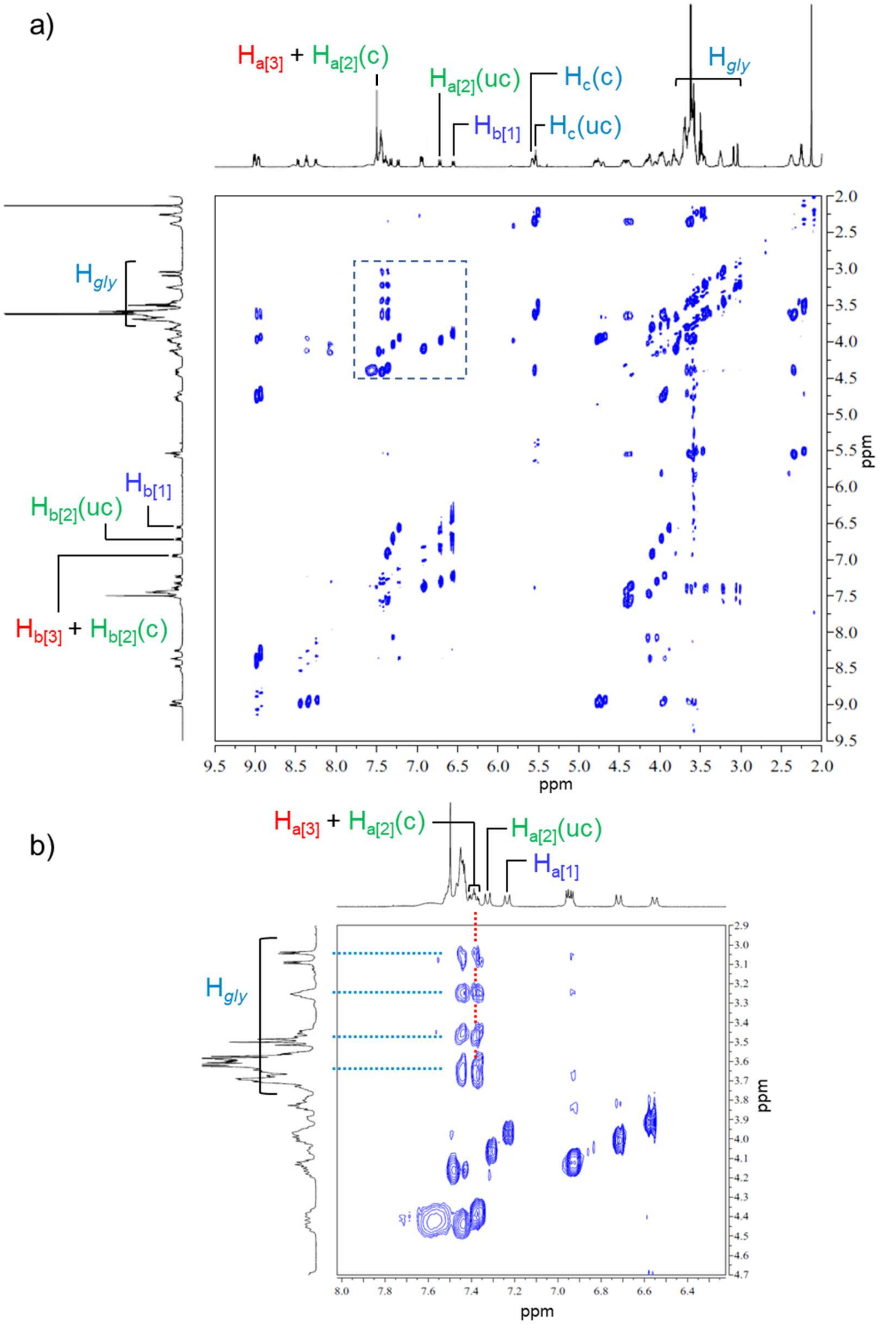

Supplementary Figure 34. ^1H-^1H NOESY NMR spectrum (400 MHz, $CD_3CN/CDCl_3$ =1:1, v/v) of

[3]**MSR** heated at 70 ºC for 10 days under the N_2 atmosphere. a) Full and b) Zoom-in view on the square area in a); dash lines indicate correlations.

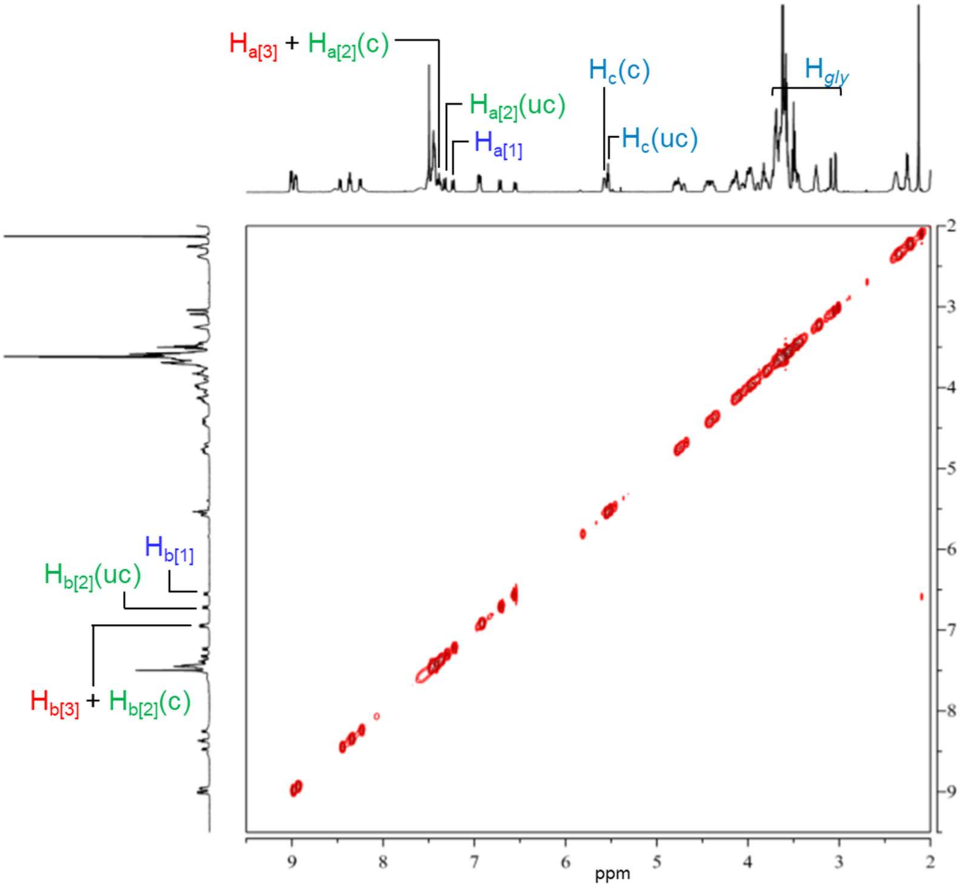

Supplementary Figure 35. ^1H-^1H EXSY NMR spectrum (400 MHz, $CD_3CN/CDCl_3$ =1:1, v/v) of [3]**MSR** heated at 70 ºC for 10 days under N_2 atmosphere.

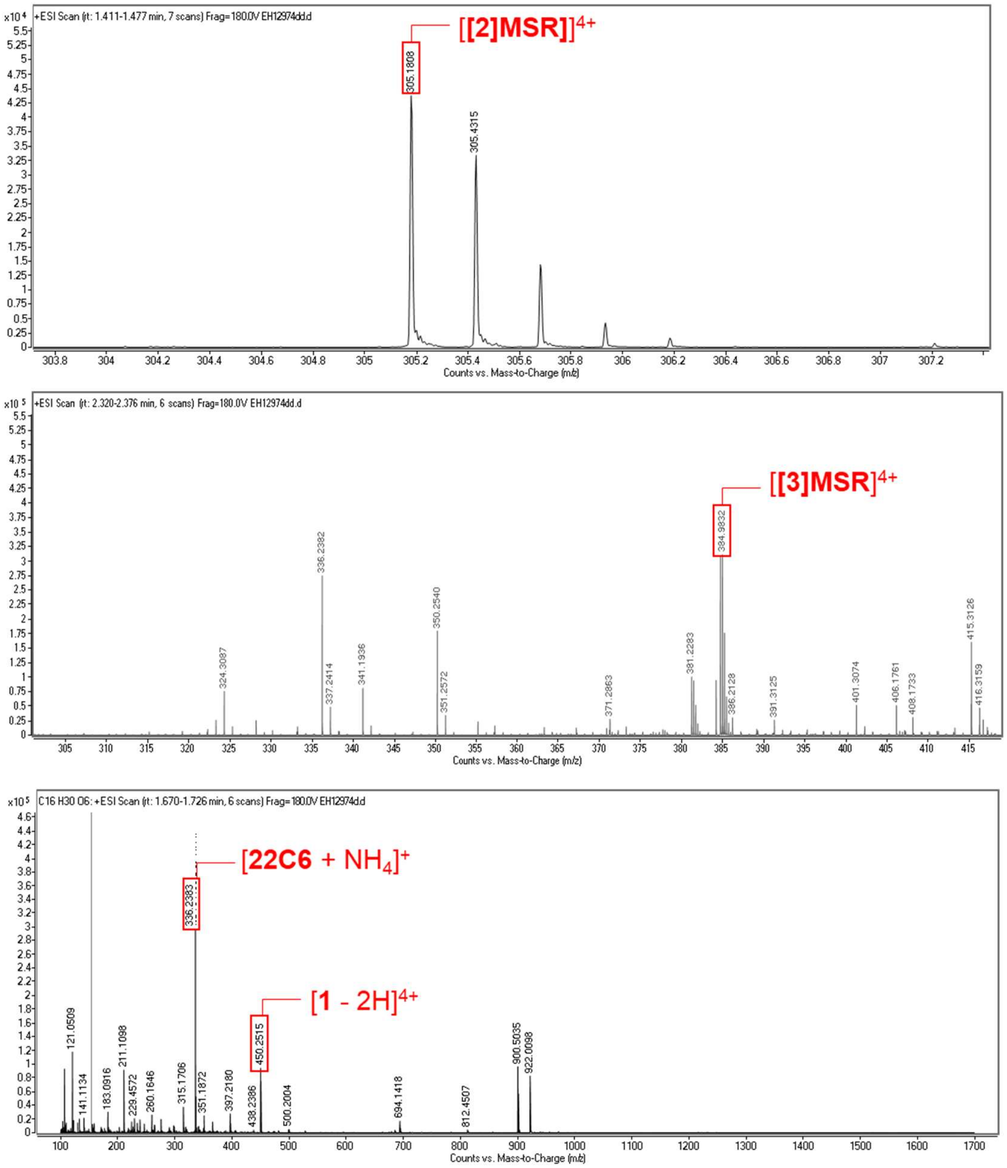

Supplementary Figure 36. Partial ESI high-resolution mass spectrum of [3]**MSR** solution heated at 70 ºC for 30 days under the N_2 atmosphere for dissociation test.

Supplementary note 1

For a better understanding, we carried out two quantitative analyses of the dissociation process of [3]**MSR**. We first estimated the dissociation ratio of **22C6** rings depending on the heating time. The dissociation process of **22C6** was tracked by the peak shift from the complex form (*c*) to the uncomplexed (*uc*) form in ^{1}H NMR spectra (Supplementary Figure 32b). We calculated the dissociation ratio using the integration of proton H_c from Equation (11), which was derived as follows:

The dissociation ratio of **22C6** after *n* days of heating was defined as:

$$\text{Dissociation ratio}_{(n)} = \frac{\mathrm{I}\left(trans\,H_c\,(uc)_{(n)}\right) + \mathrm{I}\left(cis\,H_c\,(uc)_{(n)}\right)}{\mathrm{I}\left(\sum H_{c\,(n)}\right)}$$ …Equation (3)

Where,

(n): heating days at 70 ºC;

c, uc: complexed and uncomplexed

trans H_c, *cis* H_c: proton species of **22C6** assigned in Supplementary Figure 37a

$\Sigma\, H_{c\,(n)}$: H_c in all state, i.e. both *trans*/*cis* in *c* and *uc* states

I (X): the integration of X resonance

The signals of H_c in ^{1}H NMR appeared in three different chemical shift areas depending on its state (*trans*/*cis* in *c* and *uc* states): zone A, B, and C designated in Supplementary Figure 37. The integrations of protons for *trans* H_c(c) and *cis* H_c(uc) isolated in zone A and C, respectively, are measurable, but those of *trans* H_c(uc) and *cis* H_c(c) cannot be measured separately because they overlapped in zone B. To calculate the dissociation ratio using the integrations from each zone, we rearranged Equation (3) to Equation (11) by applying Equation (4) – (10).

$\int \boldsymbol{zone\ A_{(n)}} = \boldsymbol{I\left(trans\ H_c(c)_{(n)}\right)}$ …Equation (4)

$\int \boldsymbol{zone\ B_{(n)}} = \boldsymbol{I\left(cis\ H_c\ (c)_{(n)}\right) + I\left(trans\ H_c(uc)_{(n)}\right)}$ …Equation (5)

$\int \boldsymbol{zone\ B_{(0)}} = \boldsymbol{I\left(cis\ H_c(c)_{(0)}\right)}$ …Equation (6)

$\int zone\ C_{(n)} = I\left(cis\ H_c(uc)_{(n)}\right)$ …Equation (7)

$\int zone\ C_{(0)} = I\left(cis\ H_c(uc)_{(0)}\right) = 0$ …Equation (8)

$\int zone\ A_{(n)} + \int zone\ B_{(n)} + \int zone\ C_{(n)} = I\left(\sum H_{c\,(n)}\right)$ …Equation (9)

Where,

$\int zone\ A_{(n)}$, $\int zone\ B_{(n)}$, $\int zone\ C_{(n)}$

: the integral of proton signals in zone A, B, and C, respectively.

Focusing on the molar balance of *cis* H_c, Equation (10) was prepared.

$\mathrm{I}\left(cis\ H_c(\mathrm{c})_{(n)}\right) = \mathrm{I}\left(cis\ H_c(c)_{(0)}\right) - \mathrm{I}\left(cis\ H_c(uc)_{(n)}\right)$ …Equation (10)

Here,

$$\text{Dissociation ratio}_{(n)} = \frac{I\left(trans\,H_c\,(uc)_{(n)}\right) + I\left(cis\,H_c\,(uc)_{(n)}\right)}{\mathrm{I}\left(\sum H_{c\,(n)}\right)}$$

$$= \frac{\int zone\, B_{(n)} + \int zone\, C_{(n)} - I\left(cis\, H_c\, (c)_{(n)} - cis\, H_c\, (uc)_{(n)}\right)}{\int zone\, A_{(n)} + \int zone\, B_{(n)} + \int zone\, C_{(n)}}$$

$$= \frac{\int zone\, B_{(n)} + 2 \times \int zone\, C_{(n)} - I\left(cis\, H_c\, (c)_{(0)}\right)}{\int zone\, A_{(n)} + \int zone\, B_{(n)} + \int zone\, C_{(n)}}$$

$$= \frac{\int zone\, B_{(n)} + 2 \times \int zone\, C_{(n)}}{\int zone\, A_{(n)} + \int zone\, B_{(n)} + \int zone\, C_{(n)}} + \frac{\int zone\, C_{(0)}}{\int zone\, A_{(0)} + \int zone\, B_{(0)} + \int zone\, C_{(0)}} \times \frac{\int zone\, A_{(0)} + \int zone\, B_{(0)} + \int zone\, C_{(0)}}{\int zone\, A_{(n)} + \int zone\, B_{(n)} + \int zone\, C_{(n)}}$$

Equation (11)

We calculated the dissociation ratio from Equation (11) for the three independent samples and plotted the results in Supplementary Figure 37b. The dissociation became slow around 20 d of heating and converged on 30 d.

Next, we quantitatively analyzed the conversion flow of [3]**MSR** to free $\mathbf{1}^{4+}$ *via* intermediate [2]**MSR** focusing on proton H_b (Supplementary Figure **32**b). In the collected NMR spectra, peaks for $H_{b[2]}$(uc) and $H_{b[1]}$ existed independently, but $H_{b[3]}$ overlapped $H_{b[2]}$(uc). To calculate the integrations of individual H_b species, we divided chemical shift area into three zones (D, E, and F) as Supplementary **Figure 38** shows and calculated the existence ratio of [3]**MSR**, [2]**MSR**, and free $\mathbf{1}^{4+}$ from Equation (19) derived the following process.

The relationships of integrations between zones and H_b species were defined as Equation (12)-(16).

$\int zone\, D_{(n)} = I(H_{b[2]}(c)_{(n)}) + I(H_{b[3](n)})$…Equation (12)
$\int zone\, E_{(n)} = I\left(H_{b[2]}(uc)_{(n)}\right)$ …Equation (13)
$\int zone\, E_{(0)} = 0$ …Equation (14)
$\int zone\, F_{(n)} = I\left(H_{b[1](n)}\right)$…Equation (15)
$\int zone\, F_{(0)} = 0$ ….Equation (16)

Where,
$\int zone\, X$: integral of proton in zone X (X = D, E or F)
[1], [2], [3]: $\mathbf{1}^{4+}$, [2]**MSR**, [3]**MSR**
(n): heating days at 70 ºC;
I ($H_{b[Y](n)}$): integral of $H_{b\,[Y]}$ (Y = 1, 2, or 3) resonance after n days of heating
c, uc: complexed and uncomplexed

Here, Equation (17) holds because integrations of $H_{b[2]}(uc)_{(n)}$ and $H_{b[2]}(c)_{(n)}$ should be equal because they are equimolar protons on the same compound.

$I(H_{b[2]}(uc)_{(n)}) = I(H_{b[2]}(c)_{(n)})$ …Equation (17)

The integration of [3]**MSR** can be described as Equation (18) by combining Equation (16) and (17).

$I(H_{b[3](n)}) = \int zone\ D_{(n)} - \int zone\ E_{(n)}$ …Equation (18)

Using the results derived above process, the existence ratio of compound [Y] (Y = [3]**MSR**, [2]**MSR**, or **1**$^{4+}$) was calculated from Equation (19).

$Existence\ ratio_{[Y](n)} = \frac{I(H_{b[Y](n)})}{I(\sum H_{b(n)})}$ … Equation (19)

Where,
Σ H_b: H_b in all states, i.e. [3]/[2]/[1] in c and uc
$I(\sum H_{b(n)}) = \int zone\ D_{(n)} + \int zone\ E_{(n)} + \int zone\ F_{(n)}$

The calculated existence ratio was plotted in Supplementary Figure 38b. After 30 d of heating, almost all [3]**MSR** was consumed and 80% of [3]**MSR** was converted into free **1**$^{4+}$.

To estimate the reliability of these two analyses, we calculated the dissociation ratio of **22C6** differently from Equitation (11) by using the obtained existence ratio of [3], [2]**MSR**, and free **1**$^{4+}$. The [3]**MSR** equips with two **22C6** rings and releases one of **22C6** converting into [2]**MSR**, which releases the other **22C6** providing free **1**$^{4+}$. This process means that the equivalence of dissociated **22C6** is one and two to [2]**MSR** and free **1**$^{4+}$, respectively. Based on this relationship, the dissociation ratio of **22C6** is estimated by Equation (20):

$$Dissociation\ Ratio_{[n]} = Existence\ Ratio_{[2]\ (n)\times} \times \frac{1}{2} + Existence\ Ratio_{[1]\ (n)}$$

…Equation (20)

The plots of the dissociation ratio calculated from Equation (20) agreed with those from Equation (11) indicating that these quantitative analyses are highly reliable (Supplementary Figure 37).

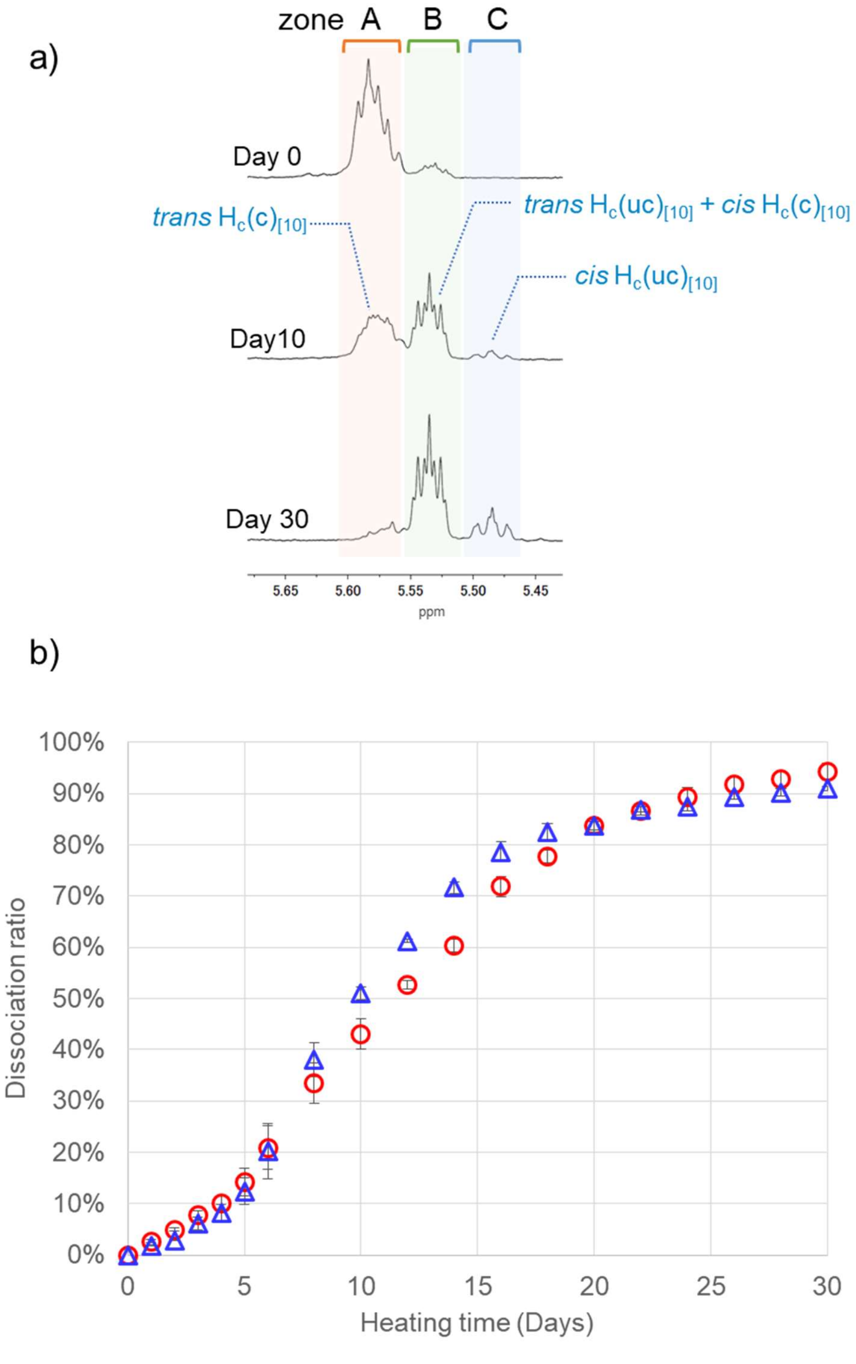

Supplementary Figure 37. 22C6 release *via* dissociation of [3]**MSR**. a) Partial ^{1}H NMR spectra before heating (top), after 10 d of heating (middle), after 30 d of heating (bottom). b) Kinetic analysis of the disassembly of **22C6** calculated from Equation (11) (red) and Equation (20) (blue)(average of three independent samples).

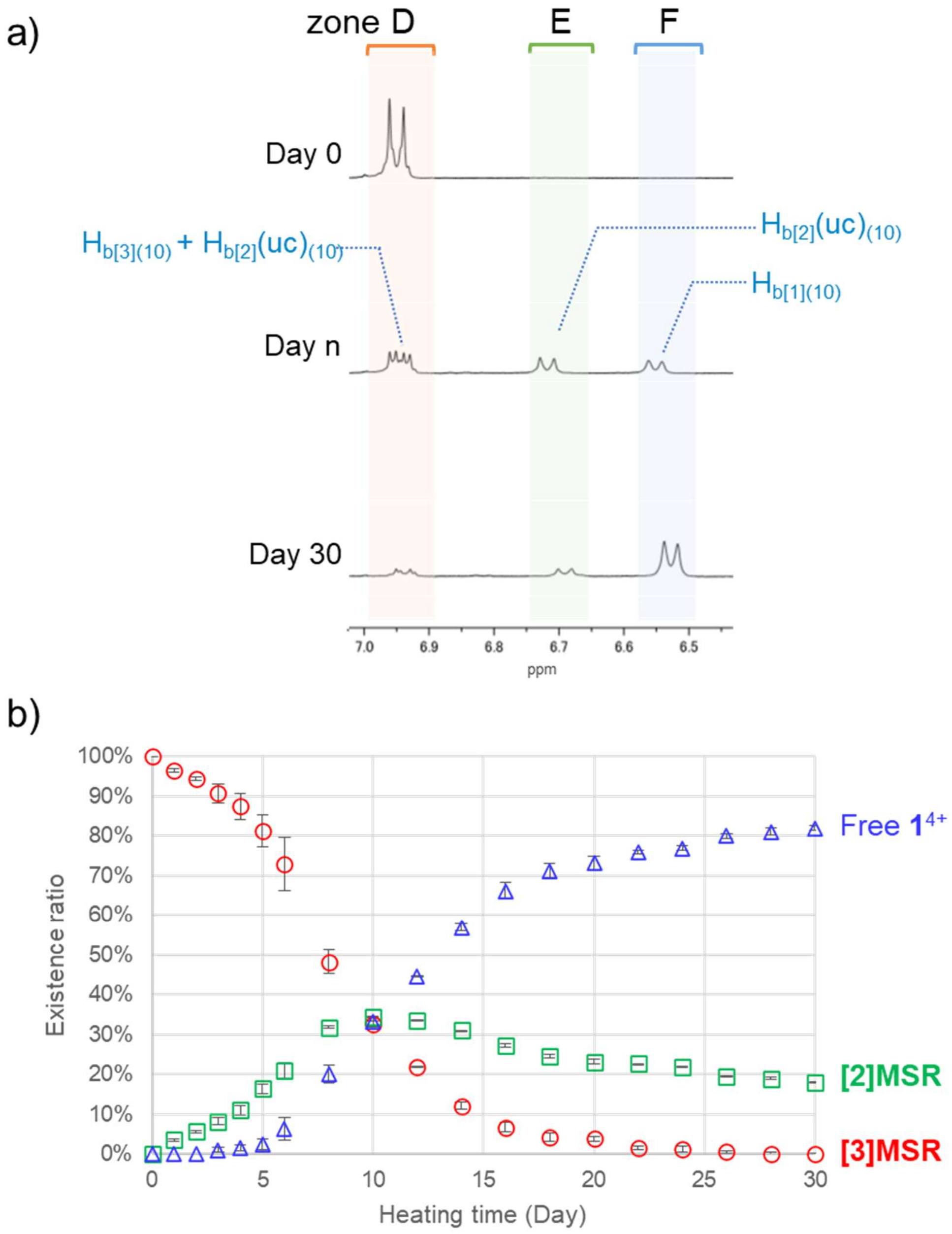

Supplementary Figure 38. Guest release *via* dissociation of [3]**MSR**. a) Partial ^{1}H NMR spectra before heating (top), after 10 d of heating (middle), after 30 d of heating (bottom). b) Kinetic analysis of the disassembly of [3]**MSR** (average of three independent samples): [3]**MSR** (red), [2]**MSR** (green), and $\mathbf{1}^{4+}$ (blue).

Stability test of dissociation process of [3]MSR

To confirm that the dissociation process of [3]**MSR** is irreversible, a solution of [3]**MSR** in dry $CD_3CN/CDCl_3$ (1:1, v/v, 3.5 mM) was heated at 70 ºC for 12 days and then cooled down and left at r.t for 20 days. Subsequently, the sample was heated at 70 ºC for another 22 days (30 days of heating in total) and then stored at r.t for 1 year. After each treatment, the sample was analyzed by ^1H NMR spectroscopy (Supplementary Figure 39). In the collected ^1H NMR spectra, we observed dissociation progressed in the heating process but did not observe any significant changes during the storage period at room temperature. This means the thermal-triggered dissociation process is irreversible and can stop and restart by turning off/on the heating.

On the other hand, a sample containing dibenzylammonium (DBA) hexafluorophosphate and *cis*/*trans*-**22C6** in equimolar amounts (5×10^{-3} M), in $C_2D_2Cl_4$, was analized by ^1H NMR spectroscopy (Figure S38e); the experiment showed no self-assembly, suggesting that the **22C6** ring cannot slip over the phenyl rings contained on DBA at ambient conditions. Even after heating at 130 °C for 7 d, we did not detect diagnostic signals for rotaxane assembly (Figure S38f).

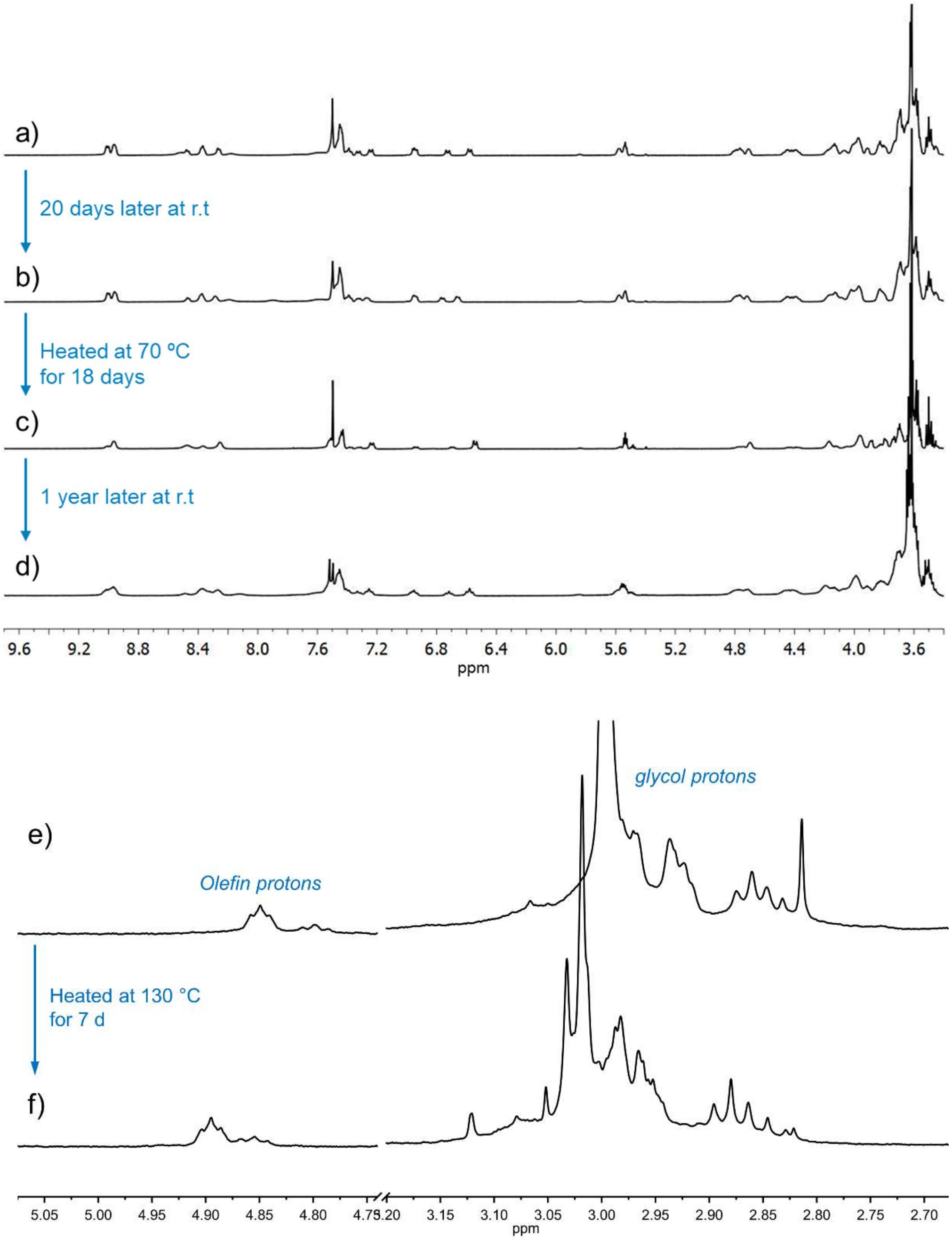

Supplementary Figure 39. ^{1}H NMR spectrum (400 MHz, 298 K, $CD_3CN/CDCl_3$ = 1:1, v/v) of [3]**MSR** a) heated at 70 ºC for 12 d, b) stored at r.t for 20 d after a), c) heated at 70 ºC for another 18 d after b), and d) stored at r.t for 1 year after c). Partial ^{1}H NMR spectra (400 MHz, 298 K, $C_2D_2Cl_4$) of a sample containing DBA[PF_6] and *cis*/*trans*-**22C6** (5×10^{-3} M, 1:1 mol ratio), e) before and f) after heating for 7 d at 130 °C. Spectra show resonances for the *cis*/*trans*-**22C6** species.

Ring-exchange from metastable rotaxane to pseudorotaxane

We investigated thermal-triggered ring-exchange from [3]**MSR** to a set of pseudorotaxanes: [2]**PR** [$\mathbf{1}^{4+}$ ⊂ **DN38C10**], [3]**PR** [$\mathbf{1}^{4+}$ ⊂ (**DB24C8**)$_2$], and [4]PR [**DN38C10** ⊃ $\mathbf{1}^{4+}$ ⊂ (**DB24C8**)$_2$]. Three independent solutions of [3]**MSR** (3.5 mM) with the corresponding ring(s): **DN38C10** (3.5 mM), **DB24C8** (7.0 mM), and **DN38C10** (3.5 mM)/**DB24C8** (7.0 mM), in mixed dry solvent ($CD_3CN/CDCl_3$ = 1:1, v/v) were separately prepared in tightly capped NMR tubes purged with N_2. Likewise the dissociation studies of [3]**MSR**, The samples were heated at 70 ºC up to 30 d and periodically monitored by ^{1}H NMR spectroscopy.

The mixtures of [3]**MSR** and free rings, **DN38C10** and/or **DB24C8**, did not produce any pseudorotaxanes. When heating the systems, however, all ring-exchange systems toward [2], [3], or [4]pseudorotaxanes were successfully performed, involving the disassembly of [3]**MSR** and the following self-assembly with prepared rings (See "Ring-exchange from [3]metastable rotaxane to [4]pseudorotaxane" section for detailed characterization).

Ring-exchange from [3]MSR to [2]pseudorotaxane

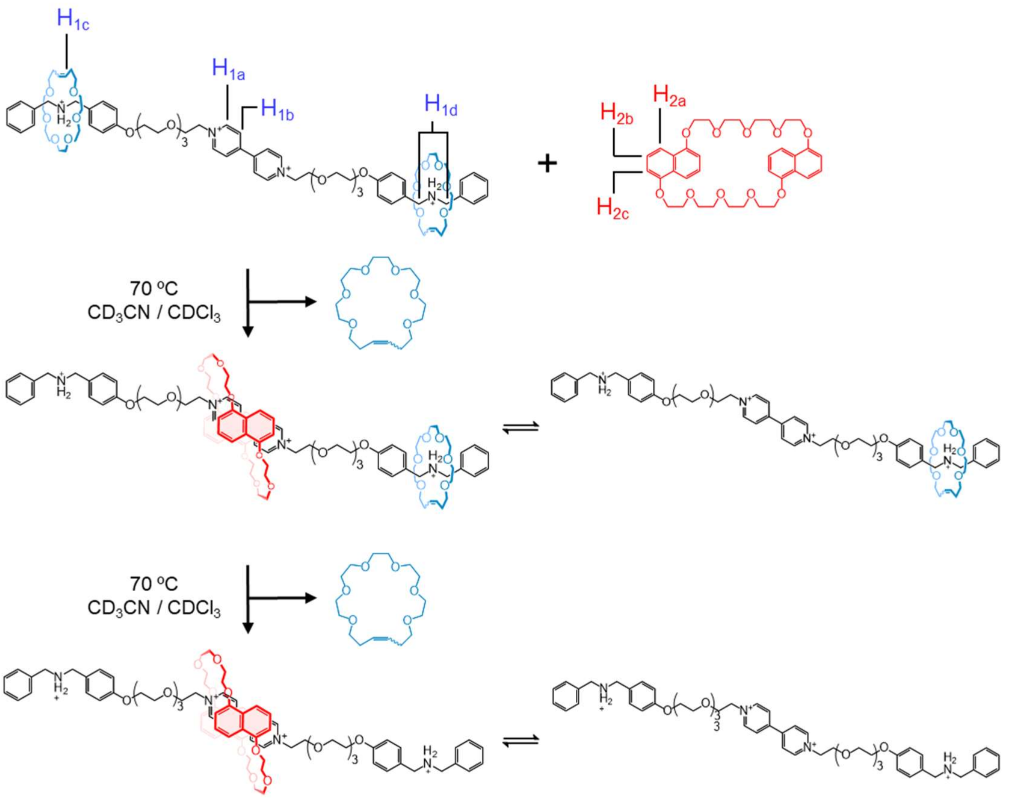

Supplementary Scheme 6. [2]**PR** formation from [3]**MSR** via thermal-triggered ring-exchange

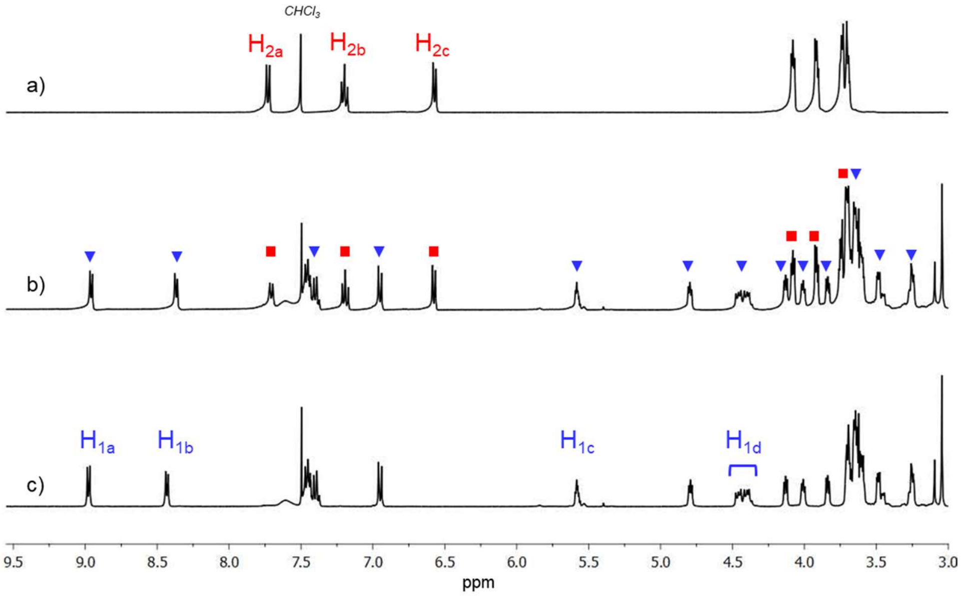

Supplementary Figure 40. Partial ^{1}H NMR spectra (400 MHz, CD_3CN / $CDCl_3$ = 1:1 (v/v), 3.5 mM). a) **DN38C10**, c) [3]**MSR**, and c) a 1:1 mixture of [3]**MSR** and **DN38C10**. The markers on b) shows the origin of the signals: **DN38C10** (red) and [3]**MSR** (blue).

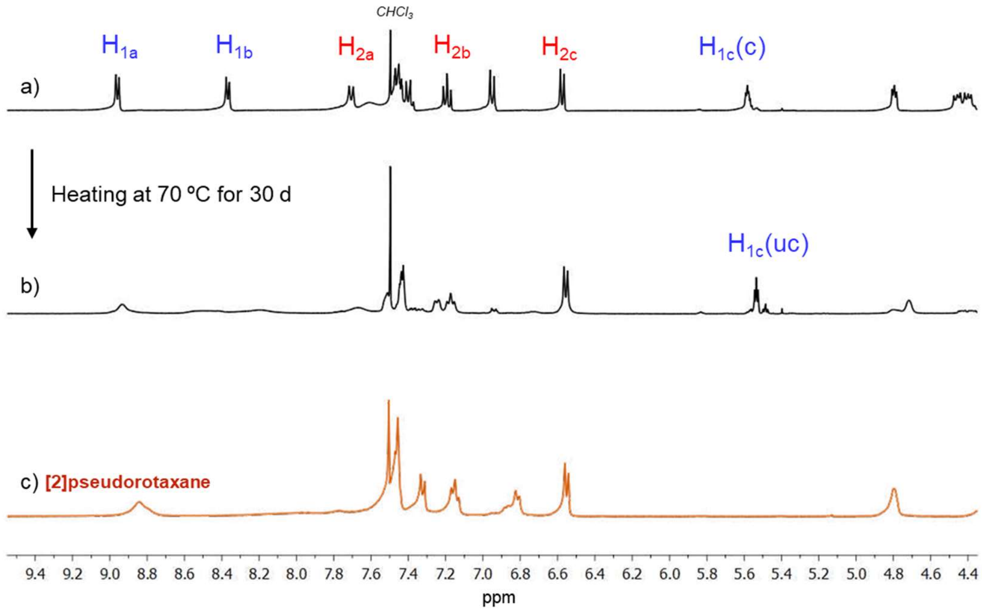

Supplementary Figure 41. Partial ^{1}H NMR spectra (400 MHz, dry CD_3CN / $CDCl_3$ = 1:1, v/v, 3.5 mM). Mixture of [3]**MSR** and **DN38C10** (1:1 molar ratio) a) before and b) after 30 d of heating at 70 ºC, and c) [2]**PR** (mixture of $\mathbf{1}^{4+}$ and **DN38C10** (1:1 molar ratio)); c and uc in parentheses denote complexed as metastable/pseudorotaxane and uncomplexed, respectively.

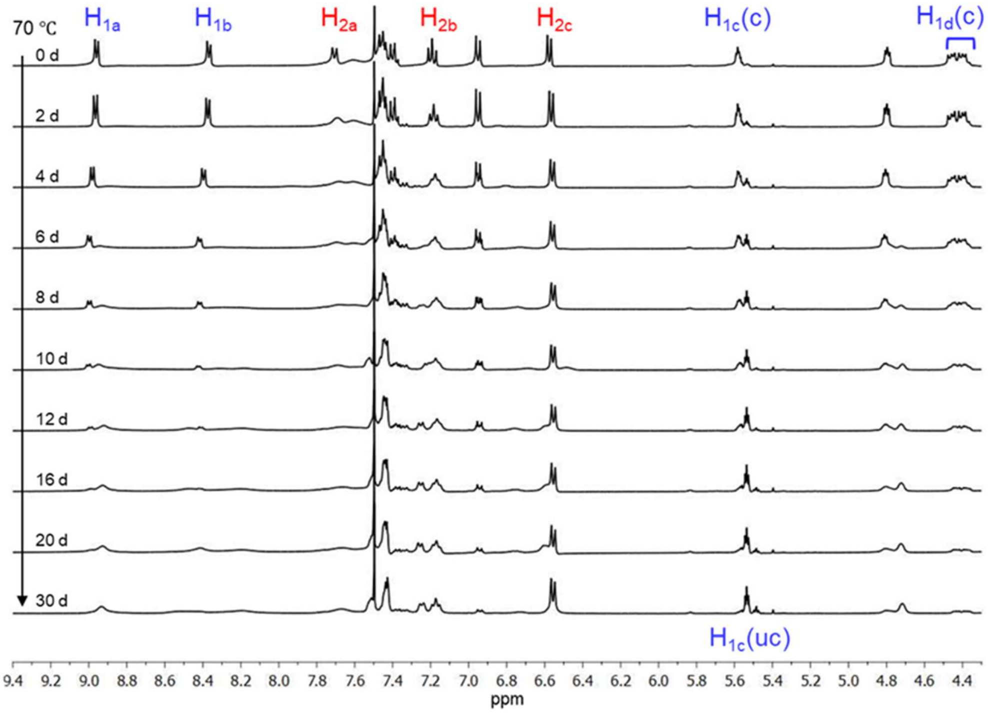

Supplementary Figure 42. [3]**MSR** transformation into [2]**PR** *via* thermal-triggered ring-exchange. Representative partial ^{1}H NMR spectra (400 MHz, $CD_3CN/CDCl_3$ = 1:1, v/v, 3.5 mM) of a 1:1 mixture of [3]**MSR** and **DN38C10** through the heating process at 70 ºC from 0 to 30 d; c and uc in parentheses denote complexed as metastable rotaxane and uncomplexed, respectively.

Ring-exchange from [3]MSR to [3]pseudorotaxane

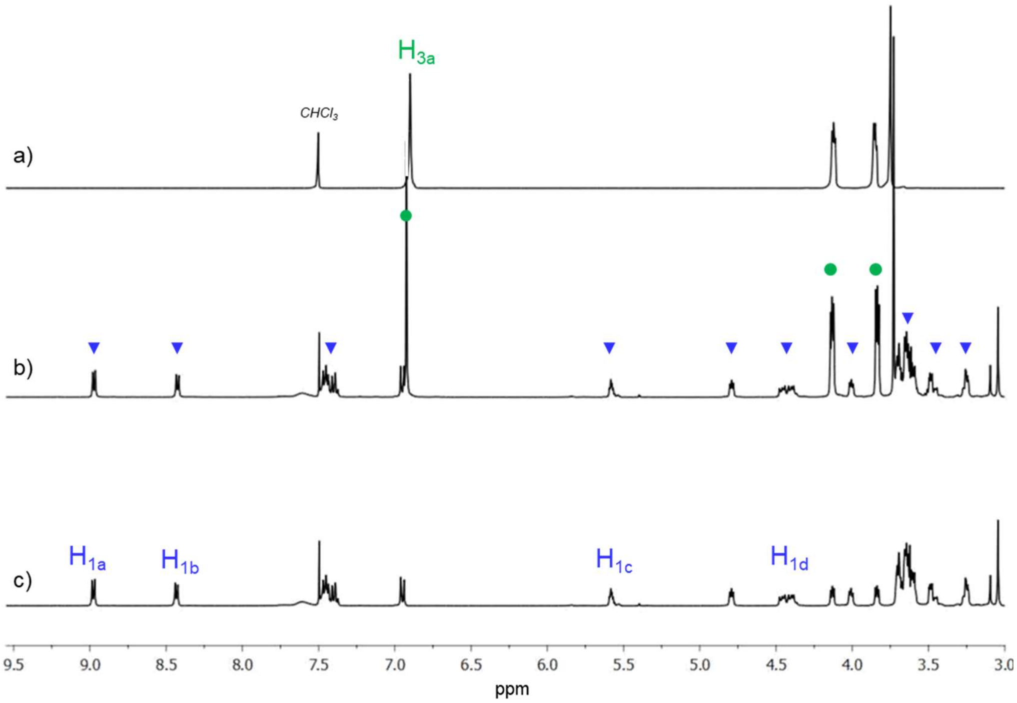

Supplementary Figure 43. Partial ¹H NMR spectra (400 MHz, CD_3CN / $CDCl_3$ = 1:1 (v/v), 3.5 mM). a) **DB24C8**, b) a 1:1 mixture of [3]**MSR** and **DB24C8**, and c) [3]**MSR**. The markers on b) shows the origin of the signals: **DB24C8** (green) and [3]**MSR** (blue).

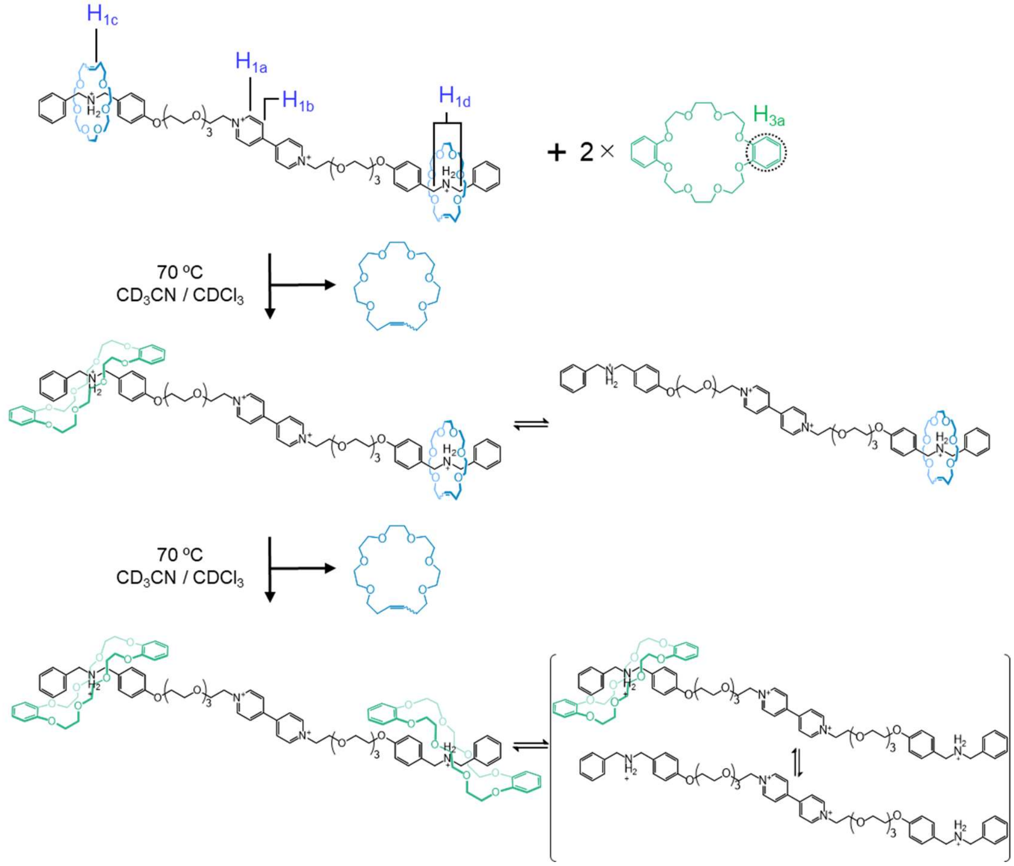

Supplementary Scheme 7. [3]**PR** formation from [3]**MSR** via thermal-triggered ring-exchange

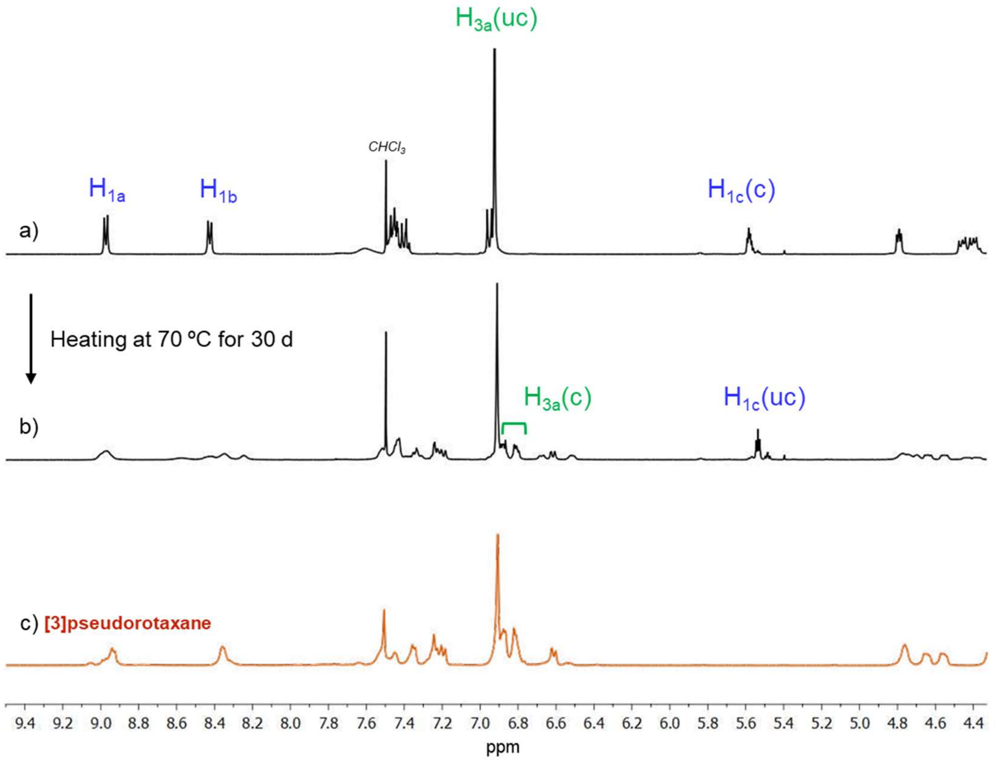

Supplementary Figure 44. Partial ^{1}H NMR spectra (400 MHz, dry CD_3CN / $CDCl_3$ = 1:1, v/v, 3.5 mM). Mixture of [3]**MSR** and **DB24C8** (1:1 molar ratio) a) before and b) after 30 d of heating at 70 ºC, and c) [3]pseudorotaxane (mixture of $\mathbf{1}^{4+}$ and **DB24C8** (1:1 molar ratio)); c and uc in parentheses denote complexed as metastable/pseudorotaxane and uncomplexed, respectively.

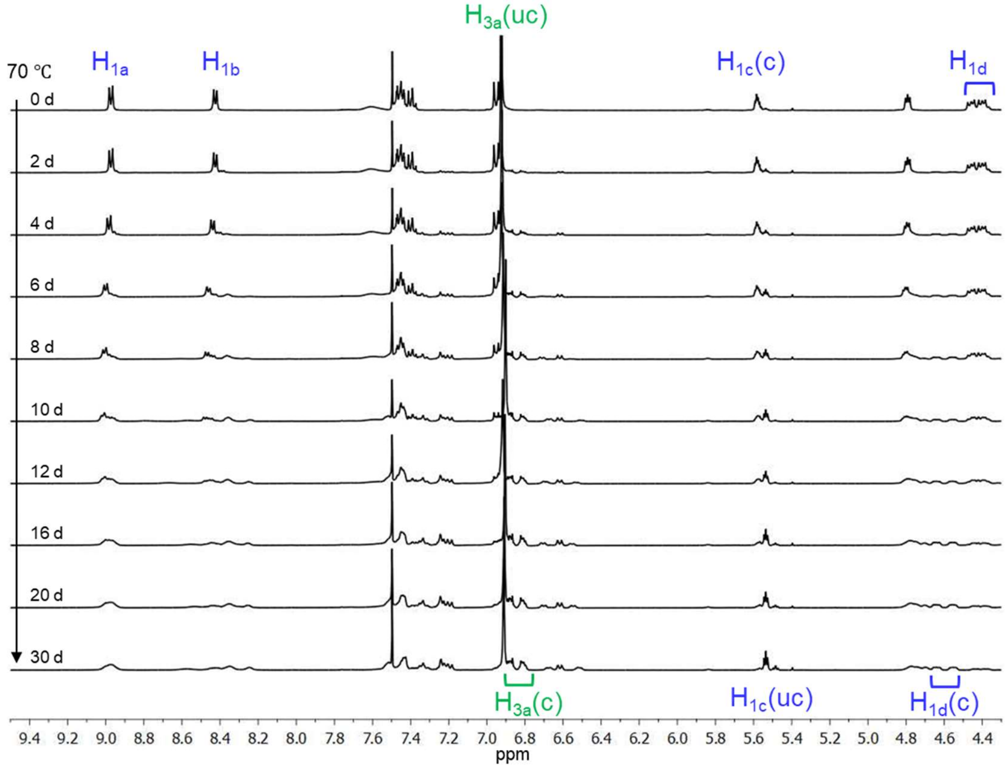

Supplementary Figure 45. [3]**MSR** transformation into [3]**PR** *via* thermal-triggered ring-exchange. Representative partial ^{1}H NMR spectra (400 MHz, $CD_3CN/CDCl_3$ = 1:1, v/v, 3.5 mM) of a 1:2 mixture of [3]**MSR** and **DB24C8** through the heating process at 70 ºC from 0 to 30 d; c and uc in parentheses denote complexed as metastable/pseudorotaxane and uncomplexed, respectively.

Ring-exchange from [3]MSR to [4]pseudorotaxane

The stopper function of [3]**MSR** preventing $\mathbf{1}^{4+}$ from forming **PR**s with free rings was confirmed by ^{1}H NMR studies. A solution containing [3]**MSR** (3.5 mM), **DN38C10**, and **DB24C8** (1:1:2 mol ratio) in $CD_3CN/CDCl_3$ (1:1, v/v) was prepared. In the collected ^{1}H NMR spectrum, we observed all peaks belonging to 3 components ([3]**MSR**, **DN38C10**, and **DB24C8**) overlapped, which indicates all components co-exist without forming any pseudorotaxanes (Supplementary Figure 47). However, protons H_{1a} and H_{1b} shifted upfield ($\Delta\delta$ = 0.03 and 0.08 ppm, respectively), which suggests that the naphthalene moieties of **DN38C10** externally interact with the bipyridinium core of [3]**MSR**.[10],[11] In fact, the prepared NMR solution showed pale pinkish color due to this external interaction of **DN38C10** (Supplementary Figure 46). This NMR sample was stored at room temperature for 30 d and we observed no significant changes in the ^{1}H NMR spectrum over this period (Supplementary Figure 48). This result indicates the sample is very stable, with no reaction occurring at room temperature.

Next, the system was heated to perform the conversion from [3]**MSR** to [4]**PR** *via* ring-exchange on $\mathbf{1}^{4+}$. We prepared a solution of [3]**MSR** (3.5 mM), **DN38C10**, and **DB24C8** (1:1:2 mol ratio) in dry $CD_3CN/CDCl_3$ (1:1, v/v) in an NMR tube and tightly capped it under N_2 protection.We heated this sample at 70 ºC up to 30 d and periodically analyzed it by ^{1}H NMR spectroscopy at 25 ºC. Upon heating, the solution color gradually changed to red implying the growth of species in which **DN38C10** is threaded by $\mathbf{1}^{4+}$ at its BIPY core (Supplementary Figure 46). The NMR spectra before and after 30 d of heating were compared with that of [4]**PR** in Supplementary Figure 49, and representative spectra during the heating process are shown in Supplementary Figure 50. Thermal-triggered dissociation was confirmed by the appearance of free *cis/trans*-**22C6.** Proton H_{1b} shifted upfield *e.g. trans* H_{1b} at 5.58 ppm displayed a $\Delta\delta$ = 0.04 ppm, which agrees with the dissociation behavior of [3]**MSR** analyzed in previous sections. Despite the dissociation of [3]**MSR**, no signals assigned to free $\mathbf{1}^{4+}$ were observed in the NMR spectrum after 30 d of heating. Instead, the signal splitting and chemical shifts suggesting self-assembly of the [4]**PR** structure were observed (see the characterization of [4]**PR**). It is worth mentioning that the *cis/trans*-**22C6** ring does not interfere with the assembly of [4]**PR**. Also, kinetic analysis of the disassembly of **22C6** in this system showed the dissociation process was identical to that of simple [3]**MSR**, meaning that **DN38C10** and **DB24C8** rings do not interfere with the dissociation of [3]**MSR**. In summary, the conversion of [3]**MSR** into [4]**PR**, *via* a two-by-three ring-exchange (**22C6** by **DN38C10** and **DB24C8**) on $\mathbf{1}^{4+}$, is triggered on-demand by the application of a thermal stimulus.

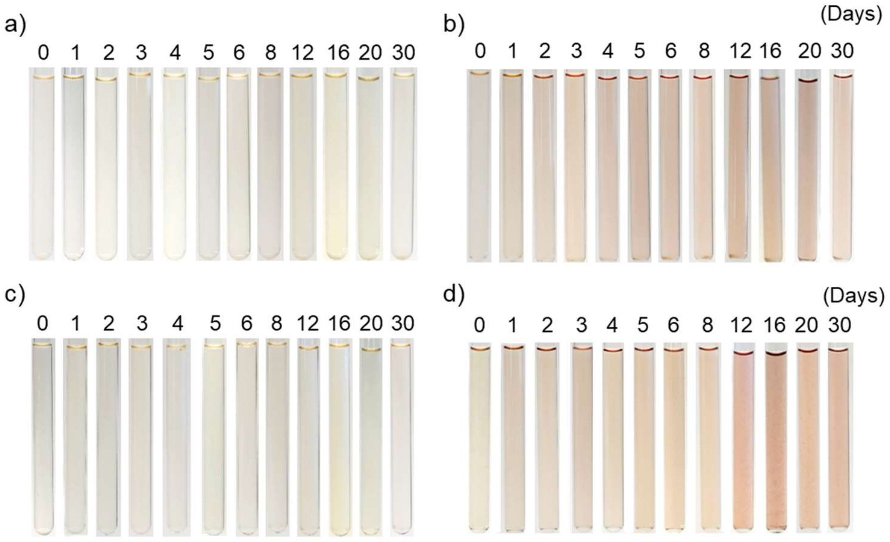

Supplementary Figure 46. Pictures of NMR samples heated at 70 ºC over 30 d for ring-exchange tests; a) [3]**MSR**, b) [3]**MSR**/**DN38C10** (1:1), c) [3]**MSR**/**DB24C8** (1:2), and d) [3]**MSR**/**DN38C10**/**DB24C8** (1:1:2 in molar ratio) in $CD_3CN/CDCl_3$ (1:1, v/v, 3.5 mM).

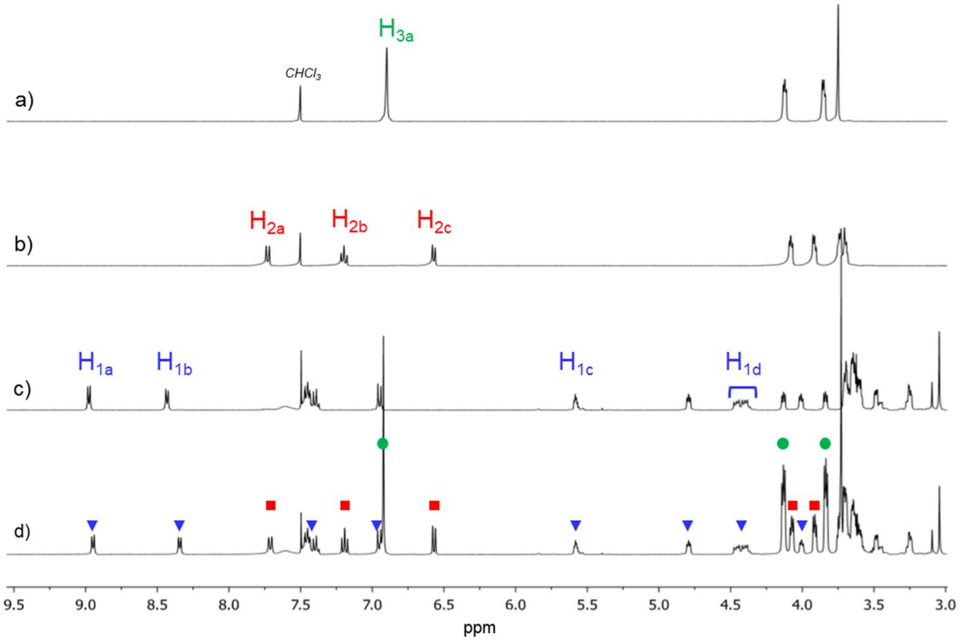

Supplementary Figure 47. Partial ^{1}H NMR spectra (400 MHz, CD_3CN / $CDCl_3$ = 1:1 (v/v), 3.5 mM). a) **DB24C8**, b) **DN38C10**, c) [3]**MSR**, and d) a 1:1:2 mixture of [3]**MSR**, **DN38C10** and **DB24C8**. The markers on d) shows the origin of the signals: **DB24C8** (green), **DN38C10** (red) and [3]**MSR** (blue).

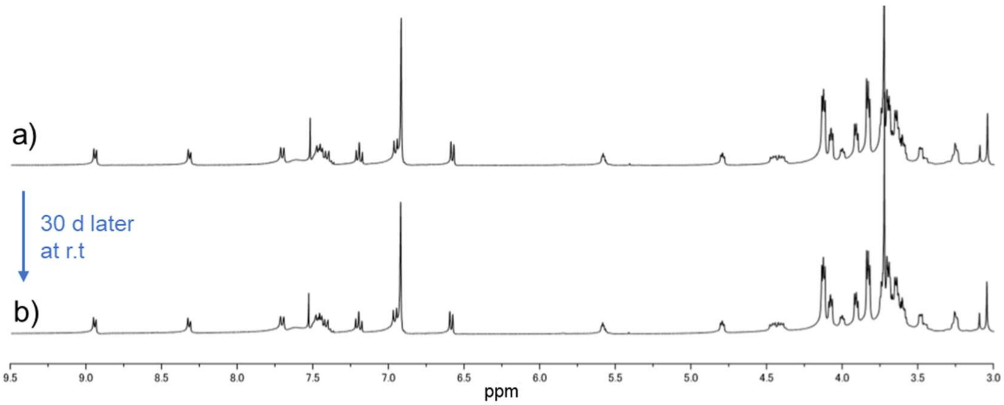

Supplementary Figure 48. Stability test of [3]**MSR**. Particial ^{1}H NMR spectrum (400 MHz, 298 K, $CD_3CN/CDCl_3$ = 1:1, v/v) of [3]**MSR** (3.5 mM), **DN38C10** and **DB24C8** (1:1:2 mol ratio) a) before and b) after stored at room temperature for 30 d.

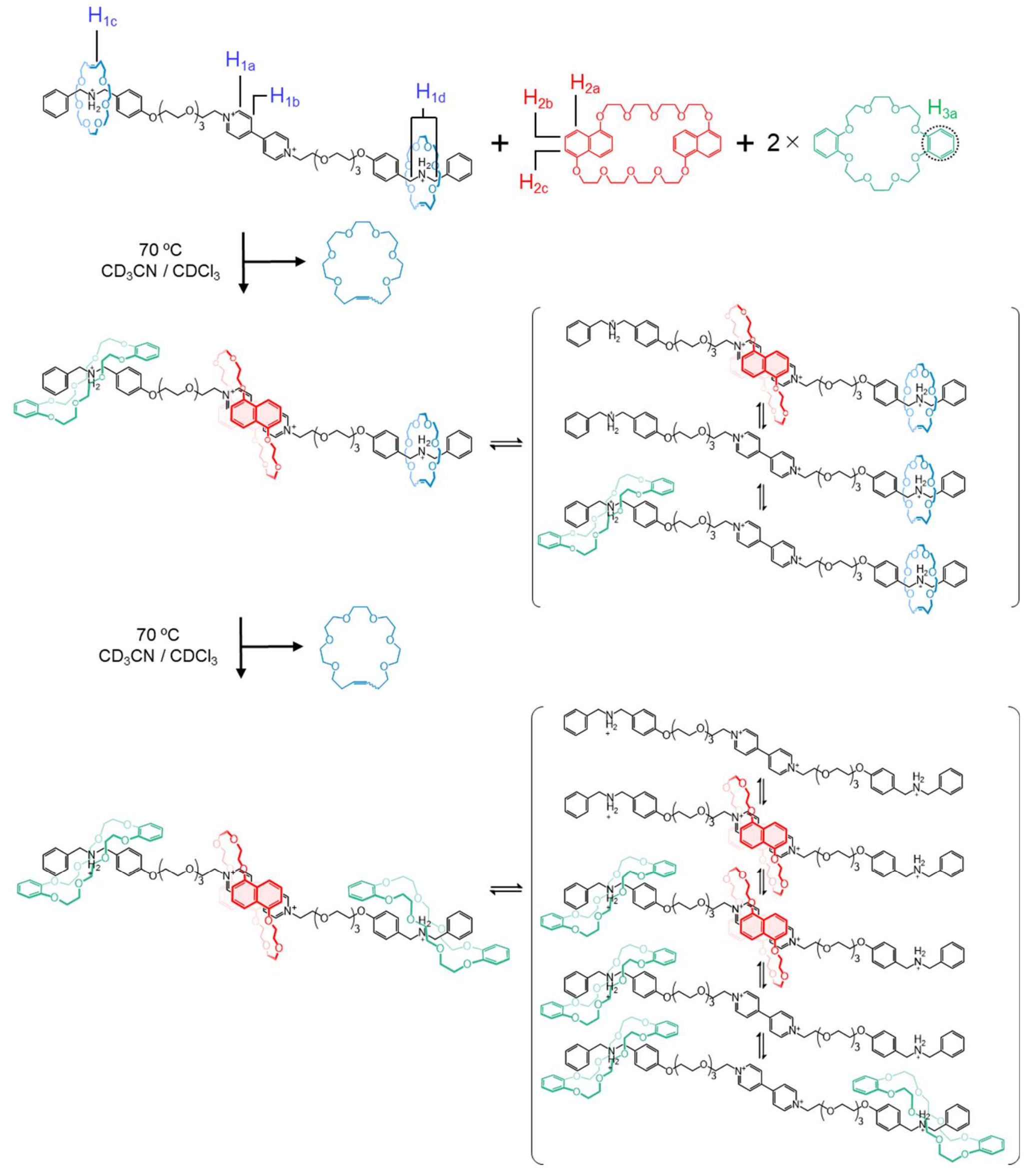

Supplementary Scheme 8. [4]**PR** formation from [3]**MSR** via thermal triggered ring exchange

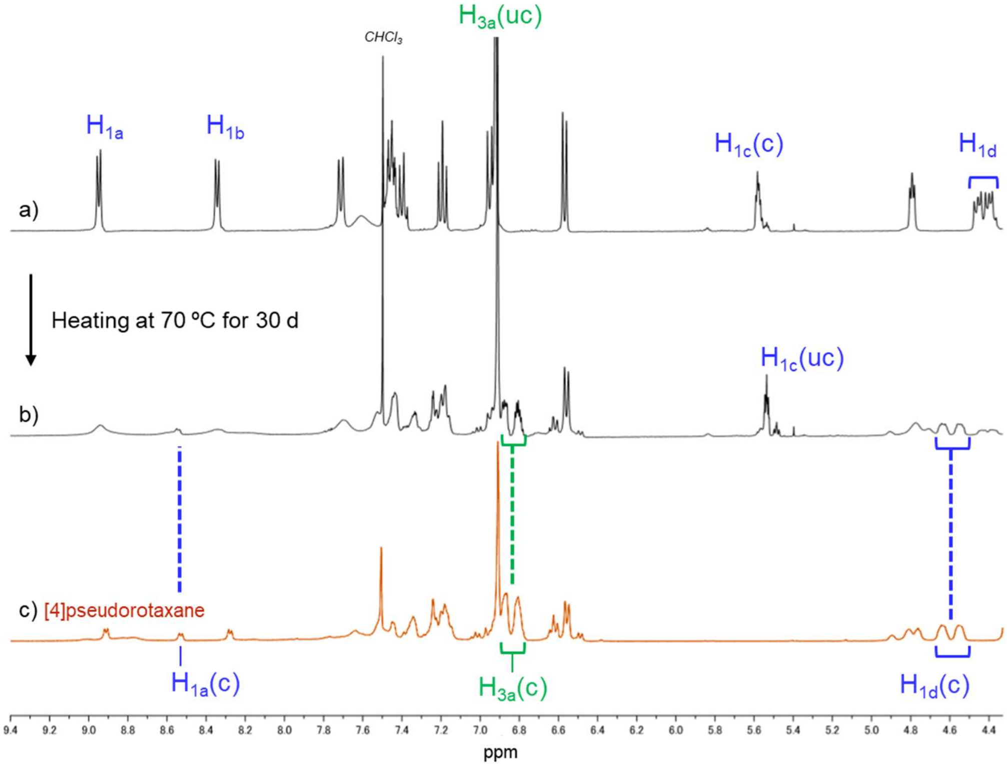

Supplementary Figure 49. Partial ^{1}H NMR spectra (400 MHz, dry CD_3CN / $CDCl_3$ = 1:1, v/v, 3.5 mM). Mixture of [3]**MSR**, **DN38C10**, **DB24C8** (1:1:2 molar ratio) a) before and b) after 30 d of heating at 70 ºC, and c) [4]**PR** (mixture of $\mathbf{1}^{4+}$, **DN38C10**, and **DB24C8** (1:1:2 molar ratio)); c and uc in parentheses denote complexed as metastable/pseudorotaxane and uncomplexed, respectively.

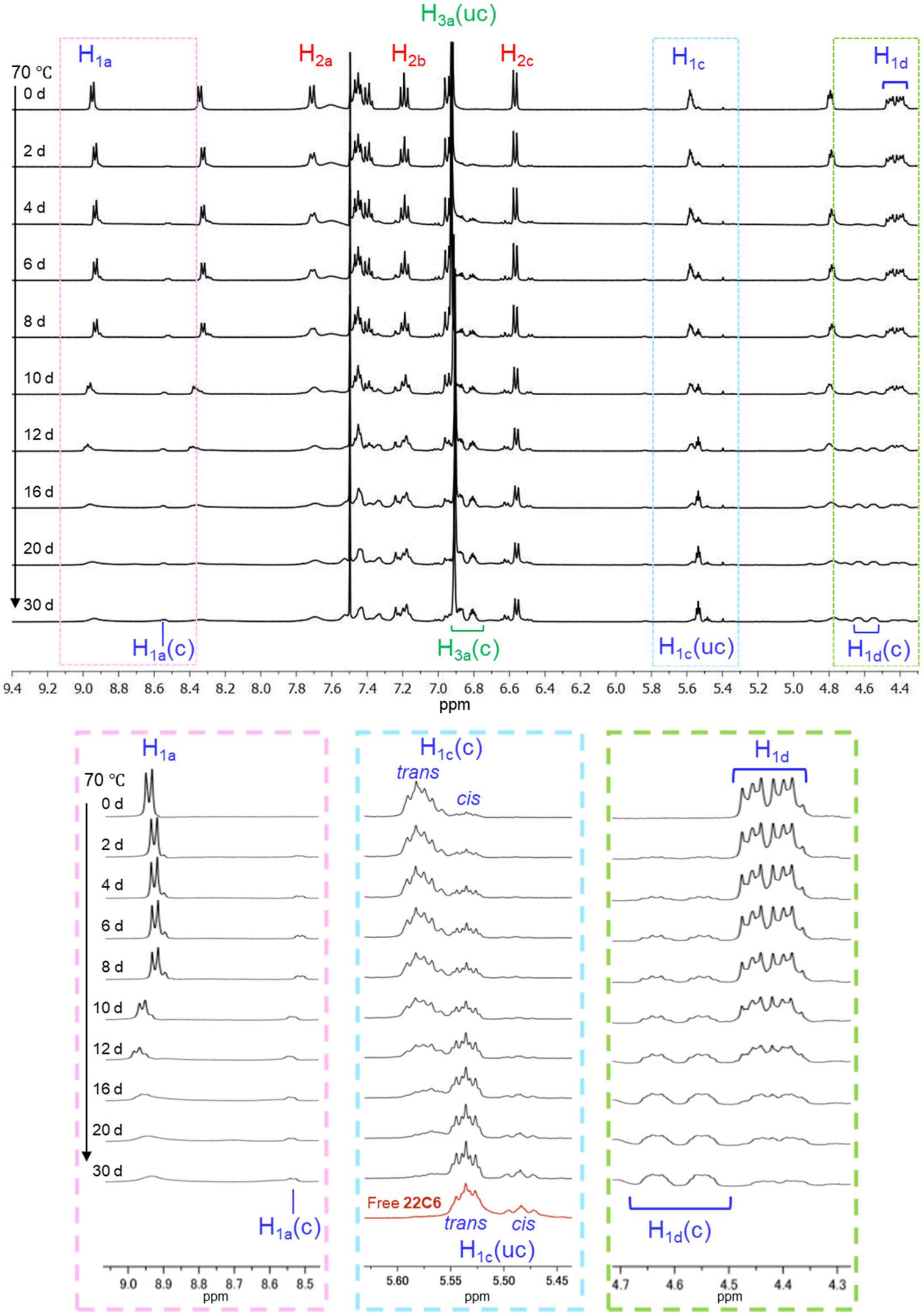

Supplementary Figure 50. [3]**MSR** conversion into [4]**PR** *via* thermal trigger (70 ºC). Representative partial ^{1}H NMR spectra (400 MHz, $CD_3CN/CDCl_3$ = 1:1, v/v, 3.5 mM) of a 1:1:2 mixture of [3]**MSR**, **DN38C10**, and **DB24C8** through the heating process at 70 ºC from 0 to 30 d; c and uc in parentheses denote complexed as metastable/pseudorotaxane and uncomplexed,

respectively.

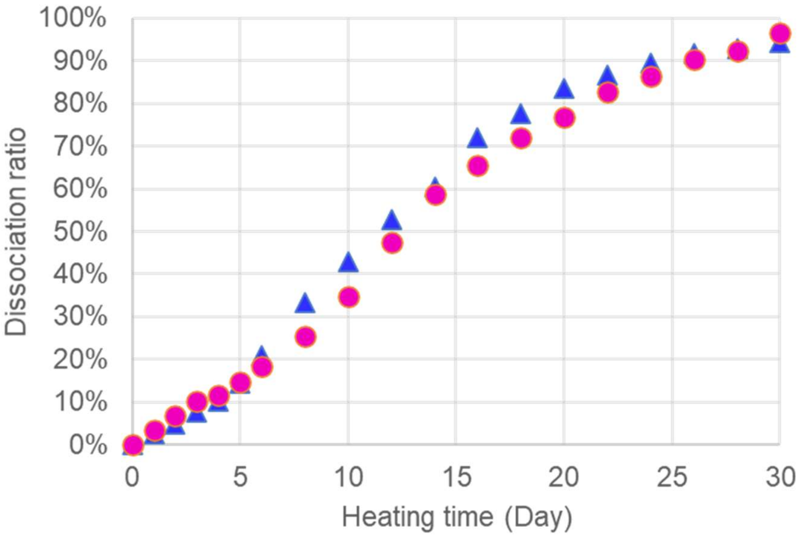

Supplementary Figure 51. Kinetic analysis of **22C6** release *via* dissociation of [3]**MSR** in a solution of [3]**MSR**, **DN38C10**, and **DB24C8** (1:1:2 mol ratio) in $CD_3CN/CDCl_3$ (1:1, v/v, 3.5 mM) (pink circles). The analysis was conducted using the integration of proton H_{1c}. The blue triangles are control data of simple [3]**MSR** shown in Supplementary Figure **37**.

Analysis of color change over transformation from [3]MSR to [4]pseudorotaxane

The heating-time-dependent color change of the preprogrammed system was investigated by UV-vis spectroscopy. Before preprogrammed system prepared the previous section, we started the analysis of a simple system containing **DB24C8** instead of poly(**DB24C8**) for a fundamental understanding of color change caused by ring-exchange from metastable-to-pseudorotaxane.

As a simple programmed system, we prepared seven independent solutions containing [3]**MSR** (35 mM), **DN38C10**, and **DB24C8** (1:1:2 molar ratio) in dry $CH_3CN/CHCl_3$ (1:1, v/v) in vials with screw caps purged with N_2 gas. When adding **DN38C10** to [3]**MSR** solution, the solution color slightly changed from yellow to orangeish yellow due to the unthreaded CT interaction between bipyridinium core of [3]**MSR** and naphthalene moieties of **DN38C10**, which was confirmed by ^{1}H NMR analysis in the previous section. We kept one sample without heating as control and heated the other six samples at 70 ºC for 2, 4, 6, 8, 14, and 30 days, separately. The heated solutions showed a color change to red, and the longer heated solutions had deeper red color (Supplementary Figure 52a). We did not observe any viscosity increase in the heated samples because this simple system did not contain poly(**DB24C8**)

and could not form a supramolecular network. All seven solutions were diluted to 3 mM based on [3]**MSR** concentration and analyzed by UV-vis spectroscopy.

Supplementary note 2

The absorbance of non-heated control at 486 nm was higher than that of pure [3]**MSR** because of the external complex formation. Depending on the heating time, the absorbance at 486 nm representing the charge-transfer interaction of BIPY core ⊂ naphthalene moieties in pseudorotaxane structure gradually increased to give a summit, although the summit at 486 nm was not observed before heating (Supplementary Figure 52b). To confirm the observed absorbance increase is derived from [4]**PR** formation, the sample heated for 30 d was evaporated and then prepared as a solution in $CD_3CN/CDCl_3$ (1:1, v/v) for ^{1}H NMR analysis. The collected ^{1}H NMR data (Supplementary Figure 53) showed both dissociation of [3]**MSR** and formation of [4]**PR**, indicating that the thermal-triggered color change was caused by ring-exchange from [3]**MSR** to [4]**PR**.

To confirm the observed heating-time-dependent solution color change was performed in a programmed manner, we conducted a quantitative investigation into the relationship between collected absorbance data and the existence ratio of [3]**MSR** and its related dissociated species shown in Supplementary Figure **38**b. Upon heating, [3]**MSR**, which existed as an external complex with **DN38C10**, dissociated and gave three pseudorotaxane species having bipyridinium core ⊂ **DN38C10** structure that is the source of absorbance at 486 nm: [2]**PR** (**1**$^{4+}$ ⊂ **DN38C10**, [3]**PR** ((**22C6** and **DB24C8**) ⊃ **1**$^{4+}$ ⊂ **DN38C10**, and [4]**PR** ((**DB24C8**)$_2$ ⊃ **1**$^{4+}$ ⊂ **DN38C10**)). Excluding the contribution of colorless **DB24C8** and dissociated **22C6**, the observed absorbance at 486 nm can be represented as Equation (20) as below;

$A_{obs(n)} = A_{PR(n)} + A_{EC(n)}$ …Equation (20)

Where,
(n): Heating days at 70 ºC
A_{obs}: Observed absorbance at 486 nm in Supplementary Figure 52b
A_{PR}: Absorbance factor in A_{obs} derived from PRs having bipyridinium core ⊂ **DN38C10** structure
A_{EC}: Absorbance factor in A_{obs} derived from the external complex between [3]**MSR** and **DN38C10**

Here, $A_{EC(n)}$ can be expressed by Equation (21), because [3]**MSR** and related external complex are consumed upon heating, and their amount can be estimated by the existence ratio plot in Supplementary **Figure 38**. Furthermore, Equation (22) holds because no pseudorotaxanes exist in the

programmed system before heating.

$A_{EC(n)} = A_{EC(0)} \times X_{[3]MSR(n)}$ …Equation (21)

$A_{EC(0)} = A_{obs(0)}$ …Equation (22)

Where,

$X_{[3]MSR(n)}$: Existence ratio of [3]**MSR** heated at 70 ºC for n days, which is read in Supplementary **Figure 38**b

Combining Equation (20) to (22), Equation (23) that can extract the absorbance from pseudorotaxanes is obtained.

$A_{PR(n)} = A_{obs(n)} - A_{obs(0)} \times X_{[3]MSR(n)}$ …Equation (23)

Once all [3]**MSR** dissociate losing at least one **22C6**, all dissociated species get to have BIPY core ⊂ **DN38C10** structures. This means the formation of BIPY core ⊂ **DN38C10** structure and related red color growth is saturated at this point. Regarding $A_{obs(30)}$ as a saturated point where the absorbance at 486 nm fully consisted of pseudorotaxane structure (BIPY core ⊂ **DN38C10**), we defined absorbance increase ratio as Equation (24) and plotted them against heating time in Supplementary Figure 55.

$Absorbance\ increase\ ratio_{(n)} = \frac{A_{PR(n)}}{A_{obs(30)}}$ …Equation (24)

The resulting plots matched with that of the existence ratio of dissociated [3]**MSR** species provided from

Supplementary **Figure 38**b, which proved the solution color change to red was performed in the expected programmed manner.

Based on the results of the basic programmed system using non-polymeric **DB24C8**, the preprogrammed supramolecular network systems containing poly(**DB24C8**) prepared in the previous section were analyzed identically by UV-vis spectroscopy. We heated 8 samples of system 1 at 70 ºC for 0 (control), 4, 6, 10, 14, 18, 22, and 30 days, separately, and also heated 6 samples of system 2 for 0 (control), 5, 10, 16, 26, and 30 days, separately. In both systems, the initial samples without heating were free-flowing solutions with yellowish-orange color, but they became tough red gel after 30-days of heating. Besides, each system showed gradual changes in terms of both color changes and viscosity increases depending on heating time. For the quantitative analysis of color change, we diluted the

system to solutions (1.7 mM for system 1 and 3.0 mM for system 2), measured their absorbance spectra, and then plotted the collected absorbance increase ratio calculated from Equation (24) in Supplementary Figure 55. The resulting plots matched with the existence ratio of dissociated [3]**MSR** species as well, indicating that supramolecular polymeric systems can output a color change in a programmed manner (viscosity increases are discussed in the next section).

a)

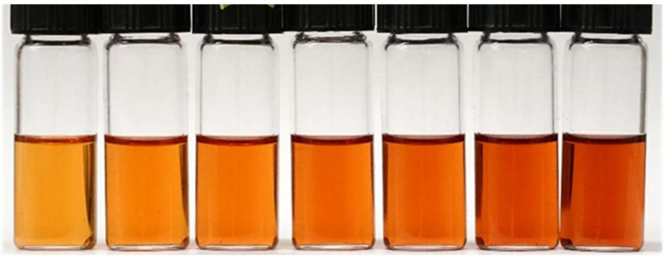

b)

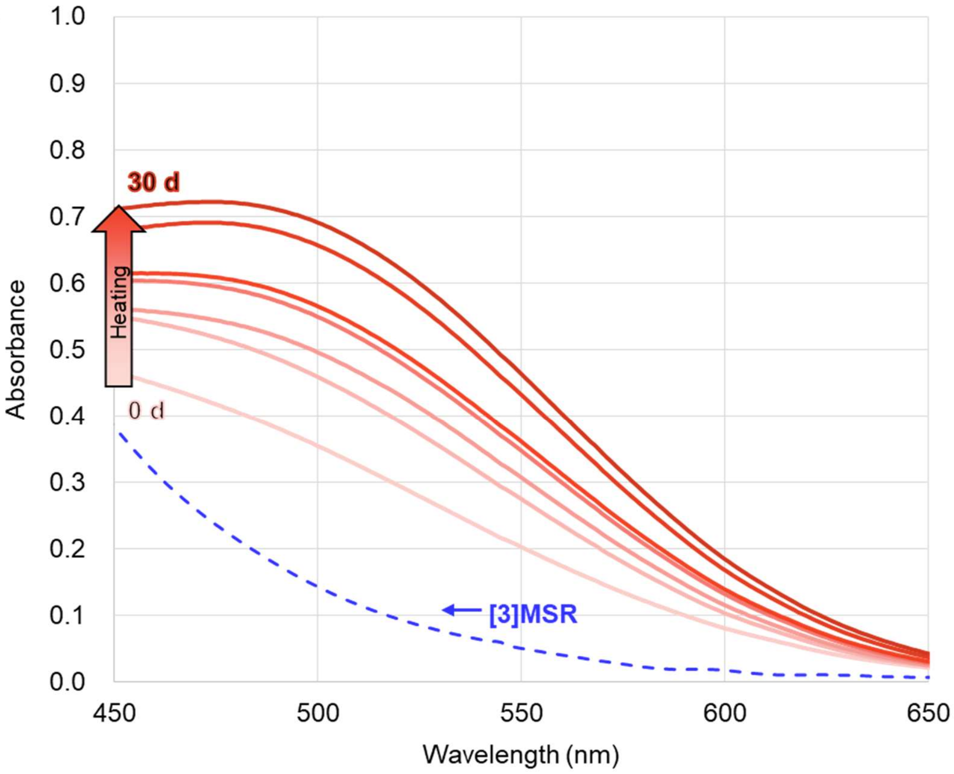

Supplementary Figure 52. a) Pictures of programmed system ([3]**MSR**: **DN38C10**: **DB24C8** = 1: 1: 2 in molar ratio) in $CH_3CN/CHCl_3$ (1:1, v/v) at 5 mM heated at 70 ºC for 0, 2, 4, 6, 8, 14, and 30 d from left to right. b) UV-vis spectra of [3]**MSR** (blue dash line) and programmed system ([3]**MSR**: **DN38C10**: **DB24C8** = 1: 1: 2 in molar ratio) in $CH_3CN/CHCl_3$ (1:1, v/v) at 3 mM heated at 70 ºC for 0, 2, 4, 6, 8, 14, and 30 d (solid reddish lines from bottom to top)

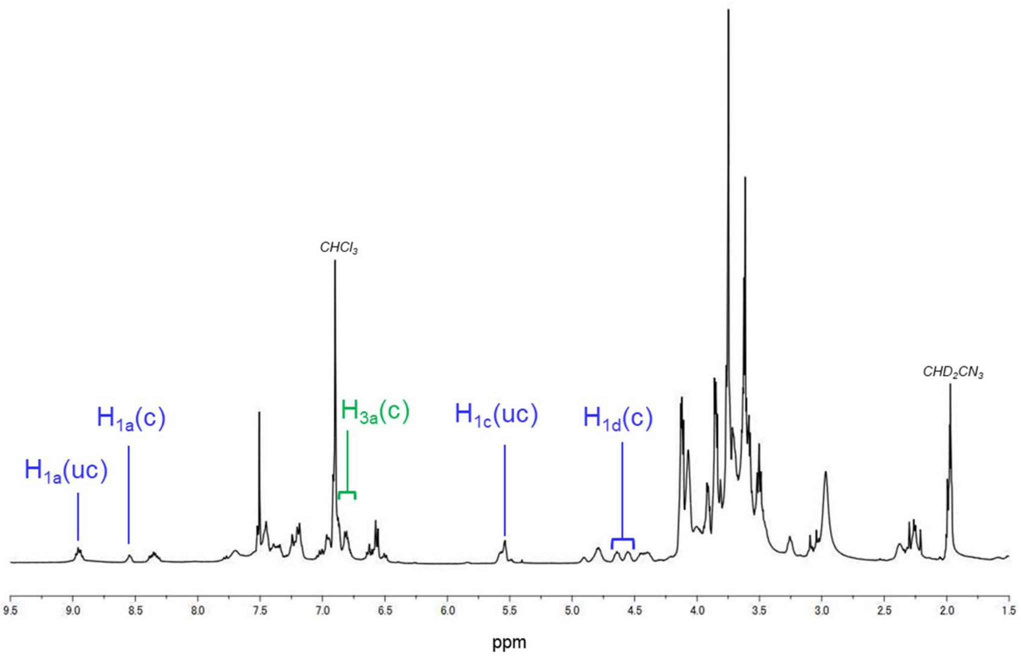

Supplementary Figure 53. Partial ^{1}H NMR spectra (400 MHz, dry CD_3CN / $CDCl_3$ = 1:1, v/v) of the programmed system ([3]**MSR**: **DN38C10**: **DB24C8** = 1: 1: 2 in molar ratio) heated at 70 ºC for 30 d, which was used for UV-vis analysis in Supplementary Figure **52**.

a)

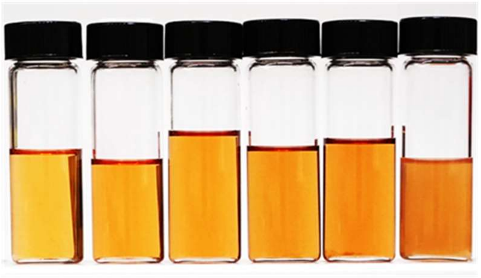

b)

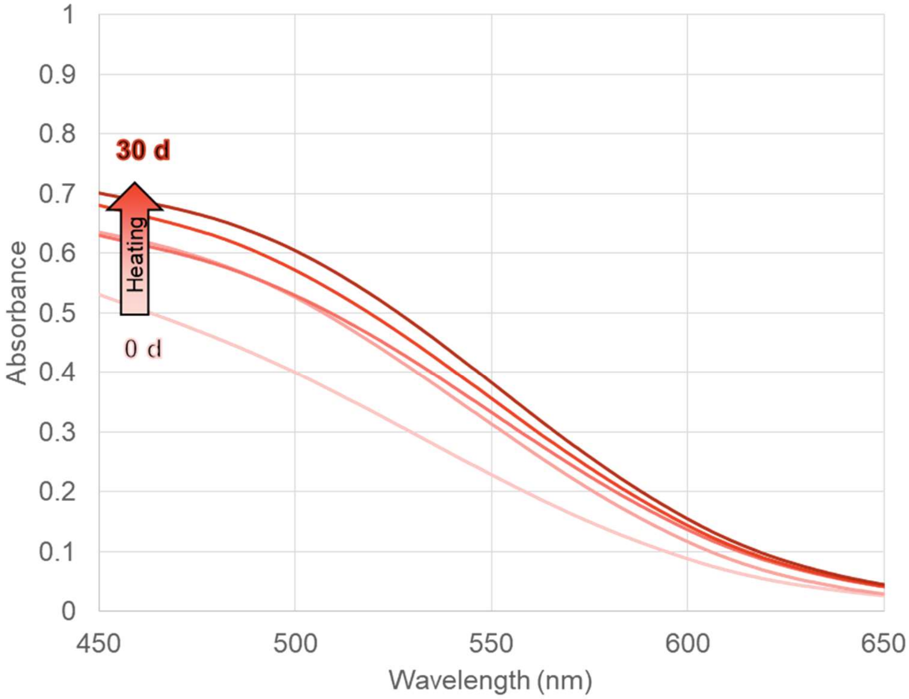

Supplementary Figure 54. a) Pictures of diluted programmed system 2 ([3]**MSR**: **DN38C10**: poly(**DB24C8)** = 1: 1: 2 in molar ratio) in $CH_3CN/CHCl_3$ (1:1, v/v) at 3 mM heated at 70 ºC for 0, 5, 10, 16, 26, and 30 d from left to right. b) UV-vis spectra of the diluted programmed system 2 ([3]**MSR**: **DN38C10**: poly(**DB24C8)** = 1: 1: 2 in molar ratio) in $CH_3CN/CHCl_3$ (1:1, v/v) at 3 mM heated at 70 ºC for 0, 5, 10, 16, 26, and 30 d (redish lines from bottom to top)

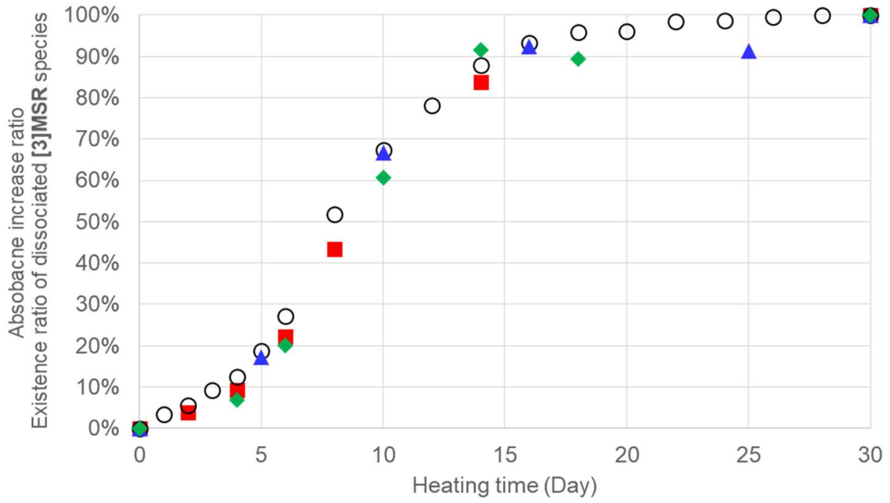

Supplementary Figure 55. Plots of heating-time-dependent absorbance increase ratio of the existence ratio of dissociated of [3]**MSR** (1 – Existence ratio of [3]**MSR**) calculated from Supplementary Figure **38**b (open circles), and the programmed systems ([3]**MSR**: DN38C10: DB24C8 = 1: 1: 2 in molar ratio) (red), system 1 (green), and system 2 (blue).

Synthesis and characterization of poly(DB24C8)

Polymer poly(**DB24C8**) was prepared based on a reported procedure[6] and characterized by ^1H NMR spectroscopy, GPC and MALDI-TOF mass spectrometry (see Supplementary Figure 56 to Supplementary Figure 58). M_w, M_n and PDI values were calculated from the MALDI-TOF MS data using the Polytool software from Bruker.

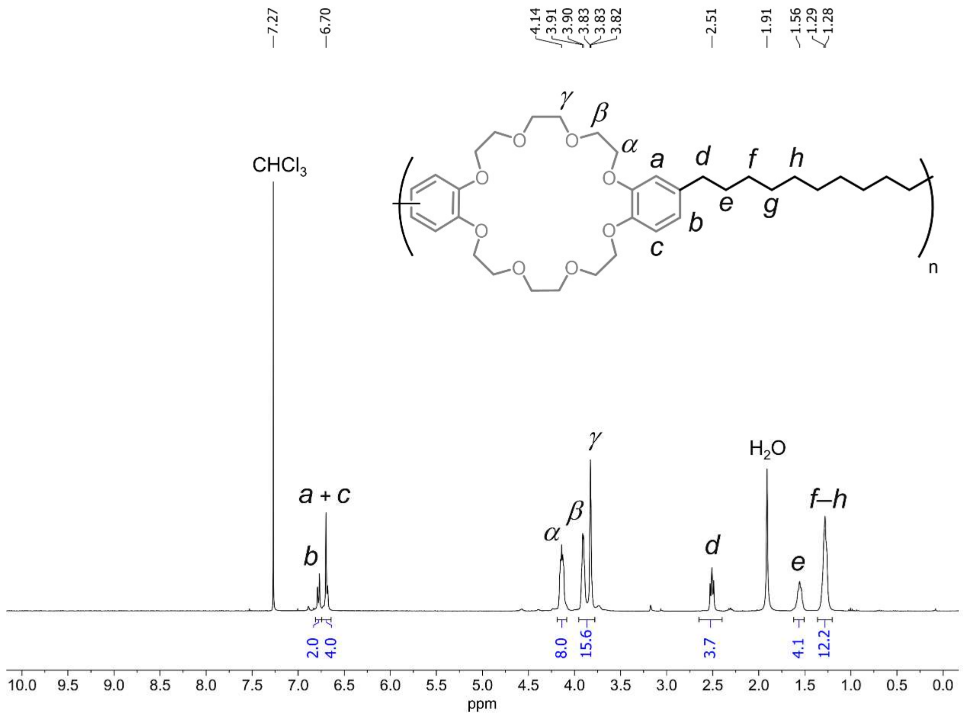

Supplementary Figure 56. ^1H NMR spectrum (400 MHz, $CDCl_3$) of poly(**DB24C8**).

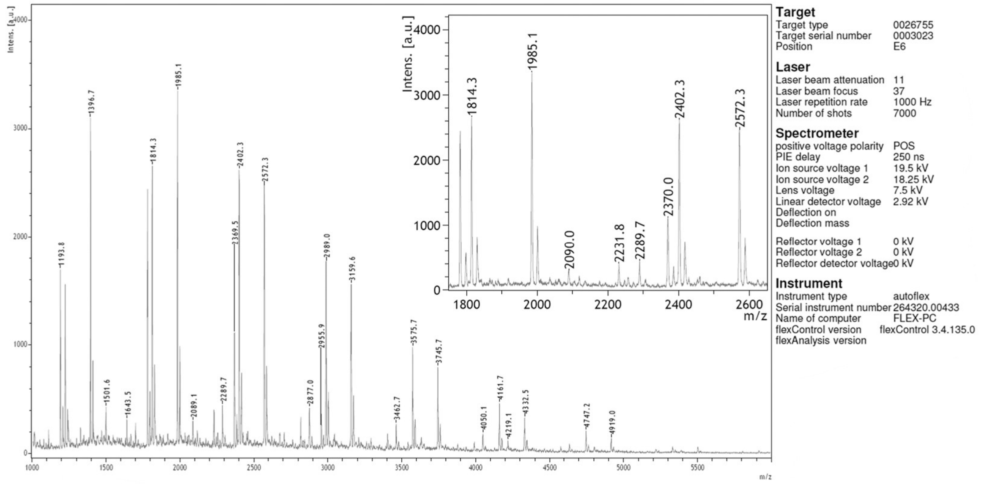

Supplementary Figure 57. MALDI-TOF mass spectrum. 2,5-Dihydroxybenzoic acid used as the matrix.

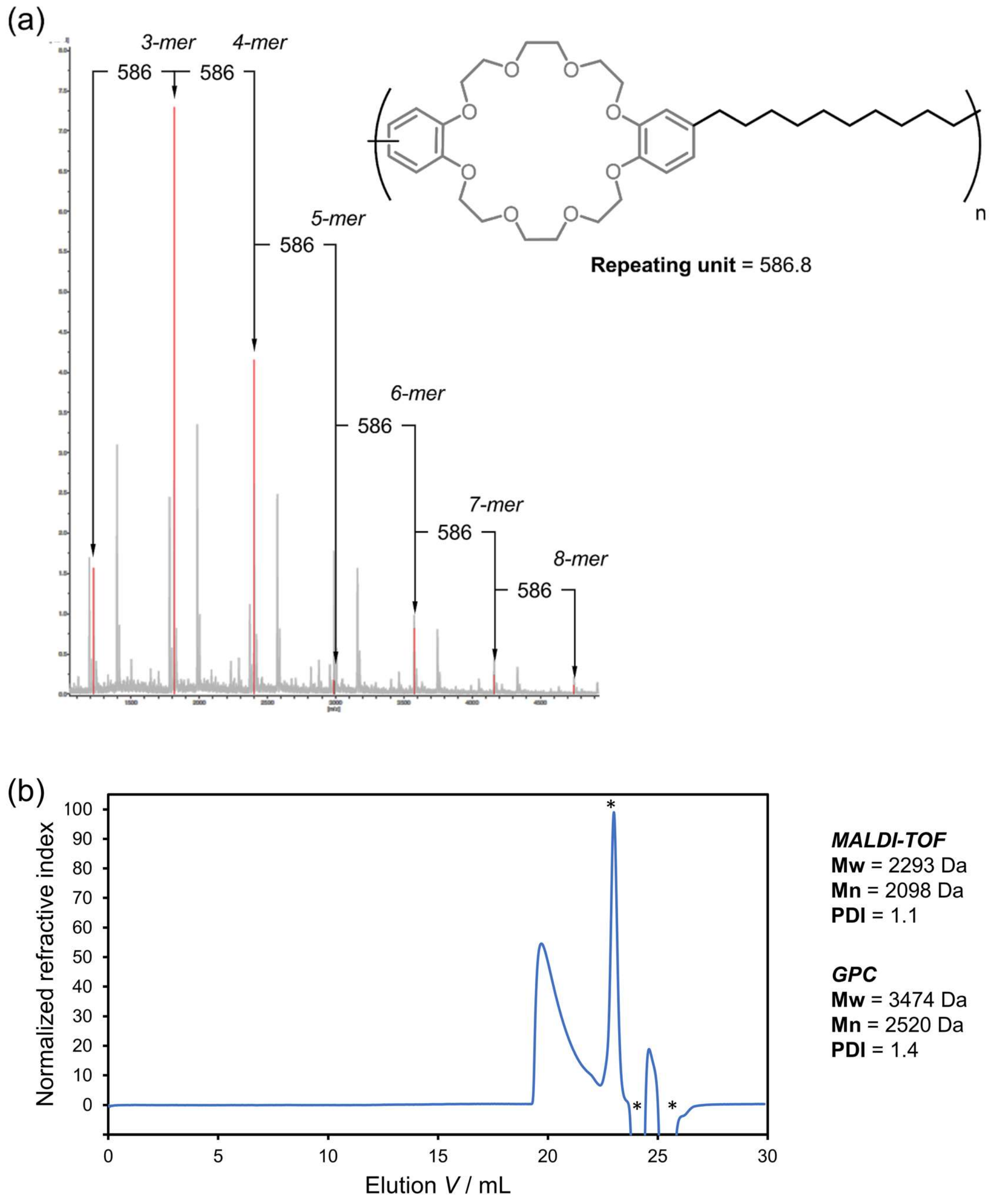

Supplementary Figure 58. (a) Fitted region of the MALDI-TOF mass spectrum. Plot in gray represents experimental data and red lines correspond to peak integrals measured with Polytool. (b) GPC trace of poly(**DB24C8**); [poly(**DB24C8**)] = 1 mg/mL; THF, 35 °C. * denotes injection artifacts. M_n and M_w GPC values reported relative to polystyrene standards.

Gelation of poly(DB24C8) with 1^{4+}

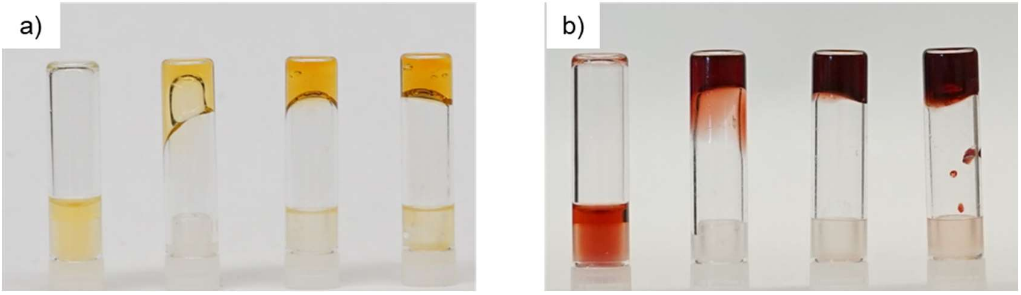

Supplementary Figure 59. Pictures of poly(**DB24C8**) and $\mathbf{1}^{4+}$ (1:2, molar ratio) a) without and b) with 1 equivalent of **DN38C10** in $CH_3CN/CHCl_3$ (1:1, v/v). The concentration of poly(**DB24C8**) is 25, 50, 75, and 100 mM from left to right, respectively.

Preparation of preprogrammed supramolecular network samples

The preprogrammed supramolecular network sample was prepared as a mixture of [3]**MSR**, **DN38C10**, and poly(**DB24C8**) with dry $CH_3CN/CHCl_3$ (1:1, v/v) in a tightly capped vial under the protection of N_2. We prepared 2 systems with different concentrations of molecular components: [3]**MSR**:**DN38C10**:poly(**DB24C8**) = 1:1:24 (12.5 mM) for system 1 and 1:1:2 (50 mM) for system 2. Eight and six independent samples were prepared for system 1 and system 2, respectively. The prepared samples in both systems 1 and 2 were free-flowing solutions with light orange color, which is attributed to weak CT from the electron-rich **DN38C10** ring to the BIPY unit of $\mathbf{1}^{4+}$ through an unthreaded geometry.

Analysis of viscosity change over transformation from [3]MSR to [4]pseudorotaxane

To probe the thermal-triggered viscosity changes of the preprogrammed systems, systems 1 and 2 prepared in the previous section were analyzed by a rheometer. We heated eight samples of system 1 for 0 (control), 4, 6, 10, 14, 18, 22, and 30 days and six samples of system 2 for 0 (control), 10, 16, 20, 26, and 30 days, separately. In both systems, the starting solutions were low viscosity, free-flowing liquids before heating. As the samples heated, we observed an increase in viscosity and transformation to a gel on the 14th day of heating in system 1 and the 10th day of heating in system 2. The rheological analysis of the preprogrammed systems was conducted by the dynamic oscillatory shear measurements (25 mm diameter parallel cone plates with a 100 μm gap) from 1 to 100 rad s^{-1} at 15 ºC under a strain

of 1%. The obtained storage modulus, loss modulus, and complex viscosity are shown in Supplementary Figure 63. Both System 1 and 2 exhibit the clear tendency of increasing complex viscosity depending on heating time.

To get quantitative insights on viscosity increase depending on the heating time, the obtained complex viscosity at 8.11 rad s^{-1} is plotted against the expected existence ratio of free $\mathbf{1}^{4+}$ calculated from Equation (19) in Supplementary Figure 63. The obtained plots show a strong correlation between complex viscosity and the existence ratio of free $\mathbf{1}^{4+}$ in both system 1 and 2, suggesting that the thermal-triggered viscosity increases are caused by the released $\mathbf{1}^{4+}$. This is a reasonable result because $\mathbf{1}^{4+}$ is the only species that can work as a cross-linker for poly(**DB24C8**) among [3]**MSR** and its dissociated species.

To get a deeper understanding of the preprogrammed viscosity increase, we prepared control samples that represent the ideal terminal states of the programmed system where all [3]**MSR** and [2]**MSR** fully converted into $\mathbf{1}^{4+}$. Specifically, [3]**MSR**, **DN38C10**, poly(**DB24C8**), and isolated **22C6** were mixed in $CH_3CN/CHCl_3$ (1:1, v/v) with different concentrations: 1: 1: 24: 2 (molar ratio, 12.5 mM) for system 1 and 1: 1: 2: 2 (molar ratio, 50.0 mM) for system 2. Both mixtures, which formed tough red gels, were analyzed by the dynamic oscillatory shear measurements, and the obtained results are plotted in Supplementary Figure 60 and Supplementary Figure 63. In Supplementary Figure 63, the control sample viscosities lie almost exactly on the extrapolated positions for both programmed system 1 and 2. This means the thermally-triggered viscosity increases of the preprogrammed systems proceeds toward their fully cross-linked states. In summary, the programmed system can increase its viscosity toward that of a fully cross-linked state with longer heating time.

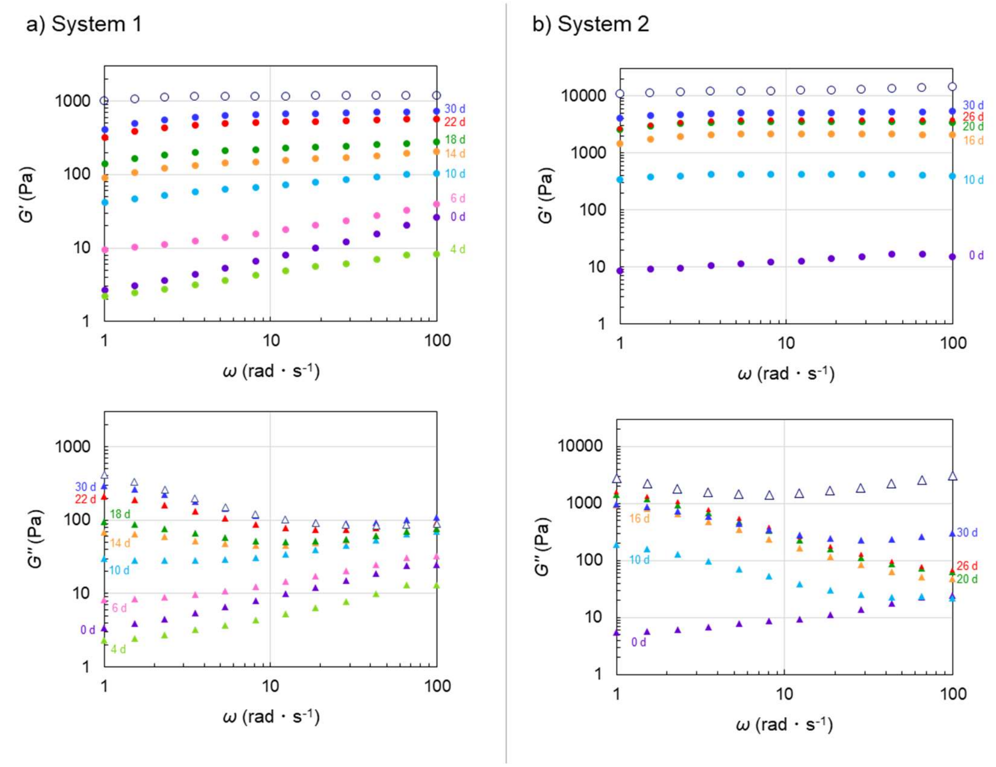

Supplementary Figure 60. Oscillation frequency sweep of a) System 1 and b) System 2: Storage modulus G’ (circle), loss modulus G” (triangle), and complex viscosity (square); close and open symbols for the programmed system and control samples, respectively.

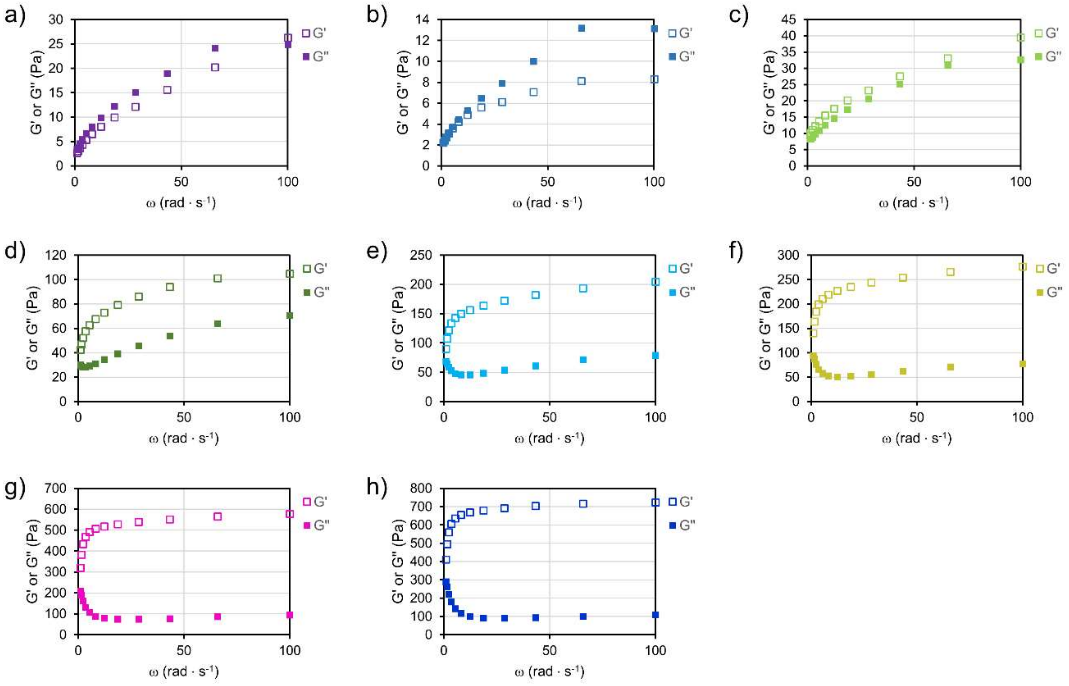

Supplementary Figure 61. G’ and G’’ plots for System 1. Data collected after heating each sample at 70 °C for a) 0 h, b) 4 h, c) 6 h, d) 10 h, e) 14 h, f) 18 h, g) 22 h, h) and 30 h. Experiments a) and b) confirm the liquid-like behaviour of the corresponding materials (G’’ > G’). The viscoelastic properties of all remaining materials, heated for 0 h or more (panels c) to h)), correlate well with the assembly of supramolecular polymer networks (gel-like materials G’ > G’’).

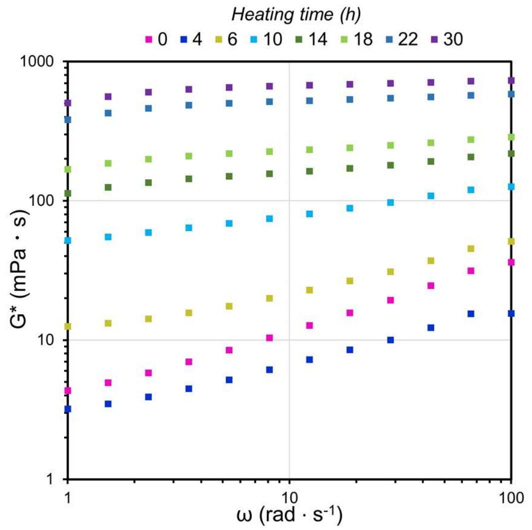

Supplementary Figure 62. G* values for all assembled materials (system 1). G*, interpreted as the stiffness of the materials, increases concomitantly with heating time, which demonstrates the enhancement of the supramolecular networks as more crosslinker is released during thermal treatment.

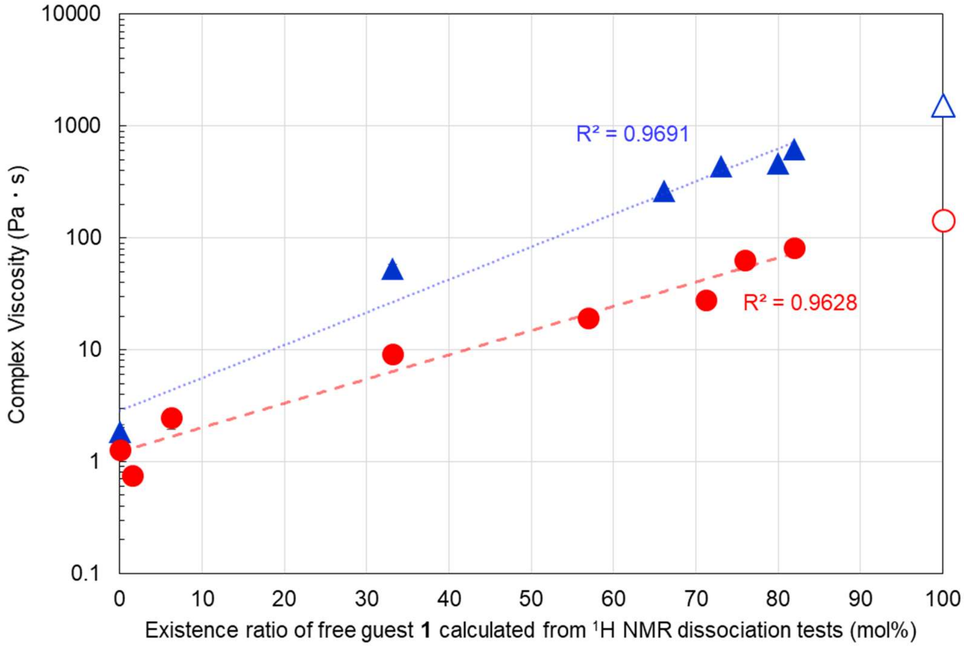

Supplementary Figure 63. Plots of heating-time dependent complex viscosity at 8.11 rad s^{-1} of System 1 (red) and System 2 (blue) against existence ratio of free guest **1** calculated from Equation (19); Programmed samples (close symbol) and control samples (open symbol). Linear fits for both data sets are included (dashed line).

Supplementary references

1. Shirude, P. S., Kumar, V. A. & Ganesh, K. N. BisPNA Targeting to DNA: Effect of Neutral Loop on DNA Duplex Strand Invasion by aepPNA-N7G/aepPNA-C Substituted Peptide Nucleic Acids. *Eur. J. Org. Chem.* **2005**, 5207–5215 (2005).
2. Juricek, M. *et al.* An ExBox [2]catenane. *Chem. Sci.* **5**, 2724–2731 (2014).
3. Vukotic, V. N. *et al.* Mechanically Interlocked Linkers inside Metal–Organic Frameworks: Effect of Ring Size on Rotational Dynamics. *J. Am. Chem. Soc.* **137**, 9643–9651 (2015).
4. Bruns, C. J., Basu, S. & Stoddart, J. F. Improved synthesis of 1,5-dinaphtho[38]crown-10. *Tetrahedron Lett.* **51**, 983–986 (2010).
5. T. Buchen; P. Gütlich; K.H. Sugiyarto; H.A. Goodwin. Bis[2]catenanes and a Bis[2]rotaxane-Model Compounds for Polymers with Mechanically Interlocked Components. *Chem. Eur. J.* **49**, 1134 (1996).
6. Soto, M. A. & Tiburcio, J. Self-assembly of a supramolecular network with pseudo-rotaxane cross-linking nodes and its transformation into a mechanically locked structure by rotaxane formation *Chem. Commun.* **52**, 14149–14152 (2016).
7. Li, H.-G. & Wang, G.-W. Liquid-Assisted One-Pot Mechanosynthesis and Properties of Neutral Donor–Acceptor [2]Rotaxanes. *J. Org. Chem.* **82**, 6341–6348 (2017).
8. Fernando, I. R., Bairu, S. G., Ramakrishna, G. & Mezei, G. Single-color pseudorotaxane-based temperature sensing. *New J. Chem.* **34**, 2097–2100 (2010).
9. http://supramolecular.org. Accessed June 1, 2022.
10. Wei, P., Wang, H., Jie, K. & Huang, F. Taco complex-templated highly regio- and stereo-selective photodimerization of a coumarin-containing crown ether. *Chem. Commun.* **53**, 1688–1691 (2017).
11. Christinat, N., Scopelliti, R. & Severin, K. Boron-based rotaxanes by multicomponent self-assembly. *Chem. Commun.* 3660–3662 (2008).
